# Solid-Phase Synthesis of Caged Luminescent Peptides via Side Chain Anchoring

**DOI:** 10.1021/acs.bioconjchem.3c00381

**Published:** 2023-12-06

**Authors:** Daan Sondag, Jurriaan J. A. Heming, Dennis W. P. M. Löwik, Elena Krivosheeva, Denise Lejeune, Mark van Geffen, Cornelis van’t Veer, Waander L. van Heerde, Marjolijn C. J. Beens, Brian H. M. Kuijpers, Thomas J. Boltje, Floris P. J. T. Rutjes

**Affiliations:** † Institute for Molecules and Materials, Radboud University, Nijmegen 6525 AJ, The Netherlands; ‡ Enzyre BV, Novio Tech Campus, Transistorweg 5-i, Nijmegen 6534 AT, The Netherlands; § Department of Haematology, Radboud University Medical Centre, Nijmegen 6525 GA, The Netherlands; ∥ Haemophilia Treatment Centre, Nijmegen Eindhoven Maastricht (HTC-NEM), Nijmegen 6525 GA, The Netherlands; ⊥ Symeres BV, Kerkenbos 1013, Nijmegen 6546 BB, The Netherlands

## Abstract

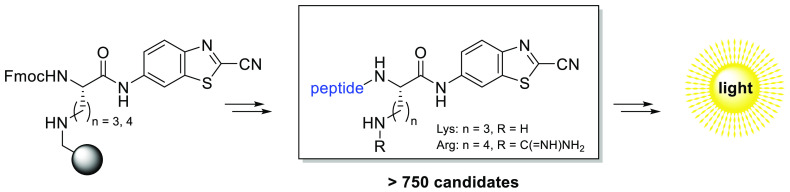

The synthesis of caged luminescent peptide substrates remains challenging, especially when libraries of the substrates are required. Most currently available synthetic methods rely on a solution-phase approach, which is less suited for parallel synthesis purposes. We herein present a solid-phase peptide synthesis (SPPS) method for the synthesis of caged aminoluciferin peptides via side chain anchoring of the P_1_ residue. After the synthesis of a preliminary test library consisting of 40 compounds, the synthetic method was validated and optimized for up to >100 g of resin. Subsequently, two separate larger peptide libraries were synthesized either having a P_1_ = lysine or arginine residue containing in total 719 novel peptide substrates. The use of a more stable caged nitrile precursor instead of caged aminoluciferin rendered our parallel synthetic approach completely suitable for SPPS and serine protease profiling was demonstrated using late-stage aminoluciferin generation.

## Introduction

**Scheme 1:**
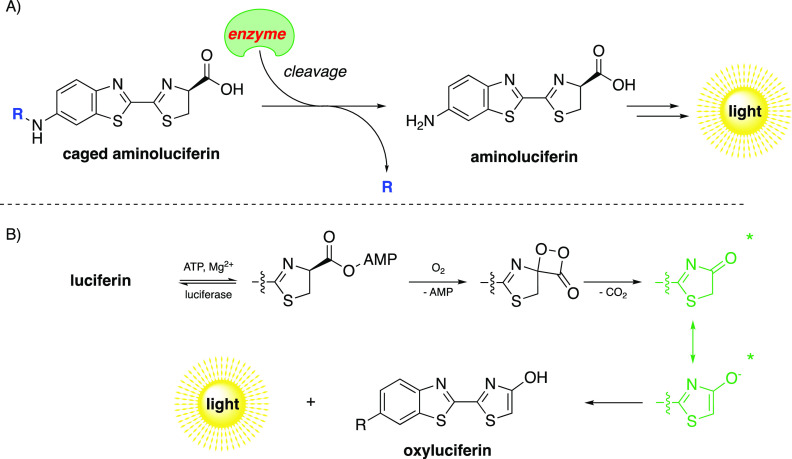
(A) General Reaction Scheme for an Aminoluciferin (aLuc)-Based Assay; upon Enzymatic Activity, d-Aminoluciferin Is Released from the Caged Substrate, Which Can Subsequently Be Converted into a Proportional Light Signal in the Presence of Luciferase, ATP, Mg^2+^, and O_2_ and (B) Luciferase-Catalyzed Luminescence Reaction Mechanism as Found in Fireflies

In recent decades, luminescent imaging has emerged as a powerful tool to monitor enzymatic activity. Luminescent imaging generally occurs via the enzymatic oxidation of a small molecule, without any external light source, and proceeds with a high signal-to-noise ratio as the background signal is generally lower as compared to fluorescence imaging.^1^ Numerous applications have been reported for luminescent imaging, including sensing reactive oxygen and nitrogen species, imaging of cancer cells, and quantifying in vivo glucose uptake.^2−4^ White and co-workers were among the first to report the use of d-luciferin derivative aminoluciferin (aLuc) in proteolytic assays in which the amino function is coupled to the C-terminus of a peptide.^5,6^ The free 6′-amino group of aLuc appeared crucial to preserve its luminescent properties (similar to the 6′-hydroxy in d-luciferin), while modification of the amine, such as coupling to a carboxylic acid to form an amide, usually results in quenched luminescence.^7^ These quenched luminescent probes have also been referred to as “caged” luciferins and can be used in luminescent enzymatic assays, as upon enzymatic activity the free aLuc is released which can readily be quantified (Scheme 1A).^7−9^ After proteolytic activity, the AMP adduct of luciferin is generated upon the action of ATP and Mg^2+^. The aLuc is subsequently oxidized to the excited oxyluciferin by the catalytic action of luciferase, and consecutive relaxation back into the ground state causes the release of photons. Both keto- and enol-intermediates are thought to be the actual light-generating intermediates in the enzyme pocket, the reaction mechanism found in fireflies is depicted in Scheme 1B.^1^ Numerous luminescent probes have been developed according to this uncaging principle and are widely used in combination with a large variety of enzymes among which hydrolases and proteases.^10−13^

**Figure 1:**
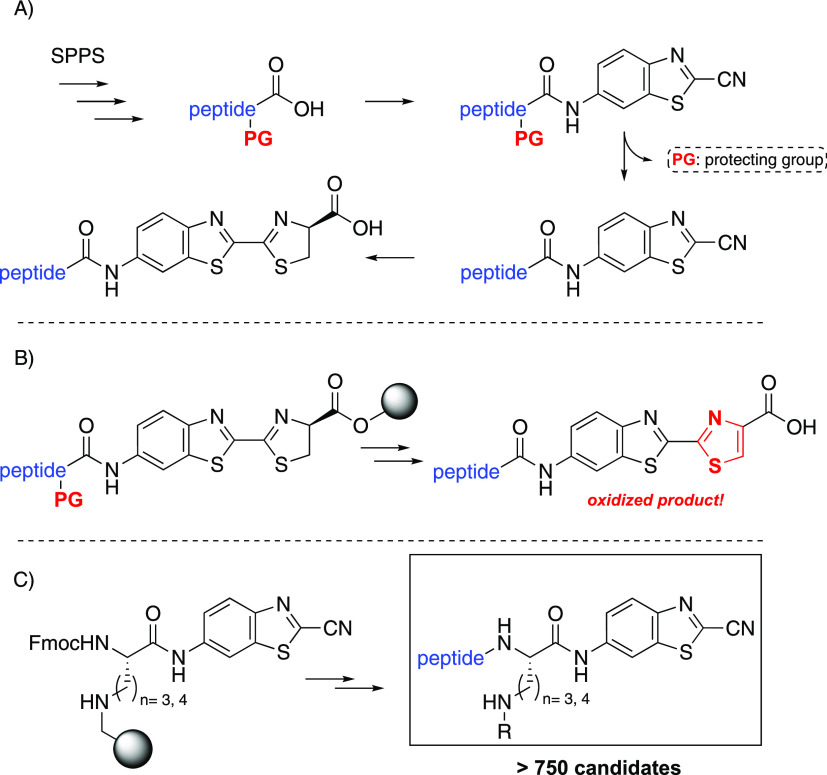
(A) Conventional solution-phase method for the synthesis of aminoluciferin peptides via C-terminal activation of a protected peptide and the subsequent side chain deprotection and d-cysteine condensation all in solution. (B) Hybrid SPPS method reported by Kovacs et al., loading of the aminoluciferin on a solid support afforded the dehydrogenated product. (C) This work with the side chain anchoring of the P_1_ amino acid residue on a solid support and the subsequent regular Fmoc-based SPPS chemistry.

In order to extend the applications of luminescence imaging, new caged luciferins are required. Current methods for the preparation of luminescent caged peptides mostly rely on the use of solution-phase synthesis. Generally, a protected peptide is C-terminally activated with reagents such as isobutyl chloroformate (IBCF) in the presence of a base, e.g., *N*-methylmorpholine (NMM), prior to the addition of the aLuc precursor 6-aminobenzo[*d*]thiazole-2-carbonitrile (6-ABTC, Figure 1A).^14−16^ After the coupling, the side chain protecting groups of the peptide can be cleaved off, and a final condensation reaction with d-cysteine yields the caged luciferin peptide. The use of solid-phase peptide synthesis (SPPS) to construct the caged luciferins would drastically lower the number of steps in solution-phase, eliminate multiple purification steps, and especially allow for a parallel automated workflow.^17^ Preliminary work by Kovacs et al. revealed that loading of the carboxylic acid moiety of aLuc onto a solid support and subsequent elongation of the peptide chain was unsuccessful, due to the inherent instability of the thiazoline moiety of aLuc (Figure 1B).^18^ The thiazoline ring is prone to oxidation resulting in the corresponding thiazole derivative, a well-known luciferase inhibitor.^19,20^

Herein, we present an SPPS method for the synthesis of caged luminescent peptides starting with the side chain anchoring of either a lysine or an ornithine P_1_ residue (Figure 1C). The ornithine moiety allows for on-resin guanidinylation into the corresponding arginine residue. The introduction of the aLuc moiety after resin cleavage renders our approach compatible with SPPS, especially since the relatively unreactive 6-ABTC moiety is introduced at the P_1_ C-terminus prior to the solid-phase reactions. We chose luminescent caged peptide substrates bearing an arginine or lysine residue on the P_1_ position, as the targeted trypsin-like serine proteases prefer positively charged P_1_ residues.^21,22^ With the SPPS method, we aim to accelerate the development of novel anticoagulation therapies and the design of new activity-based probes for various serine proteases.

## Results and Discussion

**Scheme 2:**
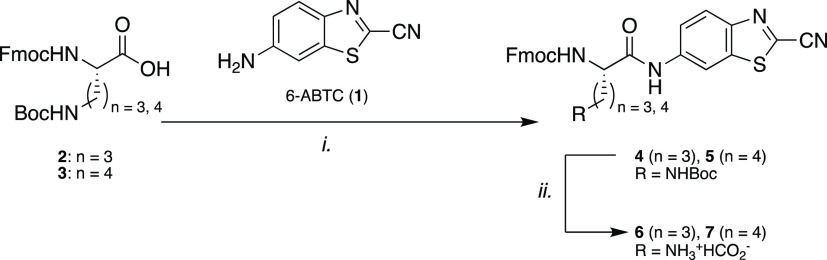
Synthesis of the Luminescent Precursor Amino Acids **6** and **7** (i) 6-ABTC (**1**, 1 equiv), PCl_3_ (0.51 equiv), pyr, 3.5 h, 40 °C, 77–98%; (ii) HCO_2_H, lyophilization, quant.

In order to develop a robust method for the synthesis of luminescent-labeled peptides via SPPS, the stable 6-ABTC (**1**) aLuc precursor was used throughout the solid-phase synthesis procedure. We were inspired by the side chain anchoring approaches toward caged 7-amino-4-methylcoumarin (AMC) fluorescent peptide probes by Beythien et al. and Hamzé et al., both using an arginine residue at P_1_.^23,24^ The synthesis of C-terminally modified peptides via P_1_ side chain anchoring has been addressed as demonstrated in numerous literature accounts.^25−28^ We chose to address the inherent unreactive character of the amino group of 6-ABTC (**1**) by coupling to the P_1_ residue in solution, prior to the solid-phase steps (Scheme 2). Thus, **1** was synthesized on a multigram scale starting from 2-chlorobenzo[*d*]thiazole according to a known literature procedure.^29^ Initial coupling reactions of either Fmoc-Orn(Boc)–OH (**2**) or Fmoc-Lys(Boc)–OH (**3**) with **1** under standard peptide coupling conditions (HOBt, DIC/HATU, DIPEA) or C-terminal activation with IBCF and NMM all appeared to be low yields with significant amounts of byproducts, presumably because the acid needs to be highly activated to allow aniline coupling. However, when switching to preactivation of **1** with phosphorus trichloride in pyridine, prior to the addition of **2** or **3**, high yields of amino acids **4** and **5** were obtained (Scheme 2).^30,31^ The Boc side chain protecting groups were subsequently removed with formic acid, and the residues were lyophilized to obtain the C-terminal functionalized ornithine and lysine building blocks **6** and **7**, respectively, with the free side chain amine available for further conjugation.

**Scheme 3:**
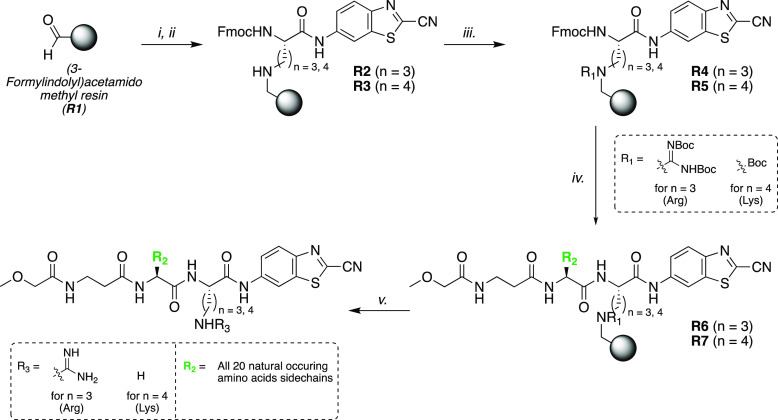
Validation Solid-Phase Peptide Synthesis Library of Luminescent Caged Peptides via Side Chain Anchoring of Amino Acids **6** or **7** via Reductive Amination with **R1** ((3-Formylindolyl)acetamido Methyl Resin) (i) **6** or **7** (2 equiv), THF/TMOF (1:1), 4 h, rt. (ii) NaBH_3_CN (2 equiv), AcOH (3.5 equiv), 2 h, rt. (iii) For *n* = 3: **8** (2 equiv), DIC (2 equiv), DCM, rt, 16 h. For *n* = 4: Boc_2_O (3 equiv), DIPEA (3 equiv), DMF, rt, 16 h. (iv) (a) 3% DBU in DMF, DMF (3×); (b) HOBt (3.6 equiv), DIC (3.3 equiv), Fmoc-AA–OH/Cap–OH (methoxy acetic acid) (3 equiv) (3×). (v) DMF. (v) TFA/DCM (1:1, v/v), 2 h

The building blocks **6** and **7** then had to be attached to an aldehyde-functionalized solid support via reductive amination.^32^ Since the nitrile moiety of **1** can be prone to (acidic) hydrolysis, we chose to use the highly acid labile (3-formylindolyl)acetamido methyl polystyrene resin (**R1**, 100–200 mesh).^23,24^ Reductive amination of either **6** or **7** with the aldehyde-functionalized resin was carried out with NaBH_3_CN in a 1:1 mixture of tetrahydrofuran and trimethyl orthoformate (TMOF) as a water scavenger for 4 h at room temperature (Scheme 3). This afforded the anchored ornithine and lysine resins **R2** and **R3**, respectively. Next, the secondary bound amines **R2** and **R3** were further functionalized before proceeding with N-terminal peptide elongation. The lysine derivatives (**R3**, *n* = 4) were Boc protected using di-*tert*-butyl dicarbonate and DIPEA in DMF while agitating overnight to afford **R5**. In order to obtain the arginine derivatives from the ornithine-bound resin, we aimed to guanidinylate the immobilized secondary amine. In our validation library, we employed two equivalents of *N*,*N*′-di-Boc-thiourea (**8**) in DCM overnight with *N*,*N*′-diisopropylcarbodiimide (DIC) as desulfurization reagent, which afforded resin **R4**.^33^ DIC was used instead of more commonly used HgCl_2_, which however is not compatible with SPPS due to the formation of insoluble HgS. Knowing that MeOCH_2_C(O)-β-Ala-Gly-Arg-aLuc is an excellent substrate for trypsin-like serine proteases such as thrombin, we chose to design our validation library accordingly by synthesizing tripeptides using P_2_ residues the naturally occurring amino acids in order to study the P_2_ dependence.^34,35^

To this extent, the peptide chains of the **R4** and **R5** resins were elongated via parallel Fmoc SPPS chemistry. The Fmoc deprotections were executed with 3% DBU in DMF, as these conditions in our experience gave considerably better results than the use of 20% piperidine in DMF. The subsequent coupling reactions with the Fmoc-protected amino acids and the N-terminal cap (3 equiv) were conducted using standard coupling conditions (3.6 equiv of HOBt and 3.3 equiv of DIC in DMF). This afforded the functionalized resins **R6** and **R7**, respectively. The peptides were then cleaved from the resin with trifluoroacetic acid (TFA) in DCM (1:1, v/v, addition of 2.5% TIS and EDT for Trt-containing sequences), lyophilized, and purified with RP-HPLC in order to obtain pure 6-ABTC-conjugated peptides, which were all analyzed by ESI–MS and NMR (typical scale of 100 mg unfunctionalized resin per substrate gave 2–5 mg of purified product, corresponding to 5–12% isolated yield). Thus, a tripeptide library consisting of 40 peptides MeOCH_2_C(O)-β-Ala-**P**_**2**_-Arg/Lys-aLuc was obtained, with **P**_**2**_ being any of the 20 naturally occurring amino acids (Scheme 3).

**Scheme 4:**
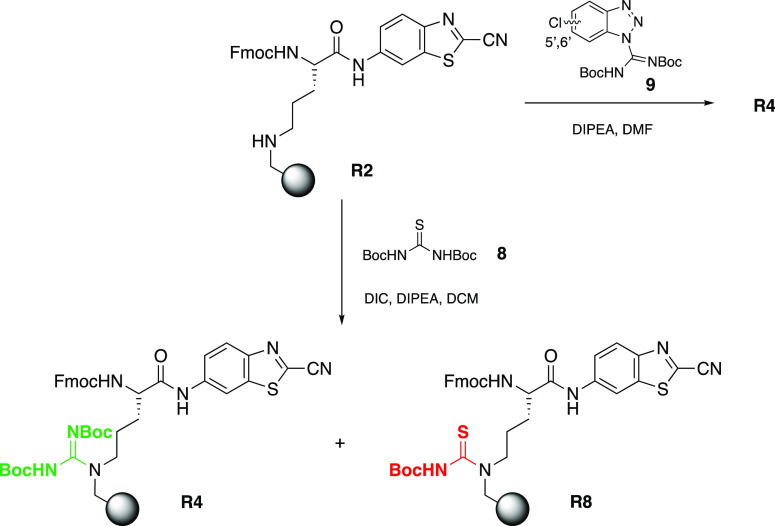
Conversion of Resin-Bound Secondary Amine **R2** into the Di-Boc-Protected Guanidine **R4** and the Observed Thiourea Byproduct **R8** When Using *N*,*N*′-Di-Boc-thiourea (**8**) and DIC Activation The use of **9** solely afforded the desired resin-bound product **R4**.

However, we often obtained a significant amount of thiourea byproduct **R8** when using the *N*,*N*′-di-Boc-thiourea (**8**) method instead of the desired protected resin-bound guanidine **R4** (Scheme 4). Initial attempts to shorten the guanidinylation reaction time or add fresh reagents did not overcome the formation of the byproduct **R8**. Neither the use of different activators such as EDC, copper(I), or copper(II) chloride lowered the formation of thiourea **R8**.^36,37^ Different guanidinylation reagents such as *N*,*N*′-di-Boc-*S*-methylisothiourea, 1-[*N*,*N*′-(di-Boc)amidino]pyrazole, Goodman’s reagent or cyanuric chloride (TCT) were also evaluated but gave rise to an even lower level of guanidinylation as analyzed by LCMS analysis directly after resin cleavage (data not shown).^38−40^ Eventually, we tested the benzotriazole-based reagent **9**, as described by Moroder and Musiol in 2001.^41^ This reagent could after optimization easily be synthesized in our hands-on multigram scale (as a mixture of the 5′ and 6′-isomers) without the use of toxic HgCl_2_ which was originally used by the authors. Reagent **9** revealed complete conversion of **R2** into **R4** and fewer byproducts (in particular **R8**) were observed compared to the method with reagent **8** and DIC activation.

**Scheme 5:**
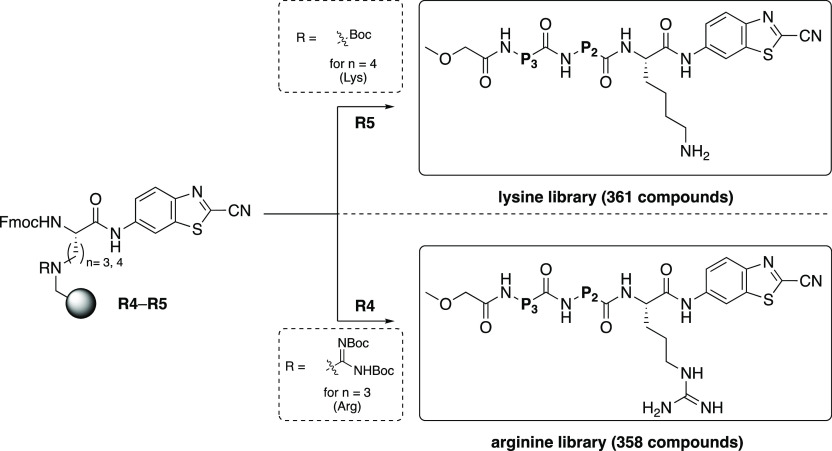
General Structures of the Two Large Tripeptide Libraries That Were Synthesized in This Work Starting from the Key Intermediate Resins **R4** and **R5** P_3_ = natural amino acid, P_2_ = natural amino acid and P_1_ = either arginine (*n* = 3) or lysine (*n* = 4). After regular Fmoc SPPS chemistry and subsequent RP-HPLC purification after cleavage, a total amount of 719 peptides was obtained.

With the validation library in hand and having optimized the guanidinylation reaction, we continued to assess our method for the synthesis of two larger peptide libraries as proof of our synthetic principle. We aimed to validate our newly developed SPPS method by parallel synthesizing two separate tripeptide libraries with alteration of the P_2_ and P_3_ residues for all-natural available amino acids. Multiple batches of ornithine- and lysine-functionalized building blocks **6** and **7** were synthesized and coupled to the (3-formylindolyl)acetamidomethyl resin (**R1**) via reductive amination to afford **R2** and **R3**. After the reductive amination, the lysine-anchored resin was again temporarily protected with a Boc group, and the ornithine resin was guanidinylated with the benzotriazole-based reagent **9** to afford the key intermediate arginine- or lysine-anchored resins **R4** and **R5**, respectively. The peptide chains were subsequently elongated similar to those for the validation libraries via regular Fmoc chemistry and with the N-termini capped (see the Supporting Information). The cleaved products were purified with RP-HPLC to obtain two libraries with all available natural amino acids (except cysteine) at P_2_ and P_3_ thereby providing ultimate proof of this synthetic concept (see: Scheme 5). A total of 719 substrates were obtained via this route, which were all analyzed by LCMS (lysine library average purity: 83%, arginine library: 63%). The purity of the arginine substrate library was generally slightly lower due to the smaller amount of material available (0.03 mmol per substrate) to synthesize all of the target substrates, which sometimes made the purification more challenging as compared to the lysine substrates (0.05 mmol per substrate). Additionally, for the lysine library, it appeared that the RP-HPLC purification was most successful by using a trifluoroacetic acid (TFA)-acidified eluent instead of formic acid (0.1%). The lysine products that were initially purified with the formic acid method revealed some extent of oligomerization after several days, which was not observed for the TFA salts of the purified products. Presumably, due to the weaker acid (p*K*_a_ HCO_2_H = 3.7 as compared to p*K*_a_ TFA = 0.2), the lower extent of protonation of the amine causes the lysine side chain to react with cyanide groups thereby leading to oligomers or due to the volatility of the counterion.

Using this approach, we obtained two large tripeptide libraries. These large numbers of compounds directly demonstrate the scalability and applicability of our novel parallel SPPS synthesis methodology and prove that our unique method is highly suitable for automated workflow.

In our experience, the 6-ABTC-functionalized peptides appeared to be significantly more stable than the corresponding aLuc peptides. Hence, we reasoned that the active aLuc substrates could be generated from the nitrile precursors with the selective d-cysteine condensation reaction in the final stage. Since the reaction of d-cysteine and 6-ABTC has extensively been described in the literature and rapidly yields the aLuc functionalized derivative, we hypothesized this reaction could be performed in situ.^42−45^

**Scheme 6:**
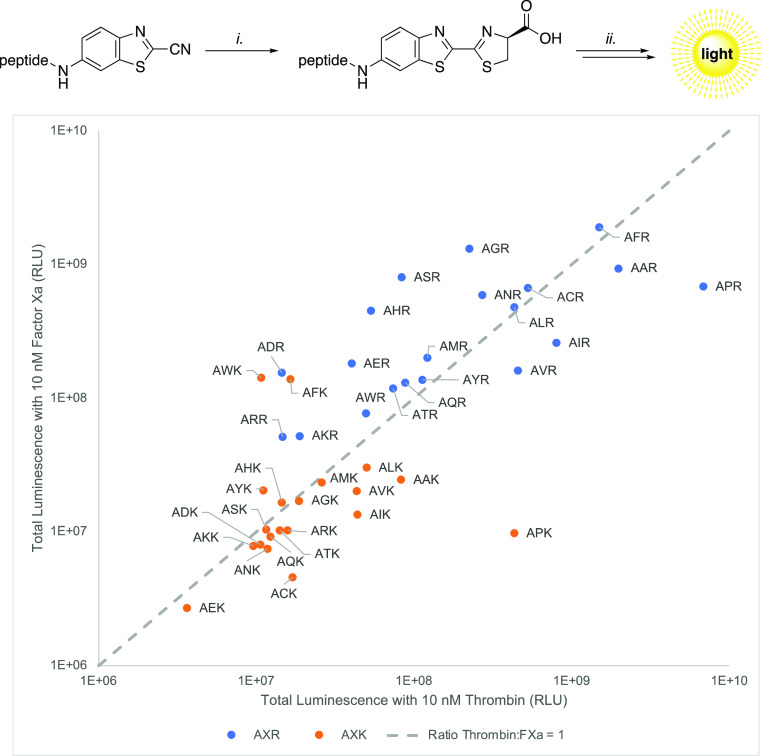
Total Luminescence of Substrate Candidates (β-AXR/β-AXK) Using Thrombin and Factor Xa (i) β-AXR/β-AXK substrate (1 equiv) d-cysteine (1.5 equiv), buffer (25 mM HEPES, 125 mM NaCl, 0.5% BSA, pH = 7.4), 37 °C, 30 min. (ii) Protease (thrombin/FXa, final conc 10 nM), ATP (1.5 equiv), MgCl_2_ (10 equiv), Quantilum recombinant firefly luciferase (final conc 10 μM). Screening of peptide library with the substrates (667 μM) methoxyacetyl-βAXR-6ABTC (in blue) and methoxyacetyl-βAXK-6ABTC (in orange) after the condensation reaction with d-cysteine. The proteolytic activity of the substrates towards both factor Xa and thrombin were determined and their ratio was plotted (ratio 1:1 *y* = *x*) against the total luminescence for either thrombin or FXa (*x*- and *y*-axis, respectively).

To provide a preliminary example, we ran our validation peptide library (40 substrates) against the two trypsin-like serine proteases Factor Xa (FXa) and thrombin (FXIIa) in order to demonstrate the protease profiling potential. First, we incubated the 6-ABTC-conjugated peptides with d-cysteine for 30 min at 37 °C in a buffer in order to generate the required aLuc-caged substrates, followed by the addition of protease, ATP, MgCl_2,_ and luciferase. Hereafter, the luminescence was recorded at 37 °C and the area under the curve (AUC) was calculated (see: Supporting Information). The ratio of substrate hydrolysis toward either thrombin or FXa was determined and is depicted in Scheme 6. The results in Scheme 6 demonstrate that both the synthetic approach and the in situ d-cysteine condensation are robust.

These preliminary data reveal that incorporation of proline at P_2_ in both libraries gave a significant increase in selectivity toward thrombin, which is consistent with known substrates encountered in the literature.^46^ Ultimately, hits obtained from our libraries may be further optimized for use in diagnostic applications in order to assess thrombin and FXa activity with a luminescent readout. This newly developed synthesis method allows for rapid luminescent profiling of substrate specificity of serine proteases and thereby contributes to the development of novel anticoagulation therapies or to the design of new specific activity-based probes for point-of-care applications. The use of the 6-ABTC-functionalized peptides and the subsequent d-cysteine chemistry appears to be a novel technique in order to generate the active aLuc substrates in the final stage, facilitating conventional peptide synthesis prior to the proteolytic assays. The increased stability of the 6-ABTC peptides allows for convenient storage, as the aLuc moiety is prone to oxidation over time and can now be generated in situ, making our method suitable for SPPS.

The results presented in this work clearly demonstrate the synthetic scope, scalability, and feasibility of our novel parallel SPPS method and its potential for profiling serine proteases with a luminescent readout. Currently, we are screening various proteases in combination with the compound libraries to hopefully find optimal enzyme–substrate pairs for potential diagnostic purposes. Potential hits will be resynthesized, validated, and optimized for the desired activity-based assay.

## Conclusions

In short, we developed a suitable SPPS method for the rapid parallel synthesis of C-terminally 6-ABTC modified peptides via side chain anchoring of either a lysine or ornithine P_1_ residue. The use of 6-ABTC and the late-stage in situ condensation reaction with d-cysteine were key steps in rendering our approach suitable for solid-phase synthesis. The ornithine P_1_ residues were converted into the corresponding arginine residues via an on-resin guanidinylation reaction, which was optimized for a larger scale. As proof of our synthetic methodology, we synthesized three peptide libraries containing >750 tripeptide compounds. This method represents a major step forward in the ease of synthesizing luminescent peptide libraries, as most frequently these substrates are currently being made using regular solution-phase chemistry via C-terminal activation. We showed that both the synthetic approach and the in situ d-cysteine condensation are robust in order to profile serine proteases, as demonstrated with our validation library screening. We feel that our method can strongly contribute to the synthesis of luminescent caged peptide substrates via a parallel and automated workflow, as we primarily focused on the synthetic aspects and practicalities in this work. The compounds from these libraries are currently being screened in our laboratories for potential use in diagnostic protease assays to ultimately find optimal enzyme–substrate pairs for diagnostic and/or therapeutic applications.

## Experimental Procedures

### Synthesis

#### (9*H*-Fluoren-9-yl)methyl *tert*-Butyl (5-((2-Cyanobenzo[*d*]thiazol-6-yl)amino)-5-oxopentane-1,4-diyl)(*S*)-dicarbamate (Fmoc-Orn(Boc)-6ABTC, **4**)

Was prepared in multiple batches. Typical procedure: 6-aminobenzo[*d*]thiazole-2-carbonitrile (**1**, 300 mg, 1.71 mmol) was dissolved in dry pyridine (6 mL) in a flame-dried flask. PCl_3_ (78 μL, 890 μmol) was added dropwise and the reaction mixture was stirred for 1.5 h. Fmoc-Orn(Boc)–OH (778 mg, 1.71 mmol) was added to dry pyridine (3 mL) and the reaction mixture was stirred for 3 h at 40 °C. The reaction mixture was allowed to cool down to rt, diluted with EtOAc (150 mL), and washed with 10% aqueous citric acid (150 mL). The aqueous phase was re-extracted with EtOAc (75 mL). The combined organic layers were washed with 10% aqueous citric acid (100 mL), saturated aqueous NH_4_Cl (2 × 100 mL), and brine (100 mL). The combined organic layers were dried with MgSO_4_, concentrated in vacuo, and purified with silica gel column chromatography (20 → 80% EtOAc/heptane) to afford Fmoc-Orn(Boc)-6ABTC (**4**, 1.03 g, 98%) as a yellow solid. **TLC** (EtOAc/heptane, 1:1 v/v) *R*_F_ = 0.69. ^1^H NMR (500 MHz, CDCl_3_): δ 9.26 (s, 1H), 8.66 (d, *J* = 2.1 Hz, 1H), 8.09 (d, *J* = 8.9 Hz, 1H), 7.76 (d, *J* = 7.6 Hz, 2H), 7.60 (t, *J* = 7.2 Hz, 2H), 7.57–7.53 (m, 1H), 7.39 (t, *J* = 7.5 Hz, 2H), 7.30 (t, *J* = 5.5 Hz, 2H), 5.76–5.71 (m, 1H), 4.87–4.83 (m, 1H), 4.68–4.64 (m, 1H), 4.42 (d, *J* = 7.1 Hz, 2H), 4.22 (t, *J* = 7.0 Hz, 1H), 3.63–3.50 (m, 1H), 3.16–3.06 (m, 1H), 2.04 (s, 2H), 1.67 (s, 2H), 1.45 (s, 9H). ^**13**^**C NMR** (126 MHz, CDCl_3_): δ 171.3, 157.6, 148.7, 143.7, 141.4, 136.9, 127.9, 127.2, 125.3, 125.2, 121.0, 120.1, 120.1, 113.2, 111.5, 47.3, 28.5.

#### (9*H*-Fluoren-9-yl)methyl (*S*)-(5-Amino-1-((2-cyanobenzo[*d*]thiazol-6-yl)amino)-1-oxopentan-2-yl)carbamate (Fmoc-Orn-6ABTC, **6**)

Was prepared in multiple batches. Typical procedure: Fmoc-Orn(Boc)-6ABTC (**4**, 785, 1.28 mmol) was dissolved in HCO_2_H (10 mL) and stirred overnight at rt. The reaction mixture was concentrated in vacuo and the product was lyophilized overnight to afford the HCO_2_H salt of Fmoc-Orn-6ABTC (**6**, 733 mg, quant) as a yellow solid. ^**1**^**H NMR** (400 MHz, CD_3_OD): δ 8.64 (d, *J* = 2.1 Hz, 1H), 8.12 (d, *J* = 9.0 Hz, 1H), 7.78 (d, *J* = 7.6 Hz, 2H), 7.70 (dd, *J* = 9.0, 2.1 Hz, 1H), 7.66 (t, *J* = 7.5 Hz, 2H), 7.37 (t, *J* = 7.5 Hz, 2H), 7.33–7.24 (m, 2H), 4.42 (qd, *J* = 10.6, 6.7 Hz, 2H), 4.35–4.31 (m, 1H), 4.21 (t, *J* = 6.7 Hz, 1H), 2.97 (t, *J* = 7.3 Hz, 2H), 1.88–1.73 (m, 3H). ^**13**^**C NMR** (101 MHz, CD_3_OD): δ 177.3, 172.9, 169.9, 158.3, 149.9, 145.1, 142.2, 140.4, 138.0, 136.8, 128.8, 128.1, 125.9, 122.3, 120.9, 114.0, 112.9, 67.9, 56.5, 39.7, 30.1, 24.2, 22.1. **HRMS** (*m*/*z*): [M + H]^+^ calcd for C_28_H_25_N_5_O_3_S, 512.1756; found, 512.1752.

#### (9*H*-Fluoren-9-yl)methyl *tert*-Butyl (6-((2-Cyanobenzo[*d*]thiazol-6-yl)amino)-6-oxohexane-1,5-diyl)(*S*)-dicarbamate (Fmoc-Lys(Boc)-6ABTC, **5**)

Was prepared in multiple batches. Typical procedure: 6-aminobenzo[*d*]thiazole-2-carbonitrile (**1**, 501 mg, 2.86 mmol) was dissolved in dry pyridine (10 mL) in a flame-dried flask. PCl_3_ (130 μL, 1.49 μmol) was added dropwise, and the reaction mixture was stirred for 1.5 h. Fmoc-Lys(Boc)–OH (1340 mg, 2.86 mmol) was added to dry pyridine (5 mL) and the reaction mixture was stirred for 3 h at 40 °C. The reaction mixture was allowed to cool down to rt, diluted with EtOAc (150 mL), and washed with 10% aqueous citric acid (150 mL). The aqueous phase was re-extracted with EtOAc (75 mL). The combined organic layers were washed with 10% aqueous citric acid (100 mL), saturated aqueous NH_4_Cl (2 × 100 mL), and brine (100 mL). The combined organic layers were dried with MgSO_4_, concentrated in vacuo, and purified with silica gel column chromatography (0 → 70% EtOAc/heptane) to afford Fmoc-Lys(Boc)-6ABTC (**5**, 1.38 g, 77%) as a yellow solid. **TLC** (EtOAc/heptane, 9:1 v/v) *R*_F_ = 0.83. ^**1**^**H NMR** (500 MHz, CD_3_OD): δ 8.65–8.60 (m, 1H), 8.08 (dd, *J* = 9.2, 4.3 Hz, 1H), 7.79–7.74 (m, 2H), 7.69–7.63 (m, 3H), 7.39–7.34 (m, 2H), 7.32–7.25 (m, 2H), 4.40–4.36 (m, 2H), 4.28–4.24 (m, 1H), 4.22–4.18 (m, 1H), 3.07–3.00 (m, 2H), 1.89–1.82 (m, 1H), 1.80–1.71 (m, 1H), 1.55–1.47 (m, 4H), 1.39 (s, 9H). ^**13**^**C NMR** (126 MHz, CDCl_3_): δ 173.7, 158.6, 149.8, 145.2, 145.1, 142.5, 140.5, 138.0, 136.7, 128.7, 128.1, 126.2, 125.9, 122.2, 120.9, 114.0, 112.8, 79.8, 67.9, 57.2, 48.4, 38.4, 32.9, 30.6, 28.7, 24.2.

#### (9*H*-Fluoren-9-yl)methyl (*S*)-(6-Amino-1-((2-cyanobenzo[*d*]thiazol-6-yl)amino)-1-oxohexan-2-yl)carbamate (Fmoc-Lys-6ABTC, **7**)

Was prepared in multiple batches. Typical procedure: Fmoc-Lys(Boc)-6ABTC (**5**, 1.38 g, 2.21 mmol) was dissolved in HCO_2_H (10 mL) and stirred overnight at rt. The reaction mixture was concentrated in vacuo and the product was lyophilized overnight to afford the HCO_2_H salt of Fmoc-Lys-6ABTC (**7**, 1260 mg, quant) as a yellow solid. ^**1**^**H NMR** (400 MHz, CD_3_OD): δ 8.61 (d, *J* = 2.1 Hz, 1H), 8.07 (d, *J* = 9.0 Hz, 1H), 8.28 (s, 2H), 7.75 (d, *J* = 7.5 Hz, 2H), 7.70–7.60 (m, 3H), 7.35 (t, *J* = 7.5 Hz, 2H), 7.26 (t, *J* = 7.5 Hz, 2H), 4.40 (dd, *J* = 6.7, 3.4 Hz, 2H), 4.28 (dd, *J* = 8.9, 5.3 Hz, 1H), 4.19 (t, *J* = 6.7 Hz, 1H), 2.93–2.88 (m, 2H), 1.94–1.83 (m, 1H), 1.83–1.62 (m, 3H), 1.58–1.40 (m, 2H). ^**13**^**C NMR** (101 MHz, CD_3_OD): δ 172.0, 157.2, 148.5, 143.7, 141.2, 139.1, 136.6, 135.4, 127.4, 126.7, 124.8, 124.6, 120.9, 119.6, 112.7, 111.6, 66.5, 55.6, 47.0, 39.1, 31.3, 26.8, 22.5. **HRMS** (*m*/*z*): [M + H]^+^ calcd C_29_H_27_N_5_O_3_S, 526.1912; found, 526.1909.

#### *N*,*N*′-Di-*tert*-butoxycarbonyl-5-chloro-1*H*-benzotriazole-1-carboxamidine and *N*,*N*′-Di-*tert*-butoxycarbonyl-6-chloro-1*H*-benzotriazole-1-carboxamidine (**9**)^41,47^

It was prepared in multiple batches. Typical procedure: *N*,*N*′-di-Boc-thiourea (7.7 g, 27.9 mmol) and 5-chlorobenzotriazole (4.3 g, 27.9 mmol) were dissolved in anhydrous MeCN (250 mL) and DIPEA (14.6 mL, 83.6 mmol) was added. The reaction mixture was cooled to 0 °C, EDCl (10.7 g, 55.7 mmol) was added, and the reaction was stirred for 18 h at rt. The mixture was diluted with EtOAc (600 mL) and washed with 10% aqueous citric acid (300 mL) and brine (300 mL). The organic layer was dried with MgSO_4_, concentrated in vacuo, and purified through silica gel column chromatography (0 → 15% EtOAc/heptane) to afford the product as a mixture of the 5′- and 6′ isomers (**9**, 3.49 g, 32%) as a white solid. **TLC** (EtOAc/heptane, 1:4 v/v) *R*_F_ = 0.32 (6′ isomer), 0.27 (5′ isomer). ^**1**^**H NMR** (500 MHz, CDCl_3_): δ 8.99 (s, 2H), 8.39 (s, 1H), 8.32 (d, *J* = 8.8 Hz, 1H), 8.09 (d, *J* = 1.9 Hz, 1H), 8.03 (d, *J* = 8.8 Hz, 1H), 7.63–7.58 (m, 1H), 7.51–7.45 (m, 1H), 1.52 (d, *J* = 17.5 Hz, 27H). ^**13**^**C NMR** (126 MHz, CDCl_3_): δ 131.1, 127.4, 121.1, 119.8, 116.3, 115.2, 28.1. **HRMS** (*m*/*z*): [M + Na]^+^ calcd for C_17_H_22_ClN_5_O_4_Na, 418.1258; found, 418.1244.

### Resin Loading Procedures

#### Loading of Fmoc-Orn-6ABCT (**2**): Typical Scale 10 g of Resin

(3-Formylindolyl)acetamidomethyl resin (10.00 g, 7.50 mmol according to loading) was suspended in dry THF/TMOF (1:1, 150 mL) in a flame-dried flask. Fmoc-Orn-6ABTC (**6**, 3.46 g, 6.75 mmol) was added, and the suspension was agitated for 4 h at rt on a rotary evaporator. NaBH_3_CN (848 mg, 13.5 mmol) in THF (5 mL) and AcOH (1.5 mL, 26.2 mmol) were added, and the suspension was agitated for 2 h. The resin was washed with DMF (3×) and DCM (3×) before the addition of **9** (2.82 g, 6.75 mmol) and DIPEA (3.26 mL, 18.7 mmol) in DMF (100 mL) and agitated for 18 h. Hereafter, the resin was washed with DMF (3×), DCM (3×), and Et_2_O (3×). The resin was dried under high vacuum to afford **R4** which was used in the next steps using regular Fmoc SPPS chemistry (see the Supporting Information). Multiple batches were prepared with a final loading between 0.20 and 0.22 mmol/g (determined by Fmoc quantification at 301 nm).

#### Loading of Fmoc-Lys-6ABCT (**3**): Typical Scale 10 g of Resin

(3-Formylindolyl)acetamidomethyl resin (10.00 g, 7.50 mmol according to loading) was suspended in dry THF/TMOF (1:1, 150 mL) in a flame-dried flask. Fmoc-Lys-6ABTC (**7**, 3.55 g, 6.75 mmol) was added, and the suspension was agitated for 4 h at rt on a rotary evaporator. NaBH_3_CN (848 mg, 13.5 mmol) in THF (5 mL) and AcOH (1.5 mL, 26.2 mmol) were added and the suspension was agitated for 2 h. The resin was washed with DMF (3×) and DCM (3×) before the addition of Boc_2_O (4.91 g, 22.5 mmol) and DIPEA (3.92 mL, 22.5 mmol) in DMF (100 mL), after which the mixture was agitated for 18 h. Hereafter, the resin was washed with DMF (3×), DCM (3×), and Et_2_O (3×). The resin was dried under high vacuum to afford **R5** which was used in the next steps with regular Fmoc SPPS chemistry (see the Supporting Information). Multiple batches were prepared with a final loading between 0.16–0.25 mmol/g (determined by Fmoc quantification at 301 nm).

## Supporting Information Available

Material and methods, experimental procedures, and NMR spectra (PDF)
