# Supplementary material for: Solid-Phase Synthesis of Caged Luminescent Peptides via Side Chain Anchoring
Source: Bioconjug Chem. 2023 Dec 6;34(12):2234–42. doi: 10.1021/acs.bioconjchem.3c00381 (PMC10739589; doi:10.1021/acs.bioconjchem.3c00381)
Supplement: Supplementary file 1 — bc3c00381_si_001.pdf [file bc3c00381_si_001.pdf]

Supporting Information

Solid-Phase Synthesis of Caged Luminescent Peptides via Sidechain Anchoring

Daan Sondag,[a] Jurriaan J. A. Heming,[a] Dennis W. P. M. Löwik,[a] Elena Krivosheeva,[b] Denise Lejeune,[a,b] Mark van Geffen,[b] Cornelis van 't Veer,[b] Waander L. van Heerde,[b,c] Marjolijn C. J. Beens,[d] Brian H. M. Kuijpers,[d] Thomas J. Boltje[a] and Floris P. J. T. Rutjes*,[a]

[a]*Institute for Molecules and Materials, Radboud University, 6525 AJ Nijmegen, The Netherlands*

[b]*Enzyre BV, Novio Tech Campus, Transistorweg 5-i, 6534 AT Nijmegen, The Netherlands*

[c]*Department of Haematology, Radboud University Medical Centre, Nijmegen, Geert Grooteplein Zuid 10, 6525 GA Nijmegen The Netherlands; Haemophilia Treatment Centre, Nijmegen Eindhoven Maastricht (HTC-NEM), The Netherlands*

[d]*Symeres BV, Kerkenbos 1013,6546 BB Nijmegen, The Netherlands*

**Corresponding author: floris.rutjes@ru.nl*

Table of Contents

1. Material and Methods **S2**
    a. General **S2**
2. Building block synthesis **S4–S9**
    a. 6-ABTC **S4**
    b. Fmoc-Orn-6ABTC **S6**
    c. Fmoc-Lys-6ABTC **S8**
    d. Guanidinylation reagent **S9**
3. Synthesis of methoxyacetyl-βAXR-6ABCT peptide conjugates (validation library) **S10–S21**
4. Synthesis of methoxyacetyl-βA**X**K-6ABCT peptide conjugates (validation library) **S22–S33**

5. Synthesis of methoxyacetyl-**XX**R-6ABCT library **S34–S45**

6. Synthesis of methoxyacetyl-**XX**K-6ABCT library **S46–S57**

7. Luminescent assay validation library **S58**

8. NMR spectra **S59–S106**

9. References **S107**

1. Material and Methods

a. General

NMR spectra were recorded on a Bruker Avance III 400 MHz or a Bruker 500 MHz spectrometer and the compounds were assigned using ^{1}H NMR, ^{13}C NMR, COSY, HSQCED and HMBC spectra. Chemical shifts were reported in parts per million (ppm.) relative to reference ($CDCl_3$: ^{1}H: 7.26 ppm. and ^{13}C 77.16 ppm.; CD_3OD: ^{1}H: 3.31 ppm. and ^{13}C 49.00 ppm.; D_2O: ^{1}H: 4.79 ppm.; $(CD_3)_2SO$: ^{1}H: 2.50 ppm. and ^{13}C 39.52 ppm.). NMR data are presented in the following way: chemical shift, multiplicity (s = singlet, bs = broad singlet, d = doublet, t = triplet, dd = doublet of doublets, ddd = doublet of doublet of doublets, dtd = doublet of triplet of doublets h = heptet, m = multiplet and/or multiple resonances) and coupling constants *J* in Hz. Peptides were synthesized using Fmoc solid-phase peptide (SPPS) chemistry and on resin coupling and deprotection steps were monitored using Kaiser tests. The (3-formylindolyl)acetamidomethyl resin (Novabiochem) was obtained from Sigma Aldrich (8.55098) the polymer matrix is copoly (styrene-1% DVB), 100 - 200 mesh. The two larger libraries were synthesized from the resins **R4** and **R5** via regular Fmoc SPPS at Symeres (Nijmegen, the Netherlands) to obtain two final libraries (P_1= arginine and P_1 = lysine) which were RP-HPLC purified and their purity was determined (LCMS). For the SPPS reactions peptide grade DMF was used. Mass spectra were recorded on a JEOL AccuTOF CS JMS-T100CS (ESI) mass spectrometer. Automatic flash column chromatography was executed on a Biotage Isolera Spektra One using SNAP or Silicycle cartridges (Biotage, 30–100 µm, 60Å) 4–50 g. Reactions were monitored using TLC F_{254} (Merck KGaA) by UV absorption detection (λ = 254 nm) and by spraying them with either 5% conc. H_2SO_4 in MeOH stain, cerium ammonium molybdate (Hannesian's) stain or with potassium permanganate stain followed by heating with a heat gun. Preparative HPLC was performed on a Phenomenex® Gemini-NX 3u C18 110A reversed-phase column (150 x 21.2 mm) using gradient elution with a constant flow

of 10 mL/min at 30 °C. MiliQ (0.1% TFA) and CH_3CN (0.1% TFA) were used as the solvents. The pure fractions containing product were combined and lyophilized overnight to yield the target compounds. Reactions under protective atmosphere were performed under positive Ar./N_2 flow using flame-dried glassware.

2. Building block synthesis

a. 6-ABTC

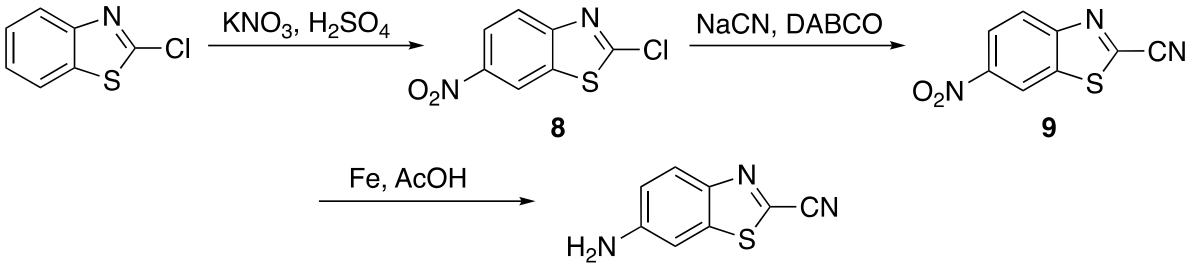

6-ABTC (**1**)

Scheme S1: Synthesis of 6-ABTC (**1**) from the commercially available 2-chlorobenzo[*d*]thiazole.[1]

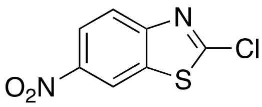

2-chloro-6-nitrobenzo[*d*]thiazole (8): 2-Chlorobenzo[*d*]thiazole (15.00 g, 88.43 mmol) was added portion wise to concentrated H_2SO_4 (90 mL) on an ice bath. KNO_3 (9.834 g, 97.27 mmol) was added portion wise and the reaction was stirred at 0 °C for 30 min. The reaction mixture was allowed to warm up to rt and stirred for 18 h at this temperature. The mixture was poured on ice water (300 mL) and the formed precipitate was collected by filtration. The crude product was rinsed with ice-cold water and saturated aqueous $NaHCO_3$ until acid free. The product was dried under high vacuum overnight and recrystallized from EtOH (650 mL) to afford **8** (16.81 g, 89%) as an off-white solid. **TLC** (EtOAc/heptane, 1:4 v/v) R_F = 0.60. **^{1}H NMR** (500 MHz, $CDCl_3$) δ 8.75 (d, *J* = 2.3 Hz, 1H), 8.38 (dd, *J* = 9.0, 2.3 Hz, 1H), 8.07 (d, *J* = 9.0 Hz, 1H). **^{13}C NMR** (126 MHz, $CDCl_3$) δ 158.9, 154.9, 136.6, 123.4, 122.3, 117.8.

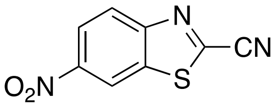

6-nitrobenzo[*d*]thiazole-2-carbonitrile (9): 2-Chloro-6-nitrobenzo[*d*]-thiazole (**8**, 10.78 g, 50.23 mmol) was dissolved in MeCN (1000 mL) and DABCO (845.2 mg, 7.53 mmol) was added. NaCN (2.78 g, 56.83 mmol) was dissolved in water (100 mL) and added dropwise to the stirred reaction mixture. After 24 h the reaction mixture was quenched with aqueous iron(III) chloride hexahydrate (0.3 M, 50 mL) and diluted with water (350 mL). The reaction mixture was extracted with EtOAc (3 × 400 mL) and the combined organic layers were washed with brine (100 mL), dried with $MgSO_4$ and concentrated *in vacuo*. The crude product

was loaded on a silica plug and flushed with $CHCl_3$ (2000 mL), concentrated and dried under high vacuum to afford **9** (8.30 g, 81%) as a yellow solid. **TLC** ($CHCl_3$) R_F = 0.33. **^{1}H NMR** (500 MHz, $CDCl_3$) δ 8.95 (d, *J* = 2.2 Hz, 1H), 8.52 (dd, *J* = 9.1, 2.2 Hz, 1H), 8.38 (d, *J* = 9.1 Hz, 1H). **^{13}C NMR** (126 MHz, $CDCl_3$) δ 155.4, 147.4, 141.9, 135.7, 126.2, 123.2, 118.6, 112.1.

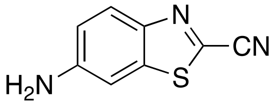

6-aminobenzo[*d*]thiazole-2-carbonitrile (1): 6-Nitrobenzo[*d*]-thiazole-2-carbonitrile (**9**, 7.70 g, 37.53 mmol) was suspended in AcOH (700 mL). Iron dust (104.8 g, 1.88 mmol) was added and the reaction was stirred for 24 h. The reaction was diluted with water (1400 mL) and filtered over Celite. The aqueous solution was extracted with EtOAc (3 × 700 mL) and washed with brine (400 mL). The crude product was loaded on a silica plug and flushed with $CHCl_3$ (2000 mL), concentrated and dried under high vacuum to afford **1** (3.45 g, 53%) as a yellow solid. **TLC** (DCM) R_F = 0.26. **^{1}H NMR** (500 MHz, $CDCl_3$) δ 7.95 (d, *J* = 8.9 Hz, 1H), 7.08 (d, *J* = 2.2 Hz, 1H), 6.95 (dd, *J* = 8.9, 2.3 Hz, 1H). **^{13}C NMR** (126 MHz, $CDCl_3$) δ 147.6, 145.5, 138.0, 131.0, 125.9, 117.6, 103.8, 77.2. **HRMS** (m/z): $[M + H]^+$ calcd. for $C_8H_5N_3S$: 176.0282, found 176.0294.

b. Fmoc-Orn-6ABTC[2, 3]

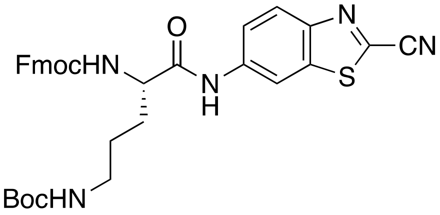

(9*H*-Fluoren-9-yl)methyl *tert*-butyl (5-((2-cyanobenzo[*d*]thiazol-6-yl)amino)-5-oxopentane-1,4-diyl)(*S*)-dicarbamate (Fmoc-Orn(Boc)-6ABTC, 4): 6-Aminobenzo[*d*]thiazole-2-carbonitrile (**1**, 300 mg, 1.71 mmol) was dissolved in dry pyridine (6 mL) in a flame-dried flask. PCl_3 (78 µL, 890 µmol) was added dropwise and the reaction mixture was stirred for 1.5 h. Fmoc-Orn(Boc)-OH (778 mg, 1.71 mmol) was added in dry pyridine (3 mL) and the reaction mixture was stirred for 3 h at 40 °C. The reaction mixture was allowed to cool down to rt, diluted with EtOAc (150 mL) and washed with 10% aqueous citric acid (150 mL). The aqueous phase was re-extracted with EtOAc (75 mL). The combined organic layers were washed with 10% aqueous citric acid (100 mL), saturated aqueous NH_4Cl (2 × 100 mL) and brine (100 mL). The combined organic layers were dried with $MgSO_4$, concentrated *in vacuo* and purified with silica gel column chromatography (20 → 80 % EtOAc/n-heptane) to afford Fmoc-Orn(Boc)-6ABTC (**4**, 1.03 g, 98%) as a yellow solid. **TLC** (EtOAc/heptane, 1:1 v/v) R_F = 0.69. **^{1}H NMR** (500 MHz, $CDCl_3$) δ 9.26 (s, 1H), 8.66 (d, *J* = 2.1 Hz, 1H), 8.09 (d, *J* = 8.9 Hz, 1H), 7.76 (d, *J* = 7.6 Hz, 2H), 7.60 (t, *J* = 7.2 Hz, 2H), 7.57–7.53 (m, 1H), 7.39 (t, *J* = 7.5 Hz, 2H), 7.30 (t, *J* = 5.5 Hz, 2H), 5.76–5.71 (m, 1H), 4.87–4.83 (m, 1H), 4.68–4.64 (m, 1H), 4.42 (d, *J* = 7.1 Hz, 2H), 4.22 (t, *J* = 7.0 Hz, 1H), 3.63–3.50 (m, 1H), 3.16–3.06 (m, 1H), 2.04 (s, 2H), 1.67 (s, 2H), 1.45 (s, 9H). **^{13}C NMR** (126 MHz, $CDCl_3$) δ 171.3, 157.6, 148.7, 143.7, 141.4, 136.9, 127.9, 127.2, 125.3, 125.2, 121.0, 120.1, 120.1, 113.2, 111.5, 47.3, 28.5.

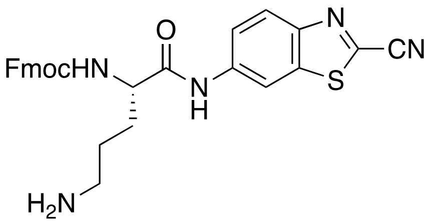

(9*H*-Fluoren-9-yl)methyl (*S*)-(5-amino-1-((2-cyanobenzo[*d*]thiazol-6-yl)amino)-1-oxopentan-2-yl)carbamate (Fmoc-Orn-6ABTC, 6): Fmoc-Orn(Boc)-6ABTC (**4**, 785, 1.28 mmol) was dissolved in HCO_2H (10 mL) and stirred overnight at rt. The reaction mixture was concentrated *in vacuo* and the product was lyophilized overnight to afford the HCO_2H salt of Fmoc-Orn-6ABTC (**6**, 733 mg, quant) as a yellow solid.

^{1}H NMR (400 MHz, CD_3OD) δ 8.64 (d, *J* = 2.1 Hz, 1H), 8.12 (d, *J* = 9.0 Hz, 1H), 7.78 (d, *J* = 7.6 Hz, 2H), 7.70 (dd, *J* = 9.0, 2.1 Hz, 1H), 7.66 (t, *J* = 7.5 Hz, 2H), 7.37 (t, *J* = 7.5 Hz, 2H), 7.33–7.24 (m, 2H), 4.42 (qd, *J* = 10.6, 6.7 Hz, 2H), 4.35–4.31 (m, 1H), 4.21 (t, *J* = 6.7 Hz, 1H), 2.97 (t, *J* = 7.3 Hz, 2H), 1.88–1.73 (m, 3H). **^{13}C NMR** (101 MHz, CD_3OD) δ 177.3, 172.9, 169.9, 158.3, 149.9, 145.1, 142.2, 140.4, 138.0, 136.8, 128.8, 128.1, 125.9, 122.3, 120.9, 114.0, 112.9, 67.9, 56.5, 39.7, 30.1, 24.2, 22.1. **HRMS** (m/z): $[M + H]^+$ calcd. for $C_{28}H_{25}N_5O_3S$: 512.1756, found 512.1752., 24.2, 22.1.

c. Fmoc-Lys-6ABTC[2, 3]

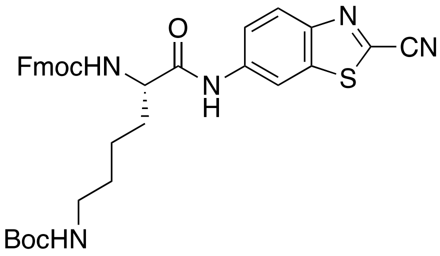

(9*H*-fluoren-9-yl)methyl *tert*-butyl (6-((2-cyanobenzo[*d*]thiazol-6-yl)amino)-6-oxohexane-1,5-diyl)(*S*)-dicarbamate (Fmoc-Lys(Boc)-6ABTC, 5): 6-Aminobenzo[*d*]thiazole-2-carbonitrile (**1**, 501 mg, 2.86 mmol) was dissolved in dry pyridine (10 mL) in a flame-dried flask. PCl_3 (130 µL, 1.49 µmol) was added dropwise and the reaction mixture was stirred for 1.5 h. Fmoc-Lys(Boc)-OH (1340 mg, 2.86 mmol) was added in dry pyridine (5 mL) and the reaction mixture was stirred for 3 h at 40 °C. The reaction mixture was allowed to cool down to rt, diluted with EtOAc (150 mL) and washed with 10% aqueous citric acid (150 mL). The aqueous phase was re-extracted with EtOAc (75 mL). The combined organic layers were washed with 10% aqueous citric acid (100 mL), saturated aqueous NH_4Cl (2 × 100 mL) and brine (100 mL). The combined organic layers were dried with $MgSO_4$, concentrated *in vacuo* and purified with silica gel column chromatography (0 → 70 % EtOAc/n-heptane) to afford Fmoc-Lys(Boc)-6ABTC (**5**, 1.38 g**,** 77%) as a yellow solid. **TLC** (EtOAc/heptane, 9:1 v/v) R_F = 0.83. **^{1}H NMR** (500 MHz, CD_3OD) δ 8.65–8.60 (m, 1H), 8.08 (dd, *J* = 9.2, 4.3 Hz, 1H), 7.79–7.74 (m, 2H), 7.69–7.63 (m, 3H), 7.39–7.34 (m, 2H), 7.32–7.25 (m, 2H), 4.40–4.36 (m, 2H), 4.28–4.24 (m, 1H), 4.22–4.18 (m, 1H), 3.07–3.00 (m, 2H), 1.89–1.82 (m, 1H), 1.80–1.71 (m, 1H), 1.55–1.47 (m, 4H), 1.39 (s, 9H). **^{13}C NMR** (126 MHz, $CDCl_3$) δ 173.7, 158.6, 149.8, 145.2, 145.1, 142.5, 140.5, 138.0, 136.7, 128.7, 128.1, 126.2, 125.9, 122.2, 120.9, 114.0, 112.8, 79.8, 67.9, 57.2, 48.4, 38.4, 32.9, 30.6, 28.7, 24.2.

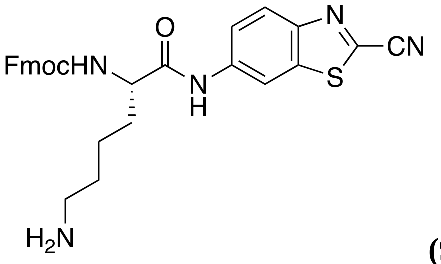

(9*H*-fluoren-9-yl)methyl (*S*)-(6-amino-1-((2-cyanobenzo[*d*]thiazol-6-yl)amino)-1-oxohexan-2-yl)carbamate (Fmoc-Lys-6ABTC, 7): Fmoc-Lys(Boc)-6ABTC (**5**, 1.38g, 2.21 mmol) was dissolved in HCO_2H (10 mL) and stirred overnight at rt. The reaction mixture was concentrated *in vacuo* and the product was lyophilized

overnight to afford the HCO_2H salt of Fmoc-Lys-6ABTC (**7**, 1260 mg, quant) as a yellow solid. **^{1}H NMR** (400 MHz, CD_3OD) δ 8.61 (d, *J* = 2.1 Hz, 1H), 8.07 (d, *J* = 9.0 Hz, 1H), 8.28 (s, 2H), 7.75 (d, *J* = 7.5 Hz, 2H), 7.70–7.60 (m, 3H), 7.35 (t, *J* = 7.5 Hz, 2H), 7.26 (t, *J* = 7.5 Hz, 2H), 4.40 (dd, *J* = 6.7, 3.4 Hz, 2H), 4.28 (dd, *J* = 8.9, 5.3 Hz, 1H), 4.19 (t, *J* = 6.7 Hz, 1H), 2.93–2.88 (m, 2H), 1.94–1.83 (m, 1H), 1.83–1.62 (m, 3H), 1.58–1.40 (m, 2H). **^{13}C NMR** (101 MHz, CD_3OD) δ 172.0, 157.2, 148.5, 143.7, 141.2, 139.1, 136.6, 135.4, 127.4, 126.7, 124.8, 124.6, 120.9, 119.6, 112.7, 111.6, 66.5, 55.6, 47.0, 39.1, 31.3, 26.8, 22.5. **HRMS** (m/z): $[M + H]^+$ calcd. $C_{29}H_{27}N_5O_3S$: 526.1912, found 526.1909.

d. Guanidinylation reagent

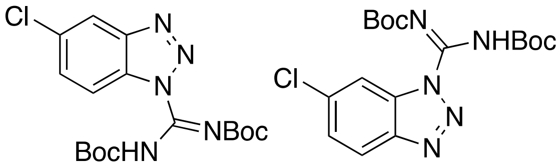

***N,N*'-di-*tert*-butoxycarbonyl-5-chloro-1*H*-benzotriazole-1-carboxamidine & *N,N*'-di-*tert*-butoxycarbonyl-6-chloro-1*H*-benzotriazole-1-carboxamidine (9)**[4, 5]: Was prepared in multiple batches. Typical procedure: *N*,*N*'-Di-Boc-thiourea (7.7 g, 27.9 mmol) and 5-chlorobenzotriazole (4.3 g, 27.9 mmol) were dissolved in anhydrous MeCN (250 mL) and DIPEA (14.6 mL, 83.6 mmol) was added. The reaction mixture was cooled to 0 °C and EDCl (10.7 g, 55.7 mmol) was added and the reaction was stirred for 18 h at rt. The mixture was diluted with EtOAc (600 mL) and washed with 10% aqueous citric acid (300 mL) and brine (300 mL). The organic layer was dried with $MgSO_4$, concentrated *in vacuo* and purified through silica gel column chromatography (0 → 15 % EtOAc/heptane) to afford the product as a mixture of the 5'- and 6' isomers (**9**, 3.49 g, 32%) as a white solid. **TLC** (EtOAc/heptane, 1:4 v/v) R_F = 0.32 (6' isomer), 0.27 (5' isomer). **^{1}H NMR** (500 MHz, $CDCl_3$) δ 8.99 (s, 2H), 8.39 (s, 1H), 8.32 (d, *J* = 8.8 Hz, 1H), 8.09 (d, *J* = 1.9 Hz, 1H), 8.03 (d, *J* = 8.8 Hz, 1H), 7.63–7.58 (m, 1H), 7.51–7.45 (m, 1H), 1.52 (d, *J* = 17.5 Hz, 27H). **^{13}C NMR** (126 MHz, $CDCl_3$) δ 131.1, 127.4, 121.1, 119.8, 116.3, 115.2, 28.1. **HRMS** (m/z): $[M + Na]^+$ calcd. for $C_{17}H_{22}ClN_5O_4Na$: 418.1258, found 418.1244.

3. Synthesis of methoxyacetyl-βAXR-6ABTC peptide conjugates (validation library)

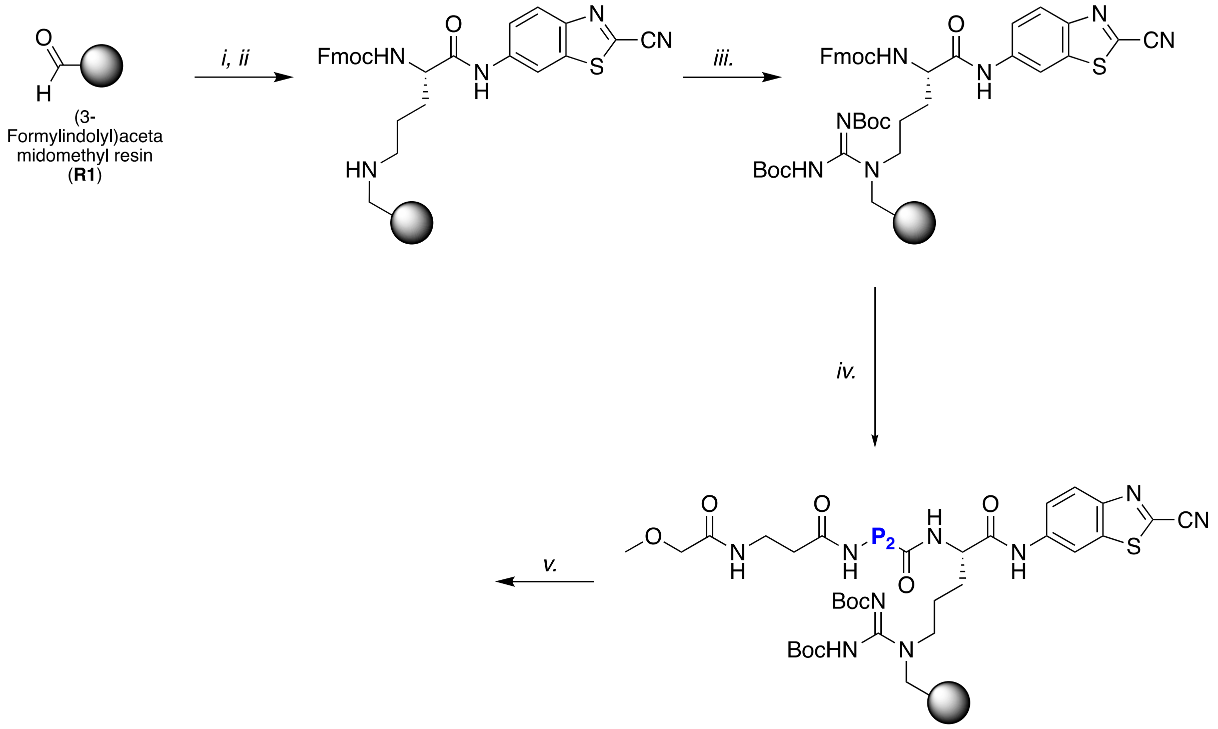

Figure S1: Synthesis of methoxyacetyl-βAXR-6ABTC peptides. i. **6** (2 equiv), THF/TMOF (1:1), 4 h, rt. ii. $NaBH_3CN$ (2 equiv), AcOH (3.5 equiv), 2 h, rt. iii. *N*,*N*'-di-Boc-thiourea (2 equiv), DIC (2 equiv), DCM, rt, 16 h. iv. a. 3% DBU in DMF, DMF (3 ×). b. DIC (3.3 equiv), HOBt (3.6 equiv) Fmoc-AA-OH / Cap-OH (methoxy acetic acid) (3.0 equiv) (3 ×), DMF. v. TFA:DCM (1:1, v/v), 2 h.

General procedure A

Typical scale 100 mg of resin:

(3-Formylindolyl)acetamidomethyl resin (100 mg, 0.073 mmol according to loading) was suspended in dry THF/TMOF (1:1, 2 mL) in a flame-dried flask. Fmoc-Orn-6ABTC (**6**, 82 mg, 0.146 mmol) was added and the suspension was stirred for 4 h at rt. $NaBH_3CN$ (9 mg, 0.146 mmol) in THF (2 mL) and AcOH (15 µL, 0.256 mmol) were added and the suspension was stirred for 2 h. The resin was washed with THF (3 ×), DCM (3 ×), MeOH (3 ×) and Et_2O (3 ×) and dried under high vacuum for 1 h. The resin was swollen in dry DCM for 20 min. *N*,*N*′-di-Boc-thiourea (40 mg, 0.146 mmol), DIPEA (25 µL, 0.146 mmol) and DIC (23 µL, 0.146 mmol) were added and the reaction was agitated overnight. The resin was washed with DCM (3 ×) and DMF (3 ×) and unreacted sites were capped by agitating with Ac_2O (138 µL, 1.46 mmol) and pyridine (118 µL, 1.46 mmol) in DMF for 10 min. The resin was washed with DMF (3 ×).

Subsequently, the resin was used for regular SPPS using standard Fmoc chemistry. Coupling of the Fmoc amino acid (3 equiv) were carried out with DIC (3.3 equiv) and HOBt (3.6 equiv) in DMF. After each coupling the resin was washed with DMF (3 ×) and the Fmoc group was removed using 3% DBU in DMF (v/v). Before final cleavage of the peptide, the resin was washed with DMF (3 ×), DCM (3 ×), MeOH (3 ×) and Et_2O (3 ×). The peptides were cleaved from the resin using 1/1 TFA:DCM for 2 h (addition of 2.5% TIS and EDT for Trt-containing sequences), concentrated *in vacuo*, lyophilized overnight and purified by RP-HPLC to obtain the target peptides.

Peptide conjugates

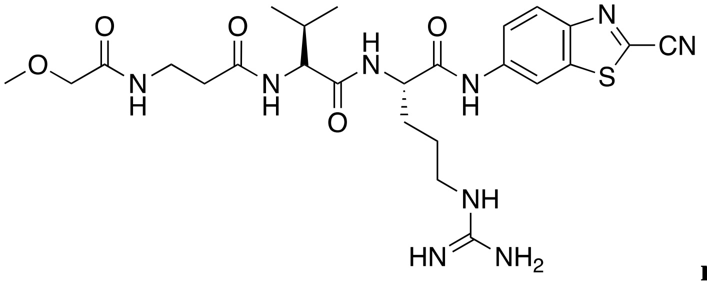

methoxyacetyl-βAVR-6ABTC: Was synthesized according to general procedure A. **^{1}H NMR** (500 MHz, CD_3OD) δ 8.14 (d, *J* = 9.0 Hz, 1H), 7.76 (dd, *J* = 9.0, 2.1 Hz, 1H), 4.58 (dd, *J* = 9.1, 4.9 Hz, 1H), 4.10 (d, *J* = 7.3 Hz, 1H), 3.86 (s, 2H), 3.49–3.43 (m, 2H), 3.37 (s, 3H), 3.25 (q, *J* = 6.6 Hz, 2H), 2.52 (t, *J* = 6.8 Hz, 2H), 2.12–1.96 (m, 3H), 1.90–1.83 (m, 1H), 1.81–1.68 (m, 3H), 1.05–0.95 (m, 6H). **^{13}C NMR** (126 MHz, CD_3OD) δ 174.4, 174.1, 172.4, 172.2, 158.6, 149.9, 140.4, 138.1, 136.9, 126.0, 122.2, 114.0, 112.8, 72.6, 61.1, 59.5, 54.8, 48.4, 42.0, 36.4, 31.4, 30.1, 26.4, 19.6. **HRMS** (m/z): $[M + H]^+$ calcd. for $C_{25}H_{35}N_9O_5S$: 574.2560, found 574.2557.

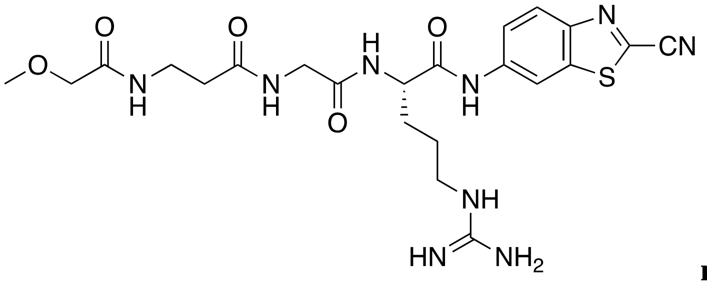

methoxyacetyl-β-AGR-6ABTC: Was synthesized according to general procedure A. **^{1}H NMR** (500 MHz, CD_3OD) δ 8.70 (d, *J* = 2.0 Hz, 1H), 8.15 (d, *J* = 9.0 Hz, 1H), 7.81 (dd, *J* = 9.0, 2.1 Hz, 1H), 4.61 (dd, *J* = 9.0, 5.0 Hz, 1H), 3.89 (d, *J* = 5.8 Hz, 2H), 3.85 (s, 2H), 3.56–3.52 (m, 2H), 3.35 (s, 3H), 3.24 (td, *J* = 7.0, 3.7 Hz, 2H), 2.52 (t, *J* = 6.6 Hz, 2H), 1.89–1.80 (m, 2H), 1.76–1.66 (m, 2H). **^{13}C NMR** (126 MHz, CD_3OD) δ 174.9, 172.4, 172.0, 158.6, 150.0, 140.4, 138.0, 137.0, 126.0, 122.4, 114.0, 113.0, 72.6, 59.5, 54.8, 44.0, 42.0, 36.5, 36.4, 30.1, 26.3, 20.7. **HRMS** (m/z): $[M + H]^+$ calcd. for $C_{22}H_{29}N_9O_5S$: 532.2090, found 532.2113.

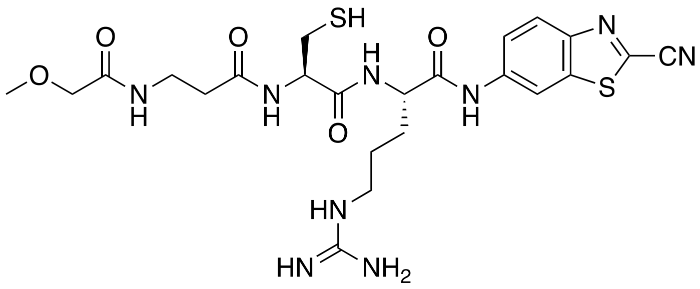

methoxyacetyl-βACR-6ABTC: Was synthesized according to general procedure A. **^{1}H NMR** (500 MHz, CD_3OD) δ 8.70 (d, *J* = 2.0 Hz, 1H), 8.14 (d, *J* = 9.0 Hz, 1H), 7.78 (dd, *J* = 9.0, 2.1 Hz, 1H), 4.59 (dd, *J* = 9.3, 4.9 Hz, 1H), 4.45 (dd, *J* = 7.0, 5.5 Hz, 1H), 3.86 (d, *J* = 1.4 Hz, 2H), 3.54 (dt, *J* = 11.0, 6.7 Hz, 2H), 3.37 (s, 3H), 3.27–3.22 (m, 2H), 2.93 (dd, *J* = 13.9, 5.6 Hz, 1H), 2.86 (dd, *J* = 13.9, 7.0 Hz, 1H), 2.53 (t, *J* = 6.7 Hz, 2H), 2.07–2.01 (m, 1H), 1.86 (dp, *J* = 14.0, 4.7 Hz, 1H), 1.81–1.68 (m, 2H). **^{13}C NMR** (126 MHz, CD_3OD) δ 174.5, 172.8, 172.6, 172.2, 158.6, 150.0, 140.3, 138.0, 136.9, 126.0, 122.3, 114.0, 112.9, 72.6, 59.5, 57.8, 55.0, 41.9, 36.6, 36.4, 30.0, 26.4, 26.4. **HRMS** (m/z): $[M + H]^+$ calcd. for $C_{23}H_{31}N_9O_5S_2$: 578.1967, found 578.1975.

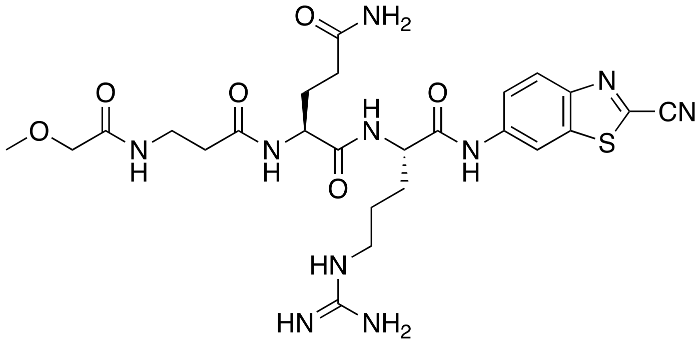

methoxyacetyl-βAQR-6ABTC: Was synthesized according to general procedure A. **^{1}H NMR** (500 MHz, CD_3OD) δ 8.70 (d, *J* = 2.1 Hz, 1H), 8.14 (d, *J* = 9.0 Hz, 1H), 7.79 (dd, *J* = 9.0, 2.1 Hz, 1H), 4.58 (dd, *J* = 9.2, 4.8 Hz, 1H), 4.30 (dd, *J* = 8.3, 6.1 Hz, 1H), 3.86 (s, 2H), 3.58–3.48 (m, 2H), 3.37 (s, 3H), 3.27–3.22 (m, 2H), 2.50 (t, *J* = 6.7 Hz, 2H), 2.37 (t, *J* = 7.4 Hz, 2H), 2.12–1.94 (m, 3H), 1.90–1.81 (m, 1H), 1.79–1.67 (m, 2H). **^{13}C NMR** (126 MHz, CD_3OD) δ 177.7, 174.3, 174.3, 172.6, 172.4, 158.7, 150.0, 140.4, 138.0, 136.9, 126.0, 122.3, 114.0, 112.9, 72.6, 59., 54.9, 54.8, 42.0, 36.6, 36.4, 32.4, 30.1, 28.4, 26.3. **HRMS** (m/z): $[M + H]^+$ calcd. for $C_{25}H_{34}N_{10}O_6S_2$: 603.2461, found 603.2458.

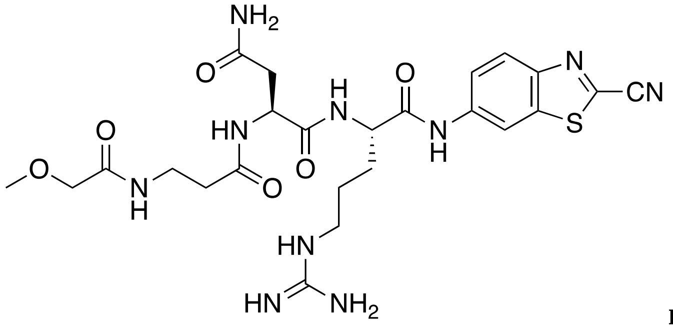

methoxyacetyl-βANR-6ABTC: Was synthesized according to general procedure A. **^{1}H NMR** (500 MHz, CD_3OD) δ 8.74 (d, *J* = 2.0 Hz, 1H), 8.13 (d, *J* = 9.0 Hz, 1H), 7.97 (dd, *J* = 9.0, 2.1 Hz, 1H), 4.65 (dd, *J* = 8.1, 5.6 Hz, 1H), 4.55 (dd, *J* = 9.8, 4.3 Hz, 1H), 3.87 (s, 2H), 3.59–3.52 (m, 2H), 3.39 (s, 3H), 3.24 (td, *J* = 6.9, 3.8 Hz, 2H), 2.84 (dd, *J* = 15.7, 8.1 Hz, 1H), 2.74 (dd, *J* = 15.7, 5.6 Hz, 1H), 2.49 (t, *J* = 6.7 Hz, 2H), 1.87–1.79 (m, 2H), 1.78–1.68 (m, 2H). **^{13}C NMR** (126 MHz, CD_3OD) δ 174.6, 174.0, 173.9, 172.5, 172.5, 158.6, 150.0, 140.5, 137.9, 136.9, 125.8, 122.7, 114.0, 113.1, 72.6, 59.5, 54.8, 52.0, 41.9, 37.4, 36.5, 36.4, 29.6, 26.3. **HRMS** (m/z): $[M + H]^+$ calcd. for $C_{24}H_{32}N_{10}O_6S$: 589.2305, found 589.2324.

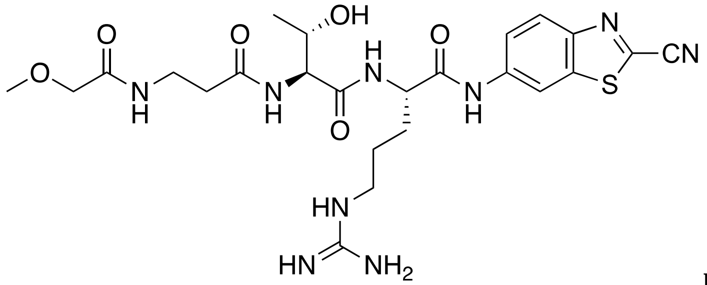

methoxyacetyl-βATR-6ABTC: Was synthesized according to general procedure A. **^{1}H NMR** (500 MHz, CD_3OD) δ 8.70 (d, *J* = 2.1 Hz, 1H), 8.14 (d, *J* = 9.0 Hz, 1H), 7.78 (dd, *J* = 9.0, 2.2 Hz, 1H), 4.62 (dd, *J* = 9.4, 4.7 Hz, 1H), 4.33 (d, *J* = 4.5 Hz, 1H), 4.23–4.17 (m, 1H), 3.86 (s, 2H), 3.56–3.51 (m, 2H), 3.37 (s, 3H), 3.25 (q, *J* = 6.7 Hz, 2H), 2.58–2.54 (m, 2H), 2.10–2.02 (m, 1H), 1.89–1.82 (m, 1H), 1.79–1.70 (m, 2H), 1.24 (d, *J* = 6.4 Hz, 3H). **^{13}C NMR** (126 MHz, CD_3OD) δ 174.5, 172.9, 172.6, 172.3, 158.5, 150.0, 140.3, 138.0, 136.9, 126.0, 122.3, 114.0, 112.9, 72.6, 68.2, 60.7, 59.5, 54.8, 41.9, 36.5, 36.5, 30.0, 26.3, 20.0. **HRMS** (m/z): $[M + H]^+$ calcd. for $C_{24}H_{33}N_9O_6S$: 576.2352, found 576.2357.

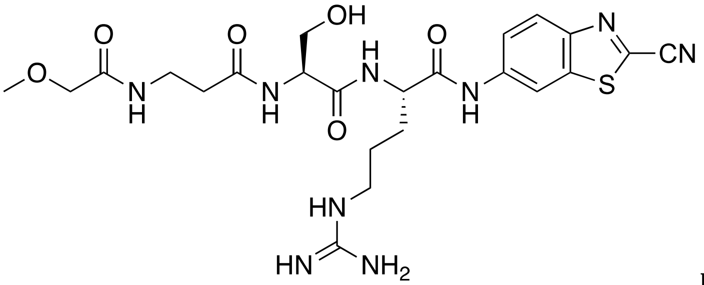

methoxyacetyl-βASR-6ABTC: Was synthesized according to general procedure A. **^{1}H NMR** (500 MHz, CD_3OD) δ 8.69 (d, *J* = 2.1 Hz, 1H), 8.14 (d, *J* = 9.0 Hz, 1H), 7.80 (dd, *J* = 9.0, 2.1 Hz, 1H), 4.62 (dd, *J* = 9.7, 4.5 Hz, 1H), 4.39 (dd, *J* = 6.3, 5.5 Hz, 1H), 3.91 (dd, *J* = 10.7, 5.5 Hz, 1H), 3.86–3.85 (m, 2H), 3.81 (dd, *J* = 10.7, 6.3 Hz, 1H), 3.56–3.51 (m, 2H), 3.37 (s, 3H), 3.25 (td, *J* = 6.9, 3.8 Hz, 2H), 2.53 (dt, *J* = 6.6, 3.2 Hz, 2H), 2.16–2.09 (m, 1H), 1.88–1.82 (m, 1H), 1.80–1.70 (m, 2H). **^{13}C NMR** (126 MHz, CD_3OD) δ 174.5, 173.3, 172.6, 172.3, 158.0, 150.0, 140.3, 138.0, 137.0, 126.0, 122.4, 114.0, 113.0, 72.6, 62.8, 59.5, 57.4, 54.8, 41.9, 36.6, 36.4, 29.7, 26.4. **HRMS** (m/z): $[M + H]^+$ calcd. for $C_{23}H_{31}N_9O_6S$: 562.2196, found 562.2206.

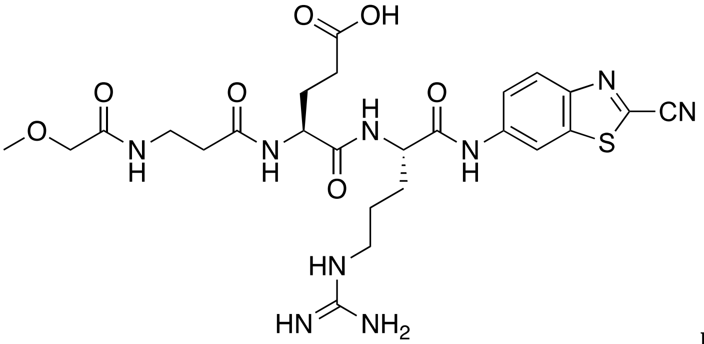

methoxyacetyl-βAER-6ABTC: Was synthesized according to general procedure A. **^{1}H NMR** (500 MHz, CD_3OD) δ 8.70 (d, *J* = 2.1 Hz, 1H), 8.14 (d, *J* = 9.0 Hz, 1H), 7.78 (dd, *J* = 9.0, 2.1 Hz, 1H), 4.58 (dd, *J* = 9.1, 4.9 Hz, 1H), 4.32 (dd, *J* = 8.4, 5.8 Hz, 1H), 3.86 (d, *J* = 0.8 Hz, 2H), 3.55–3.49 (m, 2H), 3.37 (s, 3H), 3.27–3.22 (m, 2H), 2.50 (t, *J* = 6.7 Hz, 2H), 2.48–2.41 (m, 2H), 2.14–2.06 (m, 1H), 2.06–2.00 (m, 2H), 1.85 (ddt, *J* = 13.9, 9.2, 4.7 Hz, 1H), 1.78–1.67 (m, 2H). **^{13}C NMR** (126 MHz, CD_3OD) δ 176.4, 174.4, 174.2, 172.6, 172.3, 158.6, 150.0, 140.4, 138.0, 136.9, 126.0, 122.3, 114.0, 112.9, 72.6, 59.5, 56.6, 54.8, 42.0, 36.5, 36.4, 31.1, 30.1, 27.8, 26.3. **HRMS** (m/z): $[M + H]^+$ calcd. for $C_{25}H_{33}N_9O_7S$: 604.2301, found 604.2310.

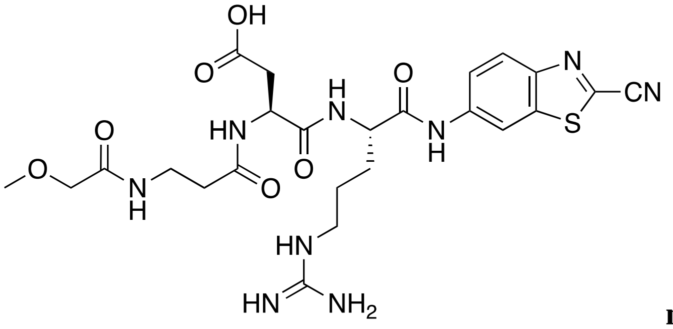

methoxyacetyl-βADR-6ABTC: Was synthesized according to general procedure A. **^{1}H NMR** (500 MHz, CD_3OD) δ 8.71 (d, *J* = 2.1 Hz, 1H), 8.13 (d, *J* = 8.9 Hz, 1H), 7.89 (dd, *J* = 9.0, 2.1 Hz, 1H), 4.66 (t, *J* = 6.3 Hz, 1H), 4.57 (dd, *J* = 9.7, 4.5 Hz, 1H), 3.87 (s, 2H), 3.45 (t, *J* = 7.1 Hz, 2H), 3.38 (s, 3H), 3.27–3.21 (m, 2H), 2.94 (dd, *J* = 17.1, 7.5 Hz, 1H), 2.82–2.78 (m, 1H), 2.49 (t, *J* = 6.8 Hz, 2H), 2.15–2.09 (m, 1H), 1.87–1.81 (m, 1H), 1.79–1.68 (m, 2H). **^{13}C NMR** (126 MHz, CD_3OD) δ 175.3, 174.3, 174.1, 173.7, 172.3, 156.4, 150.0, 140.3, 137.9, 136.9, 125.9, 122.5, 114.0, 113.1, 72.6, 59.5, 54.8, 51.8, 41.9, 36.6, 36.4, 36.3, 29.7, 26.3. **HRMS** (m/z): $[M + H]^+$ calcd. for $C_{24}H_{31}N_9O_7S$: 590.2145, found 590.2162.

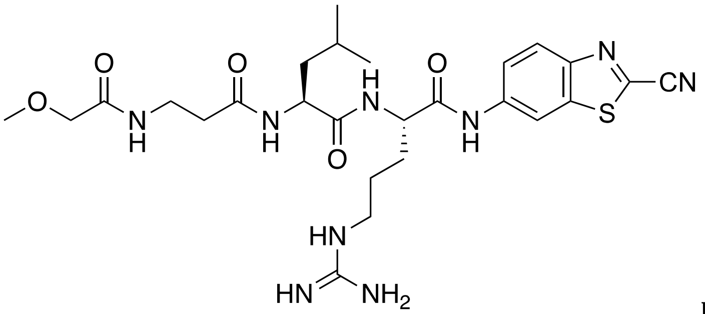

methoxyacetyl-βALR-6ABTC: Was synthesized according to general procedure A. **^{1}H NMR** (500 MHz, CD_3OD) δ 8.71 (d, *J* = 2.0 Hz, 1H), 8.14 (d, *J* = 9.0 Hz, 1H), 7.81 (dd, *J* = 9.0, 2.1 Hz, 1H), 4.58 (dd, *J* = 9.3, 4.8 Hz, 1H), 4.28 (q, *J* = 7.2 Hz, 1H), 3.85 (d, *J* = 1.9 Hz, 2H), 3.56–3.50 (m, 2H), 3.36 (s, 3H), 3.28–3.22 (m, 2H), 2.50 (td, *J* = 6.7, 2.1 Hz, 2H), 1.91–1.82 (m, 2H), 1.79–1.67 (m, 2H), 1.39 (d, *J* = 7.2 Hz, 3H). **^{13}C NMR** (126 MHz, CD_3OD) δ 175.3, 174.3, 172.5, 172.3, 158.6, 150.0, 140.4, 138.8, 137.0, 126.0, 122.2, 114.0, 112.8, 72.6, 59.5, 54.7, 53.9, 42.0, 41.5, 36.4, 36.4, 26.4, 25.9, 23.3, 21.9. **HRMS** (m/z): $[M + H]^+$ calcd. for $C_{26}H_{37}N_9O_5S$: 588.2716, found 588.2706.

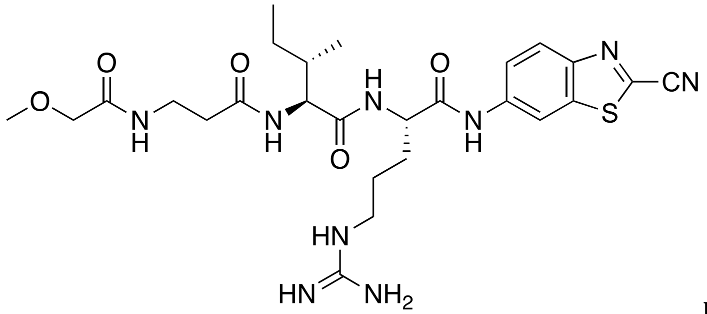

methoxyacetyl-βAIR-6ABTC: Was synthesized according to general procedure A. **^{1}H NMR** (500 MHz, CD_3OD) δ 8.72 (d, *J* = 2.1 Hz, 1H), 8.16 (dd, *J* = 9.0, 0.6 Hz, 1H), 7.78 (dd, *J* = 9.0, 2.1 Hz, 1H), 4.60 (dd, *J* = 9.2, 5.0 Hz, 1H), 4.18 (d, *J* = 7.6 Hz, 1H), 3.88–3.86 (m, 2H), 3.59–3.49 (m, 2H), 3.39 (s, 3H), 3.27 (q, *J* = 6.6 Hz, 2H), 2.55–2.51 (m, 2H), 2.08–1.98 (m, 1H), 1.92–1.83 (m, 2H), 1.80–1.68 (m, 2H), 1.60 (ddd, *J* = 13.6, 7.5, 3.6 Hz, 1H), 1.29–1.23 (m, 1H), 0.98 (d, *J* = 6.9 Hz, 3H), 0.94 (t, *J* = 7.5 Hz, 3H). **^{13}C NMR** (126 MHz, CD_3OD) δ 174.4, 174.2, 172.7, 172.5, 158.6, 149.2, 139.2, 138.1, 138.1, 126.0, 122.2, 114.0, 112.8, 72.6, 68.1, 60.0, 59.5, 42.0, 37.6, 36.5, 36.5, 30.1, 26.4, 26.2, 15.9, 11.3. **HRMS** (m/z): $[M + H]^+$ calcd. for $C_{26}H_{37}N_9O_5S$: 588.2716, found 588.2714.

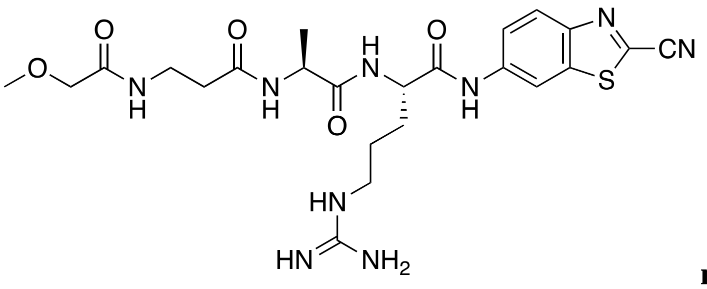

methoxyacetyl-βAAR-6ABTC: Was synthesized according to general procedure A. **^{1}H NMR** (500 MHz, CD_3OD) δ 8.71 (d, *J* = 2.0 Hz, 1H), 8.14 (d, *J* = 9.0 Hz, 1H), 7.81 (dd, *J* = 9.0, 2.1 Hz, 1H), 4.58 (dd, *J* = 9.3, 4.8 Hz, 1H), 4.28 (q, *J* = 7.2 Hz, 1H), 3.85 (d, *J* = 1.9 Hz, 2H), 3.56–3.50 (m, 2H), 3.36 (s, 3H), 3.28–3.22 (m, 2H), 2.50 (td, *J* = 6.7, 2.1 Hz, 2H), 1.91–1.82 (m, 2H), 1.79–1.67 (m, 2H), 1.39 (d, *J* = 7.2 Hz, 3H). **^{13}C NMR** (126 MHz, CD_3OD) δ 175.7, 175.2, 172.6, 172.4, 158.6, 150.0, 140.4, 138.0, 136.9, 126.0, 122.3, 114.0, 112.9, 72.6, 59.5, 54.7, 51.3, 42.0, 36.5, 36.4, 30.1, 26.3, 17.5. **HRMS** (m/z): $[M + H]^+$ calcd. for $C_{23}H_{31}N_9O_5S$: 546.2247, found 546.2246.

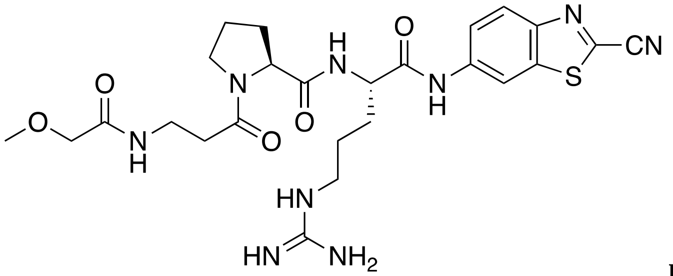

methoxyacetyl-βAPR-6ABTC: Was synthesized according to general procedure A. **^{1}H NMR** (500 MHz, CD_3OD) δ 8.72 (d, *J* = 2.2 Hz, 1H), 8.14 (d, *J* = 8.9 Hz, 1H), 7.84 (dd, *J* = 9.0, 2.1 Hz, 1H), 4.60 (dd, *J* = 9.6, 4.6 Hz, 1H), 4.44 (dd, *J* = 8.7, 4.6 Hz, 1H), 3.84 (s, 2H), 3.69–3.62 (m, 2H), 3.57–3.52 (m, 2H), 3.36 (s, 3H), 3.29–3.23 (m, 2H), 2.71–2.62 (m, 2H), 2.32–2.24 (m, 1H), 2.08–2.03 (m, 3H), 1.92–1.82 (m, 2H), 1.80–1.71 (m, 2H). **^{13}C NMR** (126 MHz, CD_3OD) δ 175.0, 173.0, 172.5, 172.3, 158.6, 150.0, 140.4, 138.0, 136.9, 126.0, 122.3, 114.0, 112.8, 72.6, 61.8, 59.5, 54.7, 48.4, 41.9, 35.8, 35.3, 30.9, 30.0, 26.4, 25.8. **HRMS** (m/z): $[M + H]^+$ calcd. for $C_{25}H_{33}N_9O_5S$: 572.2403, found 572.2397.

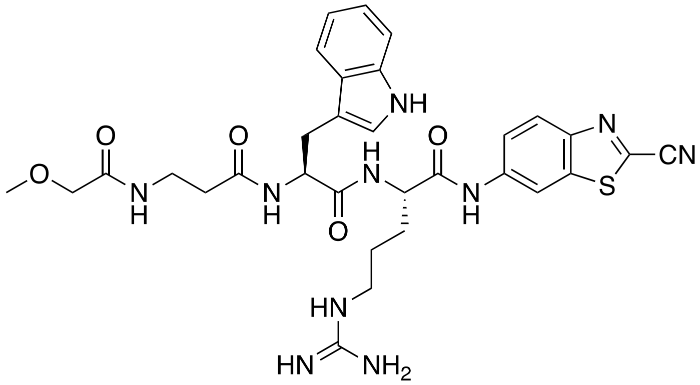

methoxyacetyl-βAWR-6ABTC: Was synthesized according to general procedure A. **^{1}H NMR** (500 MHz, CD_3OD) δ 8.68 (d, *J* = 2.1 Hz, 1H), 8.14 (d, *J* = 9.0 Hz, 1H), 7.80 (dd, *J* = 9.0, 2.1 Hz, 1H), 7.55 (d, *J* = 7.9 Hz, 1H), 7.31 (d, *J* = 8.1 Hz, 1H), 7.11 (s, 1H), 7.10–7.04 (m, 1H), 7.01–6.97 (m, 1H), 4.47 (dd, *J* = 7.9, 6.9 Hz, 1H), 4.35 (dd, *J* = 8.9, 4.4 Hz, 1H), 3.78 (s, 2H), 3.50–3.42 (m, 2H), 3.32 (s, 3H), 3.30–3.28 (m, 2H), 3.27–3.19 (m, 1H), 3.11 (dd, *J* = 14.5, 7.9 Hz, 1H), 2.46–2.33 (m, 2H), 2.06–1.98 (m, 1H), 1.78–1.69 (m, 1H), 1.63–1.53 (m, 2H). **^{13}C NMR** (126 MHz, CD_3OD) δ 175.0, 174.0, 172.5, 170.9, 158.9, 150.0, 140.3, 138.1, 138.0, 137.0, 128.6, 126.1, 124.5, 122.5, 122.3, 119.8, 119.2, 113.9, 113.0, 112.3, 110.8, 72.5, 59.5, 56.8, 56.4, 38.8, 36.5, 36.2, 30.7, 28.7, 26.3. **HRMS** (m/z): $[M + H]^+$ calcd. for $C_{31}H_{36}N_{10}O_5S$: 661.2669, found 661.2650.

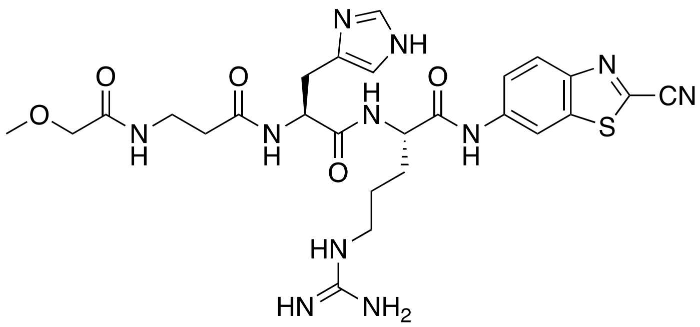

methoxyacetyl-βAHR-6ABTC: Was synthesized according to general procedure A. **^{1}H NMR** (500 MHz, CD_3OD) δ 8.80–8.77 (m, 1H), 8.68 (d, *J* = 2.1 Hz, 1H), 8.16 (d, *J* = 9.0 Hz, 1H), 7.75 (dd, *J* = 9.0, 2.1 Hz, 1H), 7.36 (s, 1H), 4.70 (t, *J* = 7.0 Hz, 1H), 4.54 (dd, *J* = 9.1, 5.0 Hz, 1H), 3.87 (s, 2H), 3.55–3.47 (m, 2H), 3.40 (s, 3H), 3.27–3.22 (m, 3H), 3.18–3.10 (m, 1H), 2.47 (t, *J* = 6.8 Hz, 2H), 1.90–1.67 (m, 4H). **^{13}C NMR** (126 MHz, CD_3OD) δ 174.0, 172.5, 172.5, 172.3, 159.2, 150.0, 141.2, 140.4, 138.1, 137.0, 126.1, 122.1, 122.1, 118.6, 114.0, 112.8, 72.6, 59.5, 55.2, 53.8, 42.0, 36.6, 36.4, 30.3, 27.9, 26.4. **HRMS** (m/z): $[M + H]^+$ calcd. for $C_{26}H_{33}N_{11}O_5S$: 612.2465, found 612.2474.

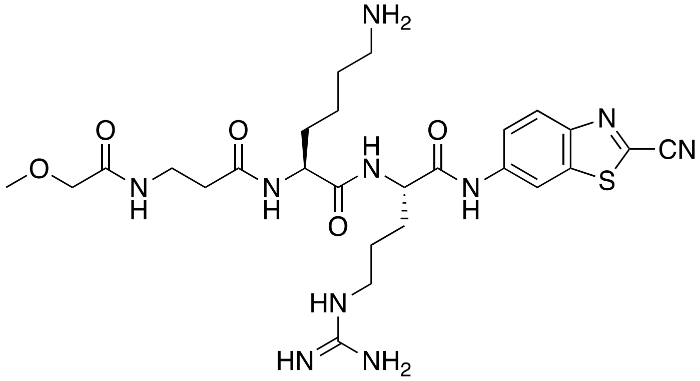

methoxyacetyl-βAKR-6ABTC: Was synthesized according to general procedure A. **^{1}H NMR** (500 MHz, CD_3OD) δ 8.67 (d, *J* = 2.1 Hz, 1H), 8.15 (d, *J* = 9.0 Hz, 1H), 7.77 (dd, *J* = 9.0, 2.1 Hz, 1H), 4.58 (dd, *J* = 9.2, 4.9 Hz, 1H), 4.31 (dd, *J* = 8.4, 6.0 Hz, 1H), 3.87 (s, 2H), 3.58–3.49 (m, 2H), 3.38 (s, 3H), 3.25 (t, *J* = 3.8 Hz, 2H), 2.92 (t, *J* = 7.6 Hz, 2H), 2.50 (t, *J* = 6.8 Hz, 2H), 1.90–1.81 (m, 3H), 1.77–1.66 (m, 5H), 1.56–1.44 (m, 2H). **^{13}C NMR** (126 MHz, CD_3OD) δ 174.5, 174.2, 172.6, 172.5, 158.6, 150.0, 140.4, 138.0, 136.9, 126.1, 122.2, 114.0, 112.8, 72.6, 59.5, 54.9, 54.9, 42.0, 40.4, 36.6, 36.4, 32.1, 30.3, 28.1, 26.4, 23.7. **HRMS** (m/z): $[M + H]^+$ calcd. for $C_{26}H_{38}N_{10}O_5S$: 603.2825, found 603.2830.

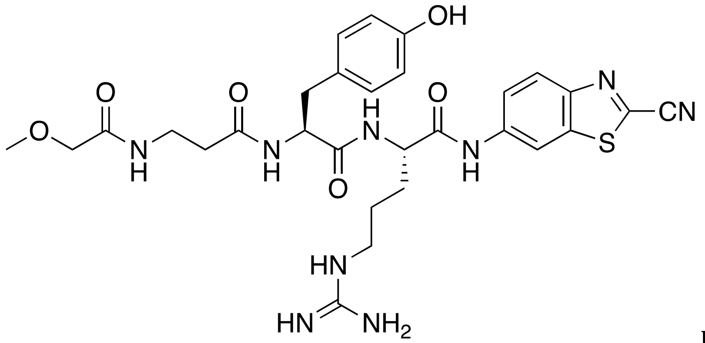

methoxyacetyl-βAYR-6ABTC: Was synthesized according to general procedure A. **^{1}H NMR** (500 MHz, CD_3OD) δ 8.69 (d, *J* = 2.0 Hz, 1H), 8.15 (d, *J* = 9.0 Hz, 1H), 7.82 (dd, *J* = 9.0, 2.1 Hz, 1H), 7.04 (d, *J* = 8.5 Hz, 2H), 6.69 (d, *J* = 8.5 Hz, 2H), 4.39–4.31 (m, 2H), 3.81–3.80 (m, 2H), 3.49–3.42 (m, 1H), 3.35 (s, 3H), 3.24–3.15 (m, 1H), 3.08–3.02 (m, 1H), 3.00–2.94 (m, 1H), 2.86–2.78 (m, 1H), 2.42–2.34 (m, 2H), 2.07–2.01 (m, 2H), 1.72–1.65 (m, 1H), 1.66–1.57 (m, 2H). **^{13}C NMR** (126 MHz, CD_3OD) δ 174.5, 173.9, 172.4, 170.8, 158.9, 157.4, 150.0, 140.3, 138.1, 137.0, 131.1, 128.8, 126.1, 122.3, 116.2, 114.0, 113.0, 72.5, 59.5, 57.5, 56.4, 38.8, 37.8, 36.4, 36.3, 30.8, 26.4. **HRMS** (m/z): $[M + H]^+$ calcd. for $C_{29}H_{35}N_9O_6S$: 638.2509, found 638.2514.

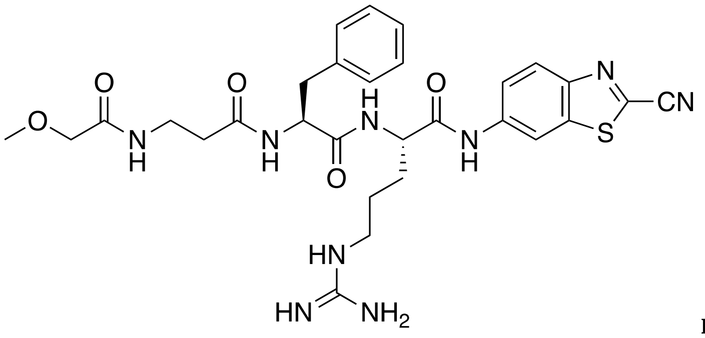

methoxyacetyl-βAFR-6ABTC: Was synthesized according to general procedure A. **^{1}H NMR** (500 MHz, CD_3OD) δ 8.70 (d, *J* = 2.1 Hz, 1H), 8.15 (d, *J* = 9.0 Hz, 1H), 7.81 (dd, *J* = 9.0, 2.1 Hz, 1H), 7.30–7.17 (m, 5H), 4.42 (dd, *J* = 8.5, 6.9 Hz, 1H), 4.37 (dd, *J* = 8.7, 4.3 Hz, 1H), 3.81 (d, *J* = 2.3 Hz, 2H), 3.48–3.40 (m, 2H), 3.35 (s, 3H), 3.10–3.01 (m, 3H), 2.92 (dd, *J* = 13.7, 8.4 Hz, 1H), 2.37 (td, *J* = 6.8, 4.8 Hz, 2H), 2.07–1.97 (m, 1H), 1.80–1.72 (m, 1H), 1.60 (d, *J* = 6.2 Hz, 2H). **^{13}C NMR** (126 MHz, CD_3OD) δ 174.3, 173.9, 172.4, 170.8, 158.9, 150.0, 140.3, 138.2, 138.1, 137.0, 130.2, 129.5, 127.8, 126.1, 122.3, 114.0, 113.0, 72.5, 59.5, 57.1, 56.4, 38.8, 38.6, 36.4, 36.2, 30.8, 26.4. **HRMS** (m/z): $[M + H]^+$ calcd. for $C_{29}H_{35}N_9O_5S$: 622.2560, found 622.2572.

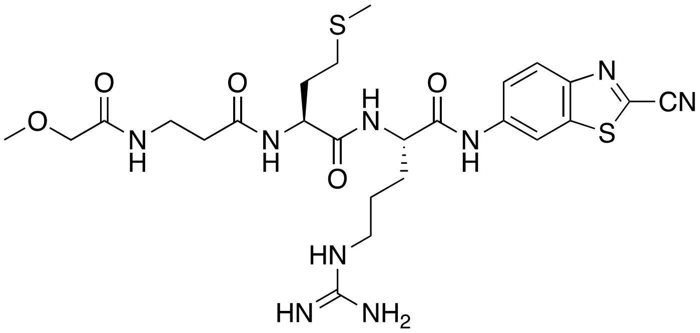

methoxyacetyl-βAMR-6ABTC: Was synthesized according to general procedure A. **^{1}H NMR** (500 MHz, CD_3OD) δ 8.70 (d, *J* = 2.1 Hz, 1H), 8.14 (d, *J* = 9.0 Hz, 1H), 7.77 (dd, *J* = 9.0, 2.1 Hz, 1H), 4.58 (dd, *J* = 9.1, 4.8 Hz, 1H), 4.41 (dd, *J* = 8.5, 5.6 Hz, 1H), 3.86 (s, 2H), 3.55–3.50 (m, 2H), 3.37 (s, 3H), 3.28–3.22 (m, 2H), 2.63–2.55 (m, 2H), 2.50 (t, *J* = 6.7 Hz, 2H), 2.08 (s, 3H), 2.05–1.95 (m, 3H), 1.90–1.82 (m, 2H), 1.78–1.69 (m, 2H). **^{13}C NMR** (126 MHz, CD_3OD) δ 174.4, 174.3, 172.6, 172.3, 158.6, 150.0, 140.4, 138.1, 136.9, 126.0, 122.2, 114.0, 112.8, 72.6, 59.5, 54.9, 54.6, 42.0, 36.5, 36.4, 32.2, 31.1, 30.1, 26.4, 15.2. **HRMS** (m/z): $[M + H]^+$ calcd. for $C_{25}H_{35}N_9O_5S_2$: 606.2280, found 606.2281.

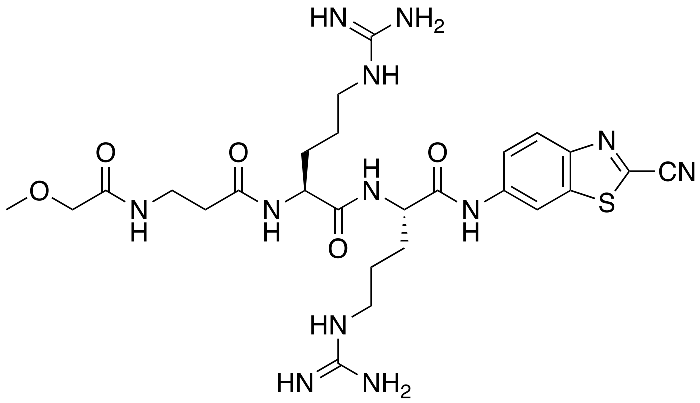

methoxyacetyl-βARR-6ABTC: Was synthesized according to general procedure A. **^{1}H NMR** (500 MHz, CD_3OD) δ 8.68 (d, *J* = 2.1 Hz, 1H), 8.17 (d, *J* = 9.0 Hz, 1H), 7.79 (dd, *J* = 9.0, 2.1 Hz, 1H), 4.59 (dd, *J* = 9.1, 5.0 Hz, 1H), 4.39–4.33 (m, 1H), 3.89 (s, 2H), 3.59–3.53 (m, 2H), 3.40 (s, 3H), 3.27 (t, *J* = 1.7 Hz, 2H), 3.22 (t, *J* = 6.9 Hz, 2H), 2.52 (t, *J* = 6.8 Hz, 2H), 1.92–1.84 (m, 2H), 1.81–1.66 (m, 6H). **^{13}C NMR** (126 MHz, CD_3OD) δ 174.2, 174.2, 172.6, 172.5, 158.6, 158.5, 150.0, 140.4, 137.8, 136.9, 126.1, 122.2, 114.0, 112.8, 72.6, 59.5, 55.0, 54.6, 42.0, 42.0, 36.6, 36.4, 30.3, 29.9, 26.4, 26.2. **HRMS** (m/z): $[M + H]^+$ calcd. for $C_{26}H_{38}N_{12}O_5S$: 631.2887, found 631.2866.

4. Synthesis of methoxyacetyl-βAXK-6ABTC peptide conjugates (validation library)

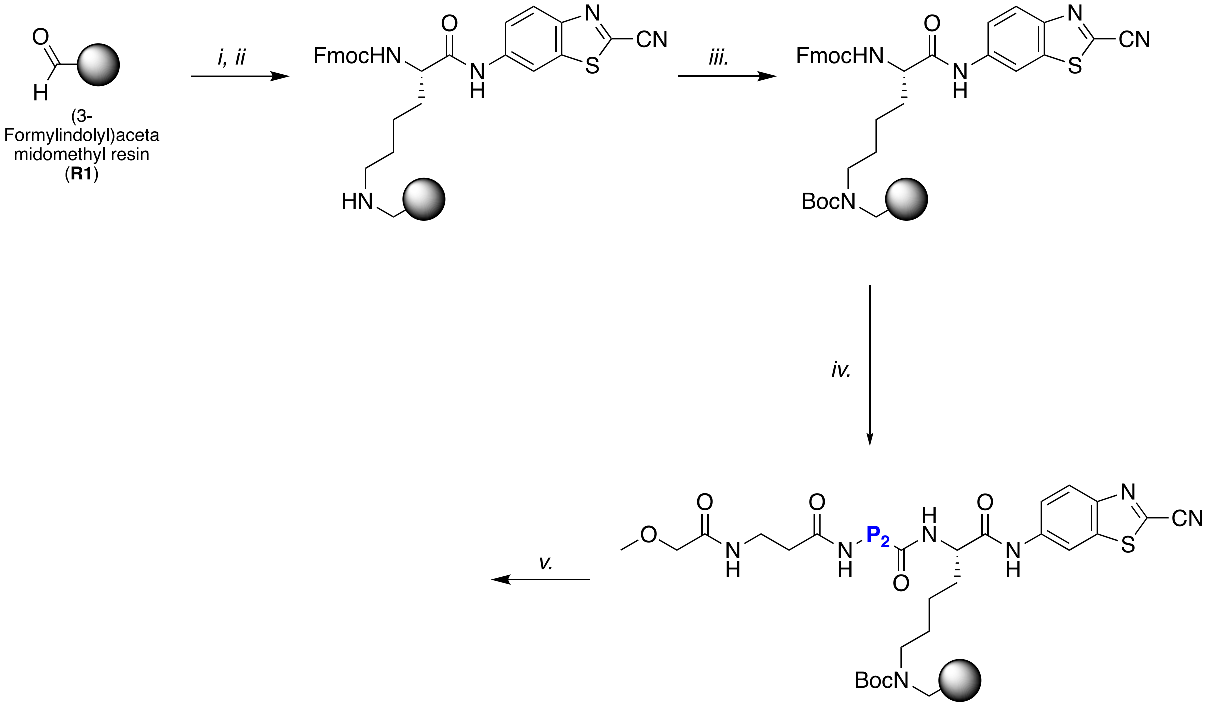

Figure S2: Synthesis of methoxyacetyl-βAXK-6ABTC peptides. i. **7** (2 equiv), THF/TMOF (1:1), 4 h, rt. ii. $NaBH_3CN$ (2 equiv), AcOH (3.5 equiv), 2 h, rt. iii. Boc_2O (3 equiv), DIPEA (3 equiv), DMF, rt, 16 h iv. 1. 3% DBU in DMF, DMF (3 ×). 2. DIC (3.3 equiv), HOBt (3.6 equiv) Fmoc-AA-OH / Cap-OH (methoxy acetic acid) (3.0 equiv) (3 ×), DMF. v. TFA:DCM (1:1, v/v), 2 h.

General Procedure B

Typical scale 100 mg of resin:

(3-Formylindolyl)acetamidomethyl resin (100 mg, 0.073 mmol according to loading) was suspended in dry THF/TMOF (1:1, 2 mL) in a flame-dried flask. Fmoc-Lys-6ABTC (**7**, 84 mg, 0.146 mmol) was added and the suspension was stirred for 4 h at rt. $NaBH_3CN$ (9 mg, 0.146 mmol) in THF (2 mL) and AcOH (15 µL, 0.256 mmol) were added and the suspension was stirred for 2 h. The resin was washed with THF (3 ×), DCM (3 ×), MeOH (3 ×) and Et_2O (3 ×) and dried under high vacuum for 1 h. The resin was swollen in DMF for 20 min. Boc_2O (48 mg, 0.219 mmol) and DIPEA (38 µL, 0.219 mmol) were added and the reaction was agitated overnight. The resin was washed with DCM (3 ×) and DMF (3 ×) and unreacted sites were capped by agitating with Ac_2O (138 µL, 1.46 mmol) and pyridine (118 µL, 1.46 mmol) in DMF for 10 min. The resin was washed with DMF (3 ×). Subsequently, the resin was used for regular

SPPS using standard Fmoc chemistry. Coupling of the Fmoc amino acid (3 equiv) were carried out using DIC (3.3 equiv) and HOBt (3.6 equiv) in DMF. After each coupling the resin was washed with DMF (3 ×) and the Fmoc group was removed using 3% DBU in DMF (v/v). Before final cleavage of the peptide the resin was washed with DMF (3 ×), DCM (3 ×), MeOH (3 ×) and Et_2O (3 ×). The peptides were cleaved from the resin using 1:1 TFA/DCM for 2 h (addition of 2.5% TIS and EDT for Trt-containing sequences), concentrated *in vacuo*, lyophilized overnight and purified by RP-HPLC to obtain the target peptides.

Peptide Conjugates

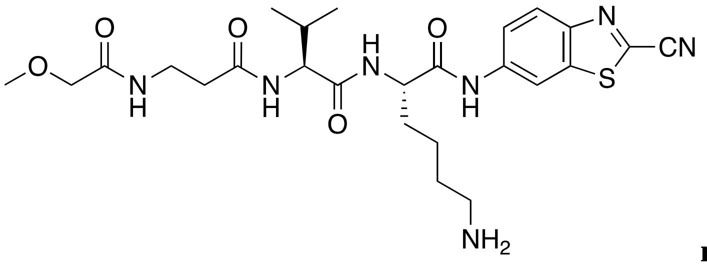

methoxyacetyl-βAVK-6ABTC: Was synthesized according to general procedure B. **^{1}H NMR** (500 MHz, D_2O) δ 8.37 (d, *J* = 2.1 Hz, 1H), 8.11 (d, *J* = 8.9 Hz, 1H), 7.68–7.63 (m, 1H), 4.51 (dd, *J* = 8.8, 5.8 Hz, 1H), 4.13 (d, *J* = 0.9 Hz, 1H), 3.96–3.95 (s, 2H), 3.54 (t, *J* = 6.6 Hz, 2H), 3.40 (s, 3H), 3.04 (t, *J* = 7.6 Hz, 2H), 2.66–2.52 (m, 2H), 2.13–2.07 (m, 1H), 2.04–1.97 (m, 1H), 1.95–1.89 (m, 1H), 1.80–1.73 (m, 2H), 1.64–1.50 (m, 2H), 1.00–0.97 (m, 6H). **^{13}C NMR** (126 MHz, D_2O) δ 174.2, 173.8, 172.4, 172.4, 148.2, 137.3, 136.8, 136.4, 124.5, 122.2, 113.5, 112.9, 70.8, 59.7, 58.9, 54.3, 39.1, 35.3, 34.9, 30.2, 29.9, 26.2, 22.1, 18.3, 17.7. **HRMS** (m/z): $[M + H]^+$ calcd. for $C_{25}H_{35}N_7O_5S$: 546.2498, found 546.2516.

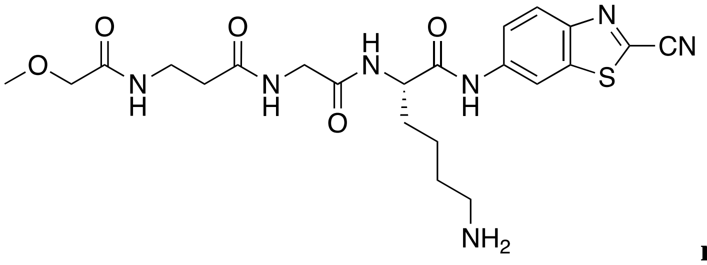

methoxyacetyl-βAGK-6ABTC: Was synthesized according to general procedure B. **^{1}H NMR** (500 MHz, D_2O) δ 8.35 (d, *J* = 2.0 Hz, 1H), 8.08 (d, *J* = 9.0 Hz, 1H), 7.64 (dd, *J* = 9.0, 2.0 Hz, 1H), 4.52 (dd, *J* = 9.0, 5.5 Hz, 1H), 4.00 (s, 2H), 3.93 (s, 2H), 3.58–3.52 (m, 2H), 3.35 (s, 3H), 3.03 (t, *J* = 7.7 Hz, 2H), 2.59 (t, *J* = 6.6 Hz, 2H), 2.08–1.96 (m, 1H), 1.93–1.87 (m, 1H), 1.79–1.70 (m, 2H), 1.61–1.48 (m, 2H). **^{13}C NMR** (126 MHz, D_2O) δ 174.7, 172.6, 172.4, 171.6, 148.2, 137.2, 136.7, 136.3, 124.4, 122.3, 113.6, 112.9, 70.7, 58.9, 54.3, 42.5, 39.1, 35.3, 34.9, 30.4, 26.2, 22.1. **HRMS** (m/z): $[M + H]^+$ calcd. for $C_{22}H_{29}N_7O_5S$: 504.2029, found 504.2035.

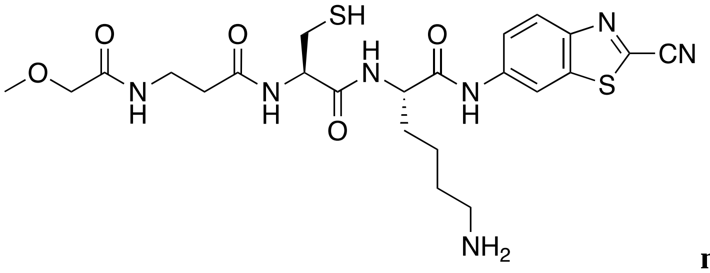

methoxyacetyl-βACK-6ABTC: Was synthesized according to general procedure B. **^{1}H NMR** (500 MHz, D_2O) δ 7.65–7.63 (m, 1H), 7.54 (d, *J* = 9.0 Hz, 1H), 7.32 (dd, *J* = 8.9, 2.2 Hz, 1H), 4.71–4.67 (m, 1H), 4.57–4.50 (m, 1H), 4.03 (s, 2H), 3.62–3.55 (m, 2H), 3.46 (s, 3H), 3.01 (t, *J* = 7.8 Hz, 4H), 2.66–2.58 (m, 2H), 1.80–1.70 (m, 4H), 1.56–1.46 (m, 2H). **^{13}C NMR** (126 MHz, D_2O) δ 173.5, 172.5, 170.8, 170.6, 147.8, 136.4, 136.2, 135.7, 124.0, 120.4, 115.1, 111.7, 70.8, 59.0, 54.1, 53.1, 39.1, 35.3, 35.0, 30.9, 26.2, 26.2, 21.9. **HRMS** (m/z): $[M + H]^+$ calcd. for $C_{23}H_{31}N_7O_5S_2$: 550.1906, found 550.1902.

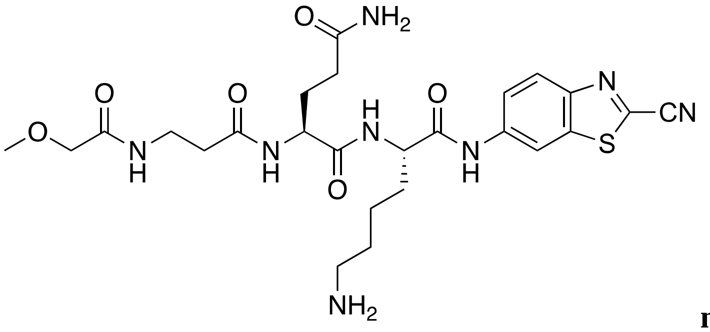

methoxyacetyl-βAQR-6ABTC: Was synthesized according to general procedure B. **^{1}H NMR** (500 MHz, D_2O) δ 8.37 (s, 1H), 8.09 (d, *J* = 8.9 Hz, 1H), 7.65 (ddd, *J* = 9.0, 2.1, 0.9 Hz, 1H), 4.51 (dd, *J* = 8.9, 5.6 Hz, 1H), 4.37 (dd, *J* = 8.7, 5.8 Hz, 1H), 3.96 (s, 2H), 3.54 (td, *J* = 6.7, 4.2 Hz, 2H), 3.40 (s, 3H), 3.05 (t, *J* = 7.6 Hz, 2H), 2.61–2.54 (m, 2H), 2.41 (t, *J* = 7.6 Hz, 2H), 2.17–2.09 (m, 1H), 2.06–1.98 (m, 2H), 1.96–1.86 (m, 1H), 1.76 (p, *J* = 7.8 Hz, 2H), 1.65–1.49 (m, 2H). **^{13}C NMR** (126 MHz, D_2O) δ 177.7, 174.1, 173.6, 172.4, 148.2, 137.3, 136.7, 136.4, 124.5, 122.2, 113.4, 112.9, 70.8, 58.9, 54.4, 53.2, 39.1, 35.3, 34.9, 31.0, 30.3, 26.8, 26.2, 22.1. **HRMS** (m/z): $[M + H]^+$ calcd. for $C_{25}H_{34}N_8O_6S_2$: 575.2400, found 575.2387.

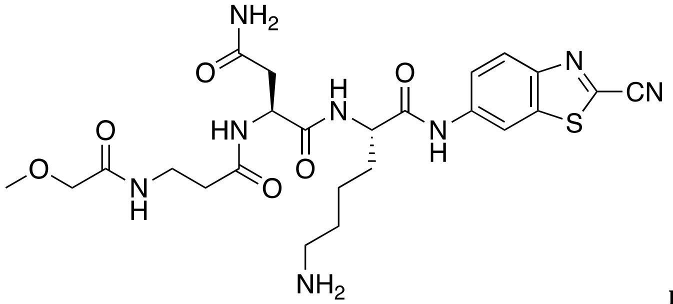

methoxyacetyl-βANK-6ABTC: Was synthesized according to general procedure B. **^{1}H NMR** (500 MHz, D_2O) δ 8.42–8.40 (m, 1H), 8.16 (d, *J* = 9.0 Hz, 1H), 7.70 (dd, *J* = 9.1, 2.1 Hz, 1H), 4.50 (dd, *J* = 9.2, 5.3 Hz, 1H), 4.08 (d, *J* = 6.0 Hz, 1H), 3.97 (s, 2H), 3.57–3.50 (m, 2H), 3.40 (s, 3H), 3.04 (t, *J* = 7.5 Hz, 2H), 2.91–2.83 (m, 1H), 2.82–2.74 (m, 1H), 2.61–2.54 (m, 2H), 1.95–1.85 (m, 2H), 1.79–1.72 (m, 2H), 1.62–1.47 (m, 2H). **^{13}C NMR** (126 MHz, D_2O) δ 174.2, 173.9, 172.9, 172.8, 172.4, 148.4, 137.2, 136.9, 136.4, 124.5, 122.5, 113.8, 112.9, 70.8, 58.9, 54.4, 50.6, 39.1, 36.2, 35.2, 34.9, 30.2, 26.2, 22.1. **HRMS** (m/z): $[M + H]^+$ calcd. for $C_{24}H_{32}N_8O_6S$: 561.2243, found 561.2246.

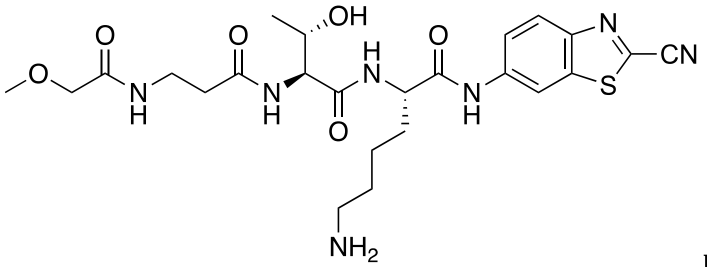

methoxyacetyl-βATK-6ABTC: Was synthesized according to general procedure B. **^{1}H NMR** (500 MHz, D_2O) δ 8.38 (d, *J* = 2.0 Hz, 1H), 8.11 (dd, *J* = 9.0, 1.2 Hz, 1H), 7.68–7.64 (m, 1H), 4.54 (dd, *J* = 8.9, 5.7 Hz, 1H), 4.35 (dd, *J* = 5.4, 1.1 Hz, 1H), 4.20 (ddd, *J* = 6.5, 5.2, 1.1 Hz, 1H), 3.96 (s, 2H), 3.55 (t, *J* = 6.6 Hz, 2H), 3.39 (s, 3H), 3.04 (t, *J* = 7.5 Hz, 2H), 2.67–2.59 (m, 2H), 2.06–1.98 (m, 1H), 1.96–1.87 (m, 1H), 1.79–1.72 (m, 2H), 1.63–1.47 (m, 2H), 1.25 (d, *J* = 1.1 Hz, 3H). **^{13}C NMR** (126 MHz, D_2O) δ 174.4, 172.4, 172.3, 172.1, 148.3, 137.2, 136.8, 136.4, 124.5, 122.3, 113.5, 112.9, 70.8, 66.9, 59.4, 58.9, 54.3, 39.1, 35.3, 34.9, 30.3, 26.2, 22.1, 18.8. **HRMS** (m/z): $[M + H]^+$ calcd. for $C_{24}H_{33}N_7O_6S$: 548.2291, found 548.2290.

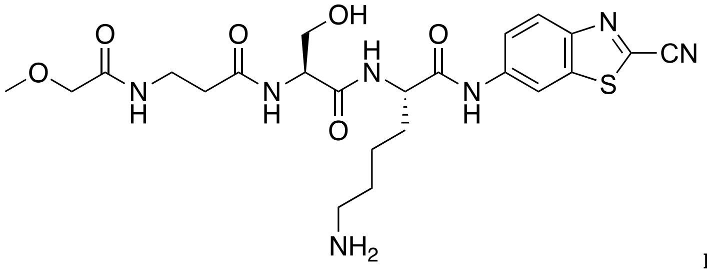

methoxyacetyl-βASK-6ABTC: Was synthesized according to general procedure B. **^{1}H NMR** (500 MHz, D_2O) δ 8.44–8.40 (m, 1H), 8.18 (d, *J* = 9.0 Hz, 1H), 7.70 (dd, *J* = 9.0, 2.1 Hz, 1H), 4.59–4.53 (m, 1H), 4.48 (t, *J* = 5.8 Hz, 1H), 3.96 (s, 2H), 3.90 (t, *J* = 5.2 Hz, 2H), 3.55 (t, *J* = 6.9 Hz, 2H), 3.39 (s, 3H), 3.04 (t, *J* = 7.3 Hz, 2H), 2.61 (t, *J* = 6.6 Hz, 2H), 2.06–2.02 (m, 1H), 1.96–1.87 (m, 1H), 1.80–1.72 (m, 2H), 1.63–1.49 (m, 2H). **^{13}C NMR** (126 MHz, D_2O) δ 174.4, 172.4, 172.4, 172.2, 148.4, 137.2, 136.9, 136.4, 124.6, 122.4, 113.7, 112.9, 70.8, 60.9, 58.9, 55.7, 54.3, 39.1, 35.3, 34.9, 30.2, 26.2, 22.1. **HRMS** (m/z): $[M + H]^+$ calcd. for $C_{23}H_{31}N_7O_6S$: 534.2134, found 534.2136.

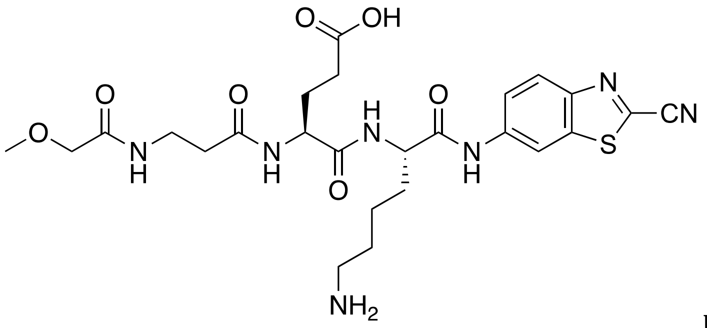

methoxyacetyl-βAEK-6ABTC: Was synthesized according to general procedure B. **^{1}H NMR** (500 MHz, D_2O) δ 8.38 (d, *J* = 2.1 Hz, 1H), 8.10 (d, *J* = 9.0 Hz, 1H), 7.66 (dd, *J* = 9.0, 2.1 Hz, 1H), 4.50 (dd, *J* = 8.8, 5.6 Hz, 1H), 4.39 (dd, *J* = 8.6, 5.9 Hz, 1H), 3.95 (s, 2H), 3.53 (t, *J* = 1.8 Hz, 2H), 3.39 (s, 3H), 3.04 (t, *J* = 7.5 Hz, 2H), 2.59–2.55 (m, 2H), 2.52 (t, *J* = 7.5 Hz, 2H), 2.18–2.13 (m, 2H), 2.04–2.02 (m, 1H), 1.94–1.89 (m, 1H), 1.78–1.72 (m, 2H), 1.62–1.51 (m, 2H). **^{13}C NMR** (126 MHz, D_2O) δ 176.8, 174.1, 173.6, 172.4, 172.4, 148.2, 137.3, 136.7, 136.4, 124.5, 122.2, 113.4, 112.9, 70.8, 58.9, 54.3, 53.1, 39.1, 35.3, 34.9, 30.3, 29.8, 26.2, 26.1, 22.1. **HRMS** (m/z): $[M + H]^+$ calcd. for $C_{25}H_{33}N_7O_7S$: 576.2240, found 576.2230.

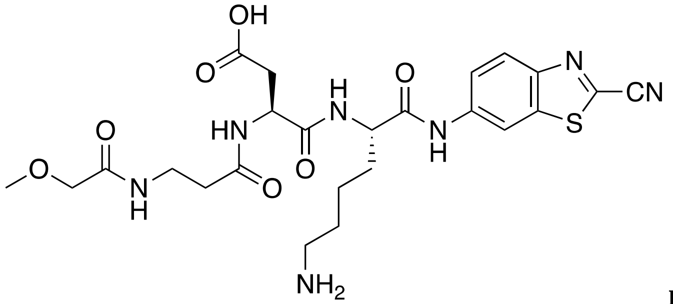

methoxyacetyl-βADK-6ABTC: Was synthesized according to general procedure B. **^{1}H NMR** (500 MHz, D_2O) δ 8.30–8.26 (m, 1H), 8.01 (d, *J* = 9.0 Hz, 1H), 7.58 (dd, *J* = 9.0, 2.1 Hz, 1H), 4.43 (dt, *J* = 9.3, 5.8 Hz, 2H), 3.87 (s, 2H), 3.49–3.42 (m, 2H), 3.31 (s, 3H), 2.95 (t, *J* = 7.5 Hz, 2H), 2.90–2.85 (m, 1H), 2.82 (d, *J* = 7.4 Hz, 1H), 2.49 (t, *J* = 6.6 Hz, 2H), 1.98–1.91 (m, 1H), 1.87–1.78 (m, 2H), 1.69–1.60 (m, 1H), 1.52–1.38 (m, 2H). **^{13}C NMR** (126 MHz, D_2O) δ 174.0, 173.9, 172.7, 172.5, 172.3, 148.3, 137.2, 136.8, 136.4, 124.5, 122.3, 113.5, 112.9, 70.8, 58.9, 54.3, 50.2, 39.1, 35.3, 35.0, 30.2, 26.2, 22.1, 21.7. **HRMS** (m/z): $[M + H]^+$ calcd. for $C_{24}H_{31}N_7O_7S$: 562.2083, found 562.2086.

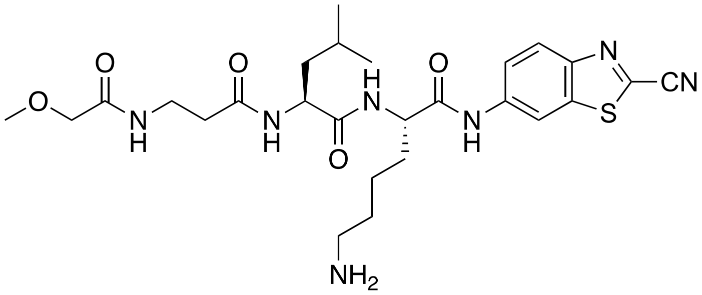

methoxyacetyl-βALK-6ABTC: Was synthesized according to general procedure B. **^{1}H NMR** (500 MHz, D_2O) δ 8.38 (d, *J* = 2.1 Hz, 1H), 8.13 (d, *J* = 9.0 Hz, 1H), 7.66 (ddd, *J* = 8.9, 2.1, 0.8 Hz, 1H), 4.52 (dd, *J* = 8.9, 5.6 Hz, 1H), 4.36 (dd, *J* = 9.2, 5.4 Hz, 1H), 3.95 (s, 2H), 3.53 (t, *J* = 6.6 Hz, 2H), 3.39 (s, 3H), 3.04 (t, *J* = 7.6 Hz, 2H), 2.63–2.50 (m, 2H), 2.07–1.96 (m, 1H), 1.95–1.86 (m, 1H), 1.78–1.72 (m, 2H), 1.69–1.60 (m, 3H), 1.57–1.48 (m, 2H), 0.96 (d, *J* = 5.8 Hz, 3H), 0.91 (d, *J* = 5.9 Hz, 3H).**^{13}C NMR** (126 MHz, D_2O) δ 175.0, 174.1, 172.4, 172.3, 148.3, 137.3, 136.8, 136.4, 124.5, 122.2, 113.5, 112.9, 70.8, 58.9, 54.2, 52.6, 39.7, 39.1, 35.3, 34.9, 30.2, 26.2, 24.3, 22.1, 22.0, 20.7. **HRMS** (m/z): $[M + H]^+$ calcd. for $C_{26}H_{37}N_7O_5S$: 560.2655, found 560.2651.

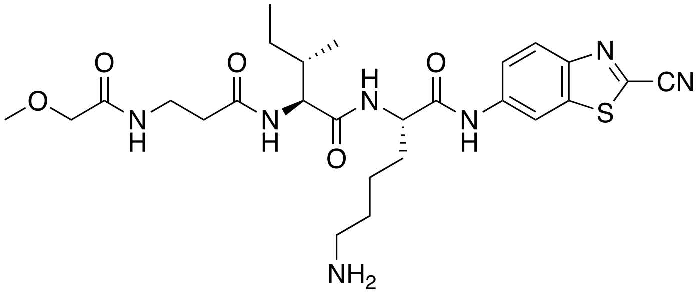

methoxyacetyl-βAIK-6ABTC: Was synthesized according to general procedure B. **^{1}H NMR** (500 MHz, D_2O) δ 8.38 (d, *J* = 2.0 Hz, 1H), 8.13 (d, *J* = 9.0 Hz, 1H), 7.66 (dd, *J* = 9.0, 2.1 Hz, 1H), 4.52 (dd, *J* = 8.7, 5.9 Hz, 1H), 4.18 (d, *J* = 7.9 Hz, 1H), 3.96 (s, 2H), 3.53 (t, *J* = 6.5 Hz, 2H), 3.40 (s, 3H), 3.04 (t, *J* = 7.7 Hz, 2H), 2.65–2.51 (m, 2H), 1.95–1.85 (m, 2H), 1.76 (ddd, *J* = 10.8, 8.9, 6.8 Hz, 2H), 1.65–1.46 (m, 4H), 1.27–1.18 (m, 1H), 0.95 (d, *J* = 6.8 Hz, 3H), 0.88 (t, *J* = 6.6 Hz, 3H). **^{13}C NMR** (126 MHz, D_2O) δ 174.1, 173.9, 172.4, 172.3, 148.3, 137.3, 136.8, 136.4, 124.6, 122.3, 113.5, 112.9, 70.8, 58.9, 58.5, 54.3, 39.1, 35.9, 35.4, 34.9, 30.2, 26.2, 24.6, 22.1, 14.7, 10.0. **HRMS** (m/z): $[M + H]^+$ calcd. for $C_{26}H_{37}N_7O_5S$: 560.2655, found 560.2644.

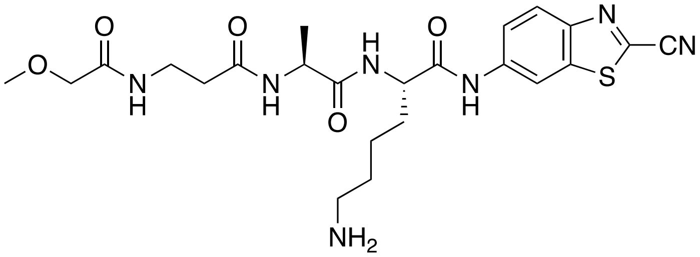

methoxyacetyl-βAAK-6ABTC: Was synthesized according to general procedure B. **^{1}H NMR** (500 MHz, D_2O) δ 8.36 (d, *J* = 2.1 Hz, 1H), 8.08 (d, *J* = 8.9 Hz, 1H), 7.65 (dd, *J* = 9.0, 2.1 Hz, 1H), 4.50 (dd, *J* = 9.1, 5.5 Hz, 1H), 4.33 (q, *J* = 7.2 Hz, 1H), 3.94 (s, 2H), 3.53 (t, *J* = 6.6 Hz, 2H), 3.38 (s, 3H), 3.04 (t, *J* = 7.6 Hz, 2H), 2.55 (td, *J* = 6.6, 2.9 Hz, 2H), 2.06–1.97 (m, 1H), 1.95–1.86 (m, 1H), 1.80–1.72 (m, 2H), 1.63–1.49 (m, 2H), 1.42 (d, *J* = 7.2 Hz, 3H). **^{13}C NMR** (126 MHz, D_2O) δ 175.4, 174.0, 172.5, 172.4, 148.2, 137.3, 136.7, 136.4, 124.5, 122.2, 113.4, 112.9, 70.7, 58.9, 54.2, 49.9, 39.2, 35.3, 34.8, 30.3, 26.6, 22.1, 15.5. **HRMS** (m/z): $[M + H]^+$ calcd. for $C_{23}H_{31}N_7O_5S$: 518.2185, found 518.2190.

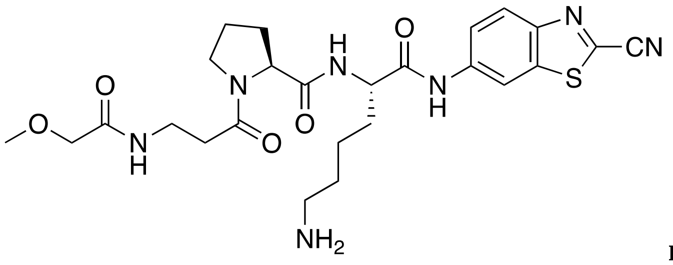

methoxyacetyl-βAPK-6ABTC: Was synthesized according to general procedure B. **^{1}H NMR** (500 MHz, D_2O) δ 8.34–8.31 (m, 1H), 8.07 (d, *J* = 8.9 Hz, 1H), 7.62 (dd, *J* = 9.0, 2.0 Hz, 1H), 4.46–4.33 (m, 2H), 3.86–3.83 (m, 2H), 3.58 (t, *J* = 6.4 Hz, 2H), 3.49–3.41 (m, 2H), 3.29 (s, 3H), 2.96 (t, *J* = 7.5 Hz, 2H), 2.68–2.61 (m, 2H), 2.27 (dd, *J* = 12.6, 7.2 Hz, 1H), 2.00–1.79 (m, 5H), 1.73–1.63 (m, 2H), 1.57–1.37 (m, 2H). **^{13}C NMR** (126 MHz, D_2O) δ 174.7, 172.6, 172.6, 172.4, 148.4, 137.3, 136.9, 136.4, 124.6, 122.3, 113.6, 112.9, 70.7, 60.3, 58.9, 54.2, 48.0, 39.2, 34.6, 33.4, 30.2, 29.7, 26.2, 24.4, 22.2. **HRMS** (m/z): $[M + H]^+$ calcd. for $C_{25}H_{33}N_7O_5S$: 544.2342, found 544.2341.

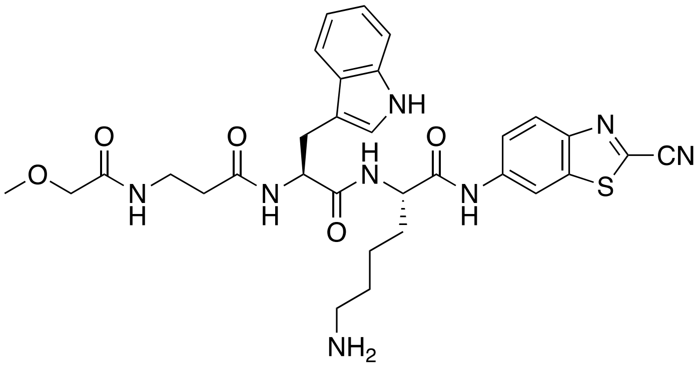

methoxyacetyl-βAWK-6ABTC: Was synthesized according to general procedure B. **^{1}H NMR** (500 MHz, D_2O) δ 8.05 (s, 1H), 7.98 (d, *J* = 8.9 Hz, 1H), 7.50 (d, *J* = 7.9 Hz, 1H), 7.40 (d, *J* = 9.0 Hz, 1H), 7.20 (s, 1H), 7.06 (d, *J* = 8.1 Hz, 1H), 6.98 (t, *J* = 7.5 Hz, 1H), 6.90 (t, *J* = 7.6 Hz, 1H), 4.59 (t, *J* = 7.6 Hz, 1H), 4.30 (dd, *J* = 8.8, 5.6 Hz, 1H), 3.90 (s, 2H), 3.48 (t, *J* = 6.6 Hz, 2H), 3.36 (d, *J* = 0.9 Hz, 3H), 3.20 (d, *J* = 7.7 Hz, 2H), 2.94 (t, *J* = 7.7 Hz, 2H), 2.54 (q, *J* = 6.5 Hz, 2H), 1.83–1.73 (m, 1H), 1.70–1.58 (m, 3H), 1.40–1.25 (m, 2H). **^{13}C NMR** (126 MHz, D_2O) δ 173.7, 173.4, 172.3, 171.2, 147.9, 137.3, 136.3, 136.2, 135.8, 126.7, 124.2, 124.1, 121.9, 121.6, 119.1, 117.9, 112.9, 112.9, 111.5, 108.5, 70.7, 58.9, 55.1, 53.8, 39.1, 35.3, 34.9, 30.8, 26.9, 26.2, 21.8. **HRMS** (m/z): $[M + H]^+$ calcd. for $C_{31}H_{36}N_8O_5S$: 633.2607, found 633.2597.

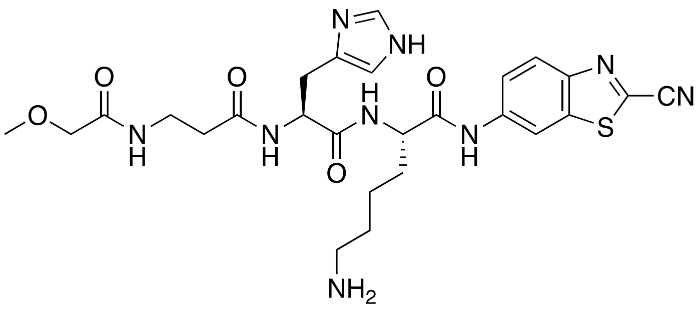

methoxyacetyl-βAHK-6ABTC: Was synthesized according to general procedure B. **^{1}H NMR** (500 MHz, D_2O) δ 8.58 (s, 1H), 8.34 (s, 1H), 8.04 (dd, *J* = 9.0, 1.2 Hz, 1H), 7.61 (dd, *J* = 9.0, 1.0 Hz, 1H), 7.30 (s, 1H), 4.74–4.69 (m, 1H), 4.52–4.46 (m, 1H), 3.95 (s, 2H), 3.49 (q, *J* = 6.4 Hz, 2H), 3.39 (s, 3H), 3.27 (dd, *J* = 15.3, 7.0 Hz, 1H), 3.17 (dd, *J* = 15.4, 8.0 Hz, 1H), 3.02 (t, *J* = 7.6 Hz, 2H), 2.53 (t, *J* = 6.7 Hz, 2H), 2.01–1.92 (m, 1H), 1.91–1.83 (m, 1H), 1.78–1.70 (m, 2H), 1.60–1.43 (m, 2H). **^{13}C NMR** (126 MHz, D_2O) δ 173.8, 172.4, 172.1, 171.7, 148.1, 137.4, 136.6, 136.4, 133.8, 128.2, 124.5, 121.9, 117.2, 113.0, 112.9, 70.8, 58.9, 54.4, 52.5, 39.1, 35.2, 34.9, 30.4, 26.3, 26.3, 22.1. **HRMS** (m/z): $[M + H]^+$ calcd. for $C_{26}H_{33}N_9O_5S$: 584.2403, found 584.2397.

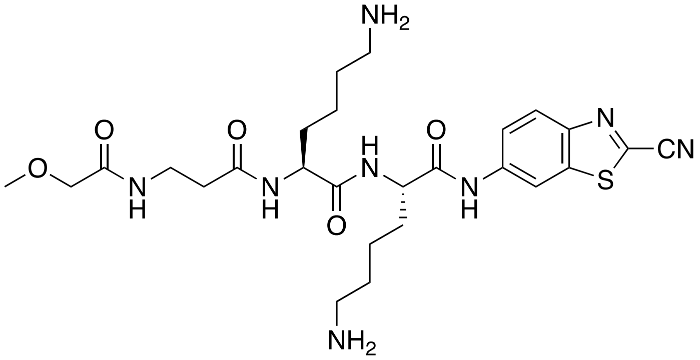

methoxyacetyl-βAKK-6ABTC: Was synthesized according to general procedure B. **^{1}H NMR** (500 MHz, D_2O) δ 8.39 (d, *J* = 2.1 Hz, 1H), 8.14 (d, *J* = 8.9 Hz, 1H), 7.68 (dd, *J* = 9.0, 2.1 Hz, 1H), 4.51 (dd, *J* = 8.8, 5.8 Hz, 1H), 4.32 (dd, *J* = 8.3, 6.1 Hz, 1H), 3.97 (s, 2H), 3.59–3.50 (m, 2H), 3.41 (s, 3H), 3.04 (t, *J* = 7.6 Hz, 2H), 2.98 (t, *J* = 7.7 Hz, 2H), 2.57 (q, *J* = 6.3 Hz, 2H), 1.95–1.82 (m, 2H), 1.82–1.65 (m, 5H), 1.65–1.42 (m, 5H). **^{13}C NMR** (126 MHz, D_2O) δ 174.1, 174.0, 172.5, 172.4, 148.3, 137.3, 136.8, 136.4, 124.6, 122.2, 113.5, 112.9, 70.8, 58.9, 54.3, 53.7, 39.1, 39.1, 35.3, 34.9, 30.4, 30.4, 27.2, 26.3, 22.1, 22.0. **HRMS** (m/z): $[M + H]^+$ calcd. for $C_{26}H_{38}N_8O_5S$: 575.2764, found 575.2751.

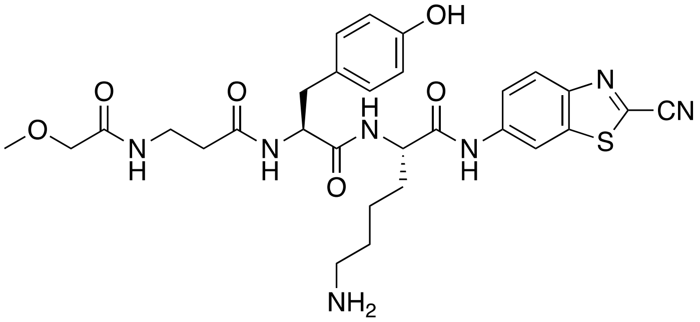

methoxyacetyl-βAYK-6ABTC: Was synthesized according to general procedure B. **^{1}H NMR** (500 MHz, D_2O) δ 8.33 (d, *J* = 2.1 Hz, 1H), 8.15 (d, *J* = 8.9 Hz, 1H), 7.58 (dd, *J* = 9.0, 2.1 Hz, 1H), 7.09 (d, *J* = 8.5 Hz, 2H), 6.60 (d, *J* = 8.4 Hz, 2H), 4.51 (dd, *J* = 9.2, 6.9 Hz, 1H), 4.41 (dd, *J* = 8.9, 5.8 Hz, 1H), 3.94 (s, 2H), 3.50 (t, *J* = 6.6 Hz, 2H), 3.40 (s, 3H), 3.04–2.97 (m, 3H), 2.91 (dd, *J* = 13.6, 9.1 Hz, 1H), 2.53 (q, *J* = 7.0 Hz, 2H), 1.92–1.82 (m, 1H), 1.80–1.66 (m, 3H), 1.50–1.35 (m, 2H). **^{13}C NMR** (126 MHz, D_2O) δ 173.7, 173.1, 172.4, 171.4, 154.4, 148.3, 137.2, 136.8, 136.5, 130.3, 127.3, 124.5, 122.2, 115.2, 113.6, 113.0, 70.7, 58.9, 55.6, 53.8, 46.6, 39.1, 36.1, 35.3, 34.8, 30.4, 26.2, 21.9. **HRMS** (m/z): $[M + H]^+$ calcd. for $C_{29}H_{35}N_7O_6S$: 610.2447, found 610.2426.

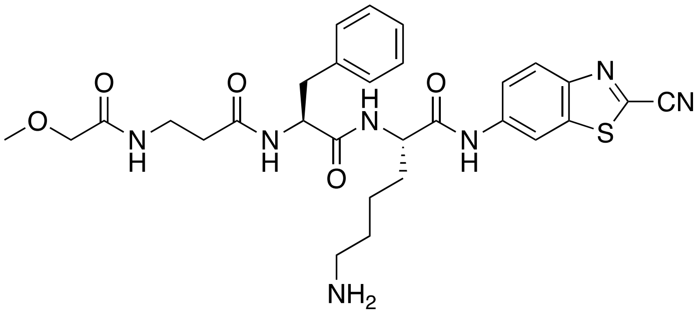

methoxyacetyl-βAFK-6ABTC: Was synthesized according to general procedure B. **^{1}H NMR** (500 MHz, D_2O) δ 8.33–8.31 (m, 1H), 8.13 (d, *J* = 8.9 Hz, 1H), 7.64–7.59 (m, 1H), 7.23 (d, *J* = 7.6 Hz, 2H), 7.19 (t, *J* = 7.5 Hz, 2H), 7.06 (t, *J* = 7.3 Hz, 1H), 4.59 (t, *J* = 7.8 Hz, 1H), 4.43 (dd, *J* = 8.8, 5.8 Hz, 1H), 3.93 (s, 2H), 3.47 (t, *J* = 6.6 Hz, 2H), 3.39 (s, 3H), 3.06 (d, *J* = 8.0 Hz, 2H), 3.01 (t, *J* = 7.5 Hz, 2H), 2.52 (td, *J* = 6.5, 3.3 Hz, 2H), 1.94–1.85 (m, 1H), 1.82–1.76 (m, 1H), 1.71 (p, *J* = 8.3 Hz, 2H), 1.51–1.36 (m, 2H). **^{13}C NMR** (126 MHz, D_2O) δ 173.7, 173.0, 172.4, 171.5, 148.2, 137.3, 136.7, 136.4, 135.8, 129.0, 128.6, 127.1, 124.5, 122.2, 113.4, 112.9, 70.7, 58.94, 55.37, 54.04, 39.16, 37.04, 35.29, 34.86, 30.41, 26.27, 21.96. **HRMS** (m/z): $[M + H]^+$ calcd. for $C_{29}H_{35}N_2O_5S$: 594.2498, found 594.2490.

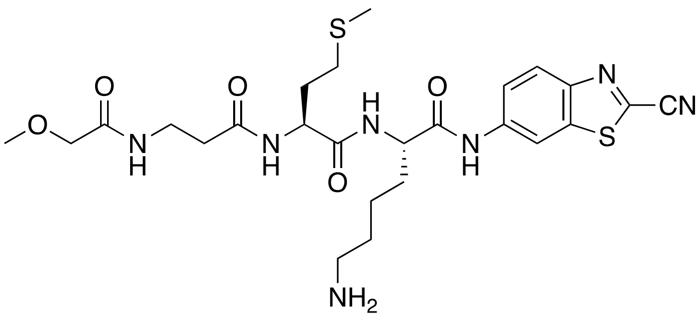

methoxyacetyl-βAMK-6ABTC: Was synthesized according to general procedure B. **^{1}H NMR** (500 MHz, D_2O) δ 8.33 (d, *J* = 2.1 Hz, 1H), 8.03 (d, *J* = 9.0 Hz, 1H), 7.61 (dd, *J* = 9.0, 2.0 Hz, 1H), 4.53 (dd, *J* = 8.8, 5.6 Hz, 1H), 4.48 (dd, *J* = 8.3, 5.9 Hz, 1H), 3.97 (s, 2H), 3.54 (t, *J* = 6.6 Hz, 2H), 3.40 (s, 3H), 3.05 (t, *J* = 7.6 Hz, 2H), 2.65–2.55 (m, 4H), 2.09 (s, 3H), 2.07–1.98 (m, 3H), 1.96–1.88 (m, 1H), 1.80–1.73 (m, 2H), 1.64–1.48 (m, 2H). **^{13}C NMR** (126 MHz, D_2O) δ 174.1, 173.7, 172.4, 172.2, 148.1, 137.4, 136.6, 136.3, 124.4, 122.0, 113.1, 112.9, 70.8, 58.9, 54.3, 52.9, 39.1, 35.3, 34.9, 30.3, 30.3, 29.2, 26.3, 22.1, 14.1. **HRMS** (m/z): $[M + H]^+$ calcd. for $C_{25}H_{35}N_7O_5S_2$: 578.2219, found 578.2205.

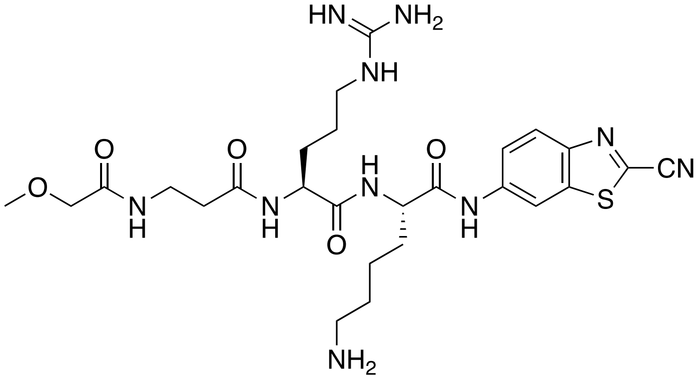

methoxyacetyl-βARK-6ABTC: Was synthesized according to general procedure B. **^{1}H NMR** (500 MHz, D_2O) δ 8.37 (d, *J* = 2.1 Hz, 1H), 8.11 (d, *J* = 9.0 Hz, 1H), 7.68–7.63 (m, 1H), 4.54–4.47 (m, 1H), 4.34 (t, *J* = 7.1 Hz, 1H), 3.96 (s, 2H), 3.57–3.50 (m, 2H), 3.40 (s, 3H), 3.16 (t, *J* = 7.0 Hz, 2H), 3.07–3.02 (m, 2H), 2.56 (q, *J* = 6.8 Hz, 2H), 2.04–1.96 (m, 1H), 1.95–1.88 (m, 1H), 1.88–1.82 (m, 1H), 1.81–1.73 (m, 3H), 1.69–1.57 (m, 3H), 1.55–1.49 (m, 1H). **^{13}C NMR** (126 MHz, D_2O) δ 174.0, 173.8, 172.4, 172.4, 156.5, 148.2, 137.3, 136.7, 136.4, 124.6, 122.1, 113.3, 112.9, 70.8, 58.9, 54.4, 53.4, 40.5, 39.1, 35.3, 34.9, 30.2, 28.1, 26.2, 23.8, 22.1. **HRMS** (m/z): $[M + H]^+$ calcd. for $C_{26}H_{38}N_{10}O_5S$: 603.2825, found 603.2813.

5. Synthesis of methoxyacetyl-XXR-6ABCT library

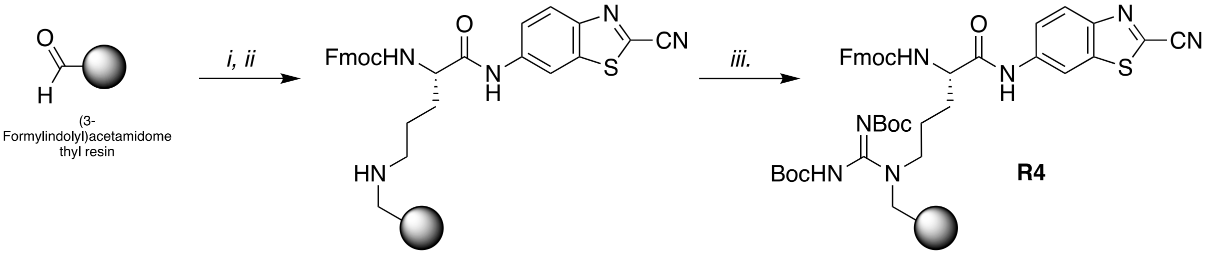

Figure S1: Loading Fmoc-Orn-6ABCT (**6**) on (3-formylindolyl)acetamidomethyl resin). i. **6** (0.9 equiv), THF:TMOF (1:1), 4 h, rt. ii. $NaBH_3CN$ (1.8 equiv), AcOH (3.5 equiv), 2 h, rt. iii. **9** (0.9 equiv), DIPEA (2.5 equiv), DMF, rt, 16 h.

<u>Loading of Fmoc-Orn-6ABCT (**2**) procedure</u>

Typical scale 10 g of resin:

(3-Formylindolyl)acetamidomethyl resin (10.00 g, 7.50 mmol according to loading) was suspended in dry THF/TMOF (1:1, 150 mL) in a flame-dried flask. Fmoc-Orn-6ABTC (**6**, 3.46 g, 6.75 mmol) was added and the suspension was agitated for 4 h at rt on a rotary evaporator. $NaBH_3CN$ (848 mg, 13.5 mmol) in THF (5 mL) and AcOH (1.5 mL, 26.2 mmol) were added and the suspension was agitated for 2 h. The resin was washed with DMF (3 ×) and DCM (3 ×) before the addition of **9** (2.82 g, 6.75 mmol) and DIPEA (3.26 mL, 18.7 mmol) in DMF (100 mL) and agitated for 18 h. Hereafter, the resin was washed with DMF (3 ×), DCM (3 ×) and Et_2O (3 ×). The resin was dried under high vacuum to afford **R4** which was used in the next steps using regular Fmoc SPPS chemistry. Multiple batches were prepared with a final loading between 0.20–0.22 mmol/g (determined by Fmoc quantification at 301 nm).

<u>SPPS reactions procedures</u>

0.03 mmol (~140–150 mg) Arg resin (loading: 0.21 mmol/g) was placed in reaction tube suitable for solid phase chemistry.

Fmoc deprotection followed by coupling of first amino acid:

DMF (1.5 ml) was added to the resin. The vial was shaken for 20 min (to swell the resin) and the solvent was removed.

Fmoc deprotection procedure:

The resin was treated with DBU (3% v/v% in DMF, freshly prepared) (1.5 ml, 0.299 mmol) and shaken for 15 min. The solvent was removed and the resin was washed with DMF (2 ×, 1.5 ml, resin was shaken 2 min in each washing step). This sequence was repeated twice (3 × DBU in total) and finally the resin was shaken with another portion DMF (3 × 1.5 mL).

Coupling procedure for P_2 and P_3:

The resin was treated with a solution of Fmoc protected amino acids (19 ×) (0.09 mmol), HOBt (0.017 g, 0.108 mmol) and DIC (0.015 ml, 0.099 mmol) in DMF (1.5 ml) (stock solutions prepared). The mixtures were shaken for 2 h. The solvent was removed and the resin was shaken with DMF (3 × 1.5 mL) and DCM (1.5 ml) (resin was shaken 2 min in each washing step) and the solvent was removed.

Coupling with capping group (P_4):

The resin was treated with a solution of methoxyacetic acid (7 µl, 0.09 mmol), HOBt (0.017 g, 0.108 mmol) and DIC (0.015 ml, 0.099 mmol) in DMF (1.5 ml) (stock solutions prepared). The mixtures were shaken for 2 h. The solvent was removed and the resin was shaken with DMF (3 × 1.5 mL) and DCM (1.5 ml) (resin was shaken 2 min in each washing step) and the solvent was removed.

Cleavage procedure :

The resin was shaken with DCM (2 × 1.5 ml) and Et_2O (2 × 1.5 ml) (resin was shaken 2 min in each washing step). The resin was shaken in a freshly prepared mixture of TFA (3.5 µl, 0.046 mmol)/triisopropylsilane (9.4 µl, 0.046 mmol) 40:1 (1.5 ml). The mixture was shaken for 2 hours. The solvent was collected in a labelled 8 ml vial. The resin was washed with TFA (3.5 µl, 0.046 mmol)/Triisopropylsilane (9.4 µl, 0.046 mmol) 40:1 (1 ml). The solvent was collected in the same 8 ml vial. The solutions were evaporated in a Genevac. The residues were dissolved in DMSO (1 ml), purified by preparative LCMS (TFA) and placed in barcoded matrix tubes. 358 compounds were shipped in matrix tubes.

Table S1: All 6ABCT conjugated peptides made in this work (358 entries) with P_1 = R and their respective amounts, molecular weight (Mw) and purity (LCMS). MAA: methoxyacetic acid. Only the P_3 and P_2 residues are depicted. General format: 6-ABCT-R-P_2-P_3-MAA, from N- to C-terminus. [a] Purity as measured by LCMS after RP-HPLC purification.

Substrate	MW	Amount (mg)	Purity[a] (%)
**-A-A-**	659,6	1,64	30,96
**-A-D-**	703,7	0,93	50,83
**-A-E-**	717,7	0,96	78,44
**-A-F-**	735,7	0,49	80,45
**-A-G-**	645,6	0,74	40,29
**-A-H-**	839,7	0,69	48,59
**-A-I-**	701,7	0,66	83,29
**-A-K-**	830,8	0,82	45,61
**-A-L-**	701,7	0,55	85,64
**-A-M-**	719,8	0,57	80,16
**-A-N-**	702,7	0,71	40,25
**-A-P-**	685,7	1,19	47,18
**-A-Q-**	716,7	0,69	37,48
**-A-R-**	858,8	0,53	37,77
**-A-S-**	675,6	0,7	42,91
**-A-T-**	689,7	1,16	41,68
**-A-V-**	687,7	0,5	65,13
**-A-W-**	774,8	0,44	66.97
**-A-Y-**	751,7	1	55,94
**-D-A-**	703,7	1,1	74,54
**-D-D-**	747,7	0,81	68,27
**-D-E-**	761,7	0,84	57,52
**-D-F-**	779,8	0,66	83,94
**-D-G-**	689,6	0,92	45,44
**-D-H-**	883,7	0,66	53,56
**-D-I-**	745,7	1,1	74,7
**-D-K-**	874,8	0,83	54,53
**-D-L-**	745,7	1,09	85,65
**-D-M-**	763,8	1,19	67,81
**-D-N-**	746,7	1,35	69,86
**-D-P-**	729,7	1,57	68,89

**-D-Q-**	760,7	1,18	67,56
**-D-R-**	902,8	0,76	69,27
**-D-S-**	719,7	1,12	73,75
**-D-T-**	733,7	1,12	48,34
**-D-V-**	731,7	1,03	72,32
**-D-W-**	818,8	0,97	88,48
**-D-Y-**	795,8	0,95	59,12
**-E-A-**	717,7	1,34	70,96
**-E-D-**	761,7	0,99	63,31
**-E-E-**	775,7	1,11	65,08
**-E-F-**	793,8	0,93	86,39
**-E-G-**	703,7	0,94	48,35
**-E-H-**	897,8	0,96	53,88
**-E-I-**	759,8	1,12	75,8
**-E-K-**	888,8	1,27	44,7
**-E-L-**	759,8	1,11	82,74
**-E-M-**	777,8	1,04	56,5
**-E-N-**	760,7	1,46	60,32
**-E-P-**	743,7	1,39	47,2
**-E-Q-**	774,7	0,7	42,5
**-E-R-**	916,8	0,32	50,9
**-E-S-**	733,7	0,52	56,91
**-E-T-**	747,7	0,43	53.29
**-E-V-**	745,7	0,57	61,74
**-E-W-**	832,8	0,32	76,82
**-E-Y-**	809,8	0,94	54,17
**-F-A-**	735,7	0,65	87,71
**-F-D-**	779,8	0,56	85,75
**-F-E-**	793,8	0,56	85,61
**-F-F-**	811,8	0,56	89,34
**-F-G-**	721,7	0,51	89,43
**-F-H-**	915,8	0,76	65,98
**-F-I-**	777,8	0,54	86,02
**-F-K-**	906,9	0,76	69,39
**-F-L-**	777,8	0,69	89,16
**-F-M-**	795,9	0,87	78,84
**-F-N-**	778,8	0,74	85,56
**-F-P-**	761,8	0,81	93,9

**-F-Q-**	792,8	0,8	62,73
**-F-R-**	934,9	0,61	59,28
**-F-S-**	751,7	0,6	74,45
**-F-T-**	765,8	0,53	85,87
**-F-V-**	763,8	0,6	97,48
**-F-W-**	850,9	0,53	89,18
**-F-Y-**	827,8	0,9	87,83
**-G-A-**	645,6	0,93	62,99
**-G-D-**	689,6	0,63	49,94
**-G-E-**	703,7	0,9	52,33
**-G-F-**	721,7	0,78	95,5
**-G-G-**	631,6	0,89	61,03
**-G-H-**	825,7	1,25	39,82
**-G-I-**	687,7	0,98	88,42
**-G-K-**	816,7	0,88	41,33
**-G-L-**	687,7	0,82	71,84
**-G-M-**	705,7	0,93	50,01
**-G-N-**	688,6	0,88	48,84
**-G-Q-**	702,7	0,93	54,57
**-G-R-**	844,8	1,13	31
**-G-S-**	661,6	0,67	55,83
**-G-T-**	675,6	0,83	66,72
**-G-V-**	673,7	0,84	87,39
**-G-W-**	760,8	0,63	85,84
**-G-Y-**	737,7	0,8	73,69
**-H-A-**	839,7	1,53	24,72
**-H-D-**	883,7	1,01	28,25
**-H-E-**	897,8	1,22	28,59
**-H-F-**	915,8	0,75	64,59
**-H-G-**	825,7	1,3	50,48
**-H-H-**	1019,8	0,76	n.d.
**-H-I-**	881,8	1,33	46,07
**-H-K-**	1010,9	1,03	n.d.
**-H-L-**	881,8	0,86	64,44
**-H-M-**	899,8	0,72	78,61
**-H-N-**	882,8	0,91	53,87
**-H-P-**	865,8	1,23	59,89
**-H-Q-**	896,8	0,83	74,88

**-H-R-**	1038,9	0,7	59,67
**-H-S-**	855,7	1,3	49,21
**-H-T-**	869,8	1,52	25,9
**-H-V-**	867,8	1,08	58,34
**-H-W-**	954,9	0,75	76,09
**-H-Y-**	931,8	1,11	66,91
**-I-A-**	701,7	1,01	70,55
**-I-D-**	745,7	0,75	95,07
**-I-E-**	759,8	0,83	72,87
**-I-F-**	777,8	0,88	86,77
**-I-G-**	687,7	0,65	83,2
**-I-H-**	881,8	1,1	63,81
**-I-I-**	743,8	0,82	87,3
**-I-K-**	872,8	1,18	50,91
**-I-L-**	743,8	0,79	84,46
**-I-M-**	761,8	0,78	84,97
**-I-N-**	744,8	0,63	86,05
**-I-P-**	727,8	1,42	65,7
**-I-Q-**	758,8	0,69	69,3
**-I-R-**	900,9	0,7	71,9
**-I-S-**	717,7	0,68	86,29
**-I-T-**	731,8	0,69	89,16
**-I-V-**	729,8	0,92	79,44
**-I-W-**	816,9	0,48	88,62
**-I-Y-**	793,8	0,98	73,69
**-K-D-**	874,8	1,64	30,86
**-K-E-**	888,8	1,55	32,27
**-K-F-**	906,9	1,05	72,68
**-K-G-**	816,7	1,27	36,34
**-K-H-**	1010,9	1,18	38,08
**-K-I-**	872,8	1,42	47,04
**-K-K-**	1001,9	1,11	n.d.
**-K-L-**	872,8	1	56,34
**-K-M-**	890,9	0,93	73,63
**-K-N-**	873,8	0,91	84,67
**-K-P-**	856,8	1,35	50,84
**-K-Q-**	887,8	0,63	82,51
**-K-R-**	1029,9	0,77	n.d.

**-K-S-**	846,8	0,89	66,23
**-K-T-**	860,8	1,52	29,12
**-K-V-**	858,8	0,96	57,05
**-K-W-**	945,9	0,85	59
**-K-Y-**	922,9	1,09	67,96
**-L-A-**	701,7	0,74	76,81
**-L-D-**	745,7	0,62	67,85
**-L-E-**	759,8	0,6	77,06
**-L-F-**	777,8	0,61	90,08
**-L-G-**	687,7	0,54	88,28
**-L-H-**	881,8	0,73	68,61
**-L-I-**	743,8	0,65	70,84
**-L-K-**	872,8	0,84	63,05
**-L-L-**	743,8	0,53	82,26
**-L-M-**	761,8	0,63	85,41
**-L-N-**	744,8	0,46	77,35
**-L-P-**	727,8	1,05	59,86
**-L-Q-**	758,8	0,67	81,17
**-L-R-**	900,9	0,78	64,85
**-L-S-**	717,7	0,66	54,8
**-L-T-**	731,8	0,63	67,3
**-L-V-**	729,8	0,61	76,14
**-L-W-**	816,9	0,57	85,7
**-L-Y-**	793,8	0,84	83
**-M-A-**	719,8	0,74	81,39
**-M-D-**	763,8	0,73	58,02
**-M-E-**	777,8	0,89	55,76
**-M-F-**	795,9	0,98	72,71
**-M-G-**	705,7	0,7	59,97
**-M-H-**	899,8	1,03	62,23
**-M-I-**	761,8	0,95	71,98
**-M-K-**	890,9	1,42	42,17
**-M-L-**	761,8	0,83	82,62
**-M-M-**	779,9	0,97	78,56
**-M-N-**	762,8	0,83	65,43
**-M-P-**	745,8	1,08	56,12
**-M-Q-**	776,8	0,86	65,81
**-M-R-**	918,9	1,16	69,33

**-M-S-**	735,8	0,88	63,27
**-M-T-**	749,8	0,84	42,86
**-M-V-**	747,8	1,05	78,45
**-M-W-**	834,9	0,84	75,31
**-M-Y-**	811,9	0,96	67,51
**-N-A-**	702,7	1,12	44,19
**-N-D-**	746,7	0,66	52,1
**-N-E-**	760,7	0,81	48,32
**-N-F-**	778,8	0,84	50,32
**-N-G-**	688,6	0,93	35,18
**-N-H-**	882,8	0,91	53,8
**-N-I-**	744,8	0,87	49,28
**-N-K-**	873,8	0,93	64,87
**-N-L-**	744,8	0,95	61,08
**-N-M-**	762,8	1,22	48,56
**-N-N-**	745,7	1,02	50,24
**-N-P-**	728,7	1,48	45,45
**-N-Q-**	759,7	1,51	44,78
**-N-R-**	901,8	1,34	31,78
**-N-S-**	718,7	1,14	36,28
**-N-T-**	732,7	0,81	45,3
**-N-V-**	730,7	1,25	30,46
**-N-W-**	817,8	0,63	61,1
**-N-Y-**	794,8	1,05	46,28
**-P-A-**	685,7	1,33	63,31
**-P-D-**	729,7	0,95	65,4
**-P-E-**	743,7	1,08	67,2
**-P-F-**	761,8	0,96	80,85
**-P-G-**	671,7	1,92	52,13
**-P-H-**	865,8	1,6	58,29
**-P-I-**	727,8	1,18	66,15
**-P-K-**	856,8	1,72	50,28
**-P-L-**	727,8	1,4	73,49
**-P-M-**	745,8	1,47	73,62
**-P-N-**	728,7	0,67	65,34
**-P-P-**	711,7	1,48	62,17
**-P-Q-**	742,7	1,15	58,98
**-P-R-**	884,8	1,45	52,12

**-P-S-**	701,7	1,64	38,93
**-P-T-**	715,7	0,94	51,81
**-P-V-**	713,7	0,93	86,45
**-P-W-**	800,8	0,88	87,48
**-P-Y-**	777,8	1,35	55,64
**-Q-A-**	716,7	1,99	45,33
**-Q-D-**	760,7	1,23	61,05
**-Q-E-**	774,7	2,04	41,49
**-Q-F-**	792,8	1,62	55,29
**-Q-G-**	702,7	1,32	50,02
**-Q-H-**	896,8	1,19	84,3
**-Q-I-**	758,8	1,71	55,07
**-Q-K-**	887,8	0,37	85,62
**-Q-L-**	758,8	1,59	60,07
**-Q-M-**	776,8	1,82	48,14
**-Q-N-**	759,7	1,93	37,26
**-Q-P-**	742,7	1,23	59,47
**-Q-Q-**	773,8	2,53	34,84
**-Q-R-**	915,8	1,43	49,9
**-Q-S-**	732,7	1,27	48,76
**-Q-T-**	746,7	1,87	40,24
**-Q-V-**	744,8	1,3	65,58
**-Q-W-**	831,8	1,24	70,76
**-Q-Y-**	808,8	1,22	83,11
**-R-A-**	858,8	0,75	46,62
**-R-D-**	902,8	0,39	46,67
**-R-E-**	916,8	0,41	46,6
**-R-F-**	934,9	0,57	56,48
**-R-G-**	844,8	0,98	30,9
**-R-H-**	1038,9	0,5	71,06
**-R-I-**	900,9	0,82	60,59
**-R-K-**	1029,9	0,65	75,61
**-R-L-**	900,9	0,71	50,13
**-R-M-**	918,9	0,92	31,64
**-R-N-**	901,8	0,92	22,94
**-R-P-**	884,8	1,22	51,01
**-R-R-**	1057,9	0,51	71,64
**-R-Q-**	915,8	0,71	41,45

**-R-S-**	874,8	1,08	27,51
**-R-T-**	888,8	0,59	34,87
**-R-V-**	886,8	0,57	63,3
**-R-W-**	973,9	0,48	61,04
**-R-Y-**	950,9	0,43	61,18
**-S-A-**	675,6	0,96	58,53
**-S-D-**	719,7	0,89	62,26
**-S-E-**	733,7	0,91	69,51
**-S-F-**	751,7	1,06	64,7
**-S-G-**	661,6	0,87	61,29
**-S-H-**	855,7	1,22	56,47
**-S-I-**	717,7	0,88	86,56
**-S-K-**	846,8	0,9	69,51
**-S-L-**	717,7	1,33	44,36
**-S-M-**	735,8	0,86	53,19
**-S-N-**	718,7	0,99	48,41
**-S-P-**	701,7	0,85	59,14
**-S-Q-**	732,7	1,01	54,48
**-S-R-**	874,8	1,67	32,27
**-S-S-**	691,6	0,81	58,86
**-S-T-**	705,7	0,91	72,77
**-S-V-**	703,7	0,87	56,29
**-S-W-**	790,8	0,95	77,42
**-S-Y-**	767,7	1,26	51,08
**-T-A-**	689,7	1,94	57,53
**-T-D-**	733,7	1,13	77,8
**-T-E-**	747,7	1,01	75,52
**-T-F-**	765,8	1,09	94,75
**-T-G-**	675,6	0,89	57,71
**-T-H-**	869,8	2,02	43,07
**-T-I-**	731,8	1,74	76,36
**-T-K-**	860,8	2,32	34,57
**-T-L-**	731,8	1,32	58,73
**-T-M-**	749,8	1,51	53,31
**-T-N-**	732,7	1,99	53,93
**-T-P-**	715,7	2,72	57,89
**-T-Q-**	746,7	2	49,07
**-T-R-**	888,8	1,54	49,24

**-T-S-**	705,7	1,67	59
**-T-T-**	719,7	1,06	80,96
**-T-V-**	717,7	1,67	62,39
**-T-W-**	804,8	1,43	79,69
**-T-Y-**	781,8	1,07	75,54
**-V-A-**	687,7	1,13	55,82
**-V-D-**	731,7	1,06	58,74
**-V-E-**	745,7	1,01	53,29
**-V-F-**	763,8	0,79	71,3
**-V-G-**	673,7	1,16	51,65
**-V-H-**	867,8	1,45	34,03
**-V-I-**	729,8	0,99	58,81
**-V-K-**	858,8	1,06	45,64
**-V-L-**	729,8	0,9	67,84
**-V-M-**	747,8	0,88	65,7
**-V-N-**	730,7	0,99	48,64
**-V-P-**	713,7	1,44	60,48
**-V-Q-**	744,8	1,55	44
**-V-R-**	886,8	1,25	31,68
**-V-S-**	703,7	1,11	51,52
**-V-T-**	717,7	1,15	56,48
**-V-V-**	715,8	1,01	70,08
**-V-W-**	802,8	0,65	82,14
**-V-Y-**	779,8	0,8	72,32
**-W-A-**	774,8	1,52	86,05
**-W-D-**	818,8	0,57	91,64
**-W-E-**	832,8	1,1	94,67
**-W-F-**	850,9	1,52	86,39
**-W-G-**	760,8	1,4	77,15
**-W-H-**	954,9	1,85	65,68
**-W-I-**	816,9	1,37	90,58
**-W-K-**	945,9	1,82	59,78
**-W-L-**	816,9	1,42	89,47
**-W-M-**	834,9	1,38	81,45
**-W-N-**	817,8	1,69	68,01
**-W-P-**	800,8	1,89	87,7
**-W-Q-**	831,8	2,06	60,5
**-W-R-**	973,9	1,71	68,76

**-W-S-**	790,8	1,41	75,06
**-W-T-**	804,8	1,41	78,88
**-W-V-**	802,8	1,38	88,21
**-W-W-**	889,9	1,26	91,15
**-W-Y-**	866,9	1,27	89,28
**-Y-A-**	751,7	1,12	65,44
**-Y-D-**	795,8	1,02	70,24
**-Y-E-**	809,8	0,95	74,5
**-Y-F-**	827,8	0,83	86,03
**-Y-G-**	737,7	0,88	77,68
**-Y-H-**	931,8	1,19	78,87
**-Y-I-**	793,8	1,51	57,26
**-Y-K-**	922,9	1,27	71,18
**-Y-L-**	793,8	1,46	75,18
**-Y-M-**	811,9	1,25	63,74
**-Y-N-**	794,8	2,26	42,42
**-Y-P-**	777,8	2,38	59,35
**-Y-Q-**	808,8	1,41	59,48
**-Y-R-**	950,9	1,21	57,4
**-Y-T-**	781,8	0,62	36,85
**-Y-V-**	779,8	1,34	42,98
**-Y-W-**	866,9	1,01	81,61
**-Y-Y-**	843,8	1,58	56,47

6. Synthesis of methoxyacetyl-XXK-6ABCT library

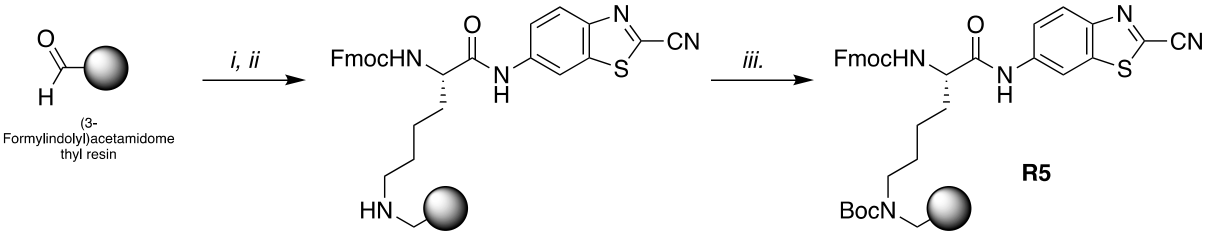

Figure S2: Loading Fmoc-Lys-6ABCT (**7**) on (3-formylindolyl)acetamidomethyl resin). i. **7** (0.9 equiv), THF:TMOF (1:1), 4 h, rt. ii. $NaBH_3CN$ (1.8 equiv), AcOH (3.5 equiv), 2 h, rt. iii. Boc_2O (3 equiv), DIPEA (3 equiv), DMF, rt, 16 h.

Loading of Fmoc-Lys-6ABCT (**3**) procedure

Typical scale 10 g of resin:

(3-Formylindolyl)acetamidomethyl resin (10.00 g, 7.50 mmol according to loading) was suspended in dry THF/TMOF (1:1, 150 mL) in a flame-dried flask. Fmoc-Lys-6ABTC (**7**, 3.55 g, 6.75 mmol) was added and the suspension was agitated for 4 h at rt on a rotary evaporator. $NaBH_3CN$ (848 mg, 13.5 mmol) in THF (5 mL) and AcOH (1.5 mL, 26.2 mmol) were added and the suspension was agitated for 2 h. The resin was washed with DMF (3 ×) and DCM (3 ×) before the addition of Boc_2O (4.91 g, 22.5 mmol) and DIPEA (3.92 mL, 22.5 mmol) in DMF (100 mL), after which the mixture was agitated for 18 h. Hereafter, the resin was washed with DMF (3 ×), DCM (3 ×) and Et_2O (3 ×). The resin was dried under high vacuum to afford **R5** which was used in the next steps with regular Fmoc SPPS chemistry. Multiple batches were prepared with a final loading between 0.16–0.25 mmol/g (determined by Fmoc quantification at 301 nm).

SPPS reactions procedures

0.046 mmol (~160–165 mg, range 0.045 mmol–0.047 mmol) Lys resin (loading: 0.287 mmol/g) was placed in reaction tube suitable for solid phase chemistry.

Fmoc deprotection followed by coupling of first amino acid :

DMF (1.5 ml) was added to the resin. The vial was shaken for 20 min (to swell the resin) and the solvent was removed.

Fmoc deprotection procedure:

The resin was treated with DBU (3% v/v% in DMF, freshly prepared) (1.5 ml, 0.299 mmol) and shaken for 15 min. The solvent was removed and the resin was washed with DMF (2 ×, 1.5 ml, resin was shaken 2 min in each washing step). This sequence was repeated twice (3 × DBU in total) and finally the resin was shaken with another portion DMF (3 × 1.5 mL).

Coupling procedure for P_2 and P_3:

The resin was treated with a solution of Fmoc protected amino acids (19 ×) (0.138 mmol), HOBt (0.032 g, 0.166 mmol) and DIC (0.024 ml, 0.152 mmol) in DMF (1.5 ml) (stock solutions prepared). The mixtures were shaken for 2 h. The solvent was removed and the resin was shaken with DMF (3 × 1.5 mL) and DCM (1.5 ml) (resin was shaken 2 min in each washing step) and the solvent was removed.

Coupling with capping group (P_4):

The resin was treated with a solution of methoxyacetic acid (11 µl, 0.138 mmol), HOBt (0.032 g, 0.166 mmol) and DIC (0.024 ml, 0.152 mmol) in DMF (1.5 ml) (stock solutions prepared). The mixtures were shaken for 2 h. The solvent was removed and the resin was shaken with DMF (3 × 1.5 mL) and DCM (1.5 ml) (resin was shaken 2 min in each washing step) and the solvent was removed.

Cleavage procedure:

The resin was shaken with DCM (2 × 1.5 ml) and Et_2O (2 × 1.5 ml) (resin was shaken 2 min in each washing step). The resin was shaken in a freshly prepared mixture of TFA (3.5 µl, 0.046 mmol)/triisopropylsilane (9.4 µl, 0.046 mmol) 40:1 (1.5 ml). The mixture was shaken for 2 hours. The solvent was collected in a labelled 8 ml vial. The resin was washed with TFA (3.5 µl, 0.046 mmol)/Triisopropylsilane (9.4 µl, 0.046 mmol) 40:1 (1 ml). The solvent was collected in the same 8 ml vial. The solutions were evaporated in a Genevac. The residues were dissolved in DMSO (1 ml), purified by preparative LCMS (TFA) and placed in barcoded matrix tubes. 361 compounds were shipped in matrix tubes.

Table S2: 6ABCT conjugated peptides made in this work (361 entries) with P_1 = K and their respective amounts, molecular weight (MW) and purity (LCMS). MAA: methoxyacetic acid. Only the P_3 and P_2 residues are depicted. General format: 6-ABCT-K-P_2-P_3-MAA, from N- to C-terminus. [a] Purity as measured by LCMS after RP-HPLC purification.

Substrate	MW	Amount (mg)	Purity[a] (%)
**-A-A-**	631,6	3,75	83,54
**-A-D-**	675,6	2,47	64,34
**-A-E-**	689,7	2,66	67,05
**-A-F-**	707,7	2,75	86,87
**-A-G-**	617,6	2,98	71,02
**-A-H-**	811,7	3,02	90,93
**-A-I-**	673,7	2,21	83,43
**-A-K-**	802,8	2,6	90,34
**-A-L-**	673,7	2,79	87,67
**-A-M-**	691,7	2,44	89,68
**-A-N-**	674,7	2,46	77,75
**-A-P-**	657,7	3,6	85,43
**-A-Q-**	688,7	2,52	83,37
**-A-R-**	830,8	3	87,6
**-A-S-**	647,6	2,76	64,68
**-A-T-**	661,7	2,78	65,04
**-A-V-**	659,7	2	88,2
**-A-W-**	746,8	2,08	76,98
**-A-Y-**	723,7	3,95	88,82
**-D-A-**	675,6	2,83	66,54
**-D-D-**	719,7	2,77	68,19
**-D-E-**	733,7	3,01	63,33
**-D-F-**	751,7	2,19	84,39
**-D-G-**	661,6	3,2	80,51
**-D-H-**	855,7	2,28	87,05
**-D-I-**	717,7	2,28	81,44
**-D-K-**	846,8	2,48	91,43
**-D-L-**	717,7	2,81	77,99
**-D-M-**	735,8	2,56	79,57
**-D-N-**	718,7	3,1	66,19
**-D-P-**	701,7	3,86	72,98

**-D-Q-**	732,7	2,98	81,09
**-D-R-**	874,8	2,6	87,1
**-D-S-**	691,6	3	79,11
**-D-T-**	705,7	3,02	49,77
**-D-V-**	703,7	2,69	71,84
**-D-W-**	790,8	2,35	88,98
**-D-Y-**	767,7	3,31	79,12
**-E-A-**	689,7	2,95	63,45
**-E-D-**	733,7	2,69	66,73
**-E-E-**	747,7	3,06	69,63
**-E-F-**	765,8	2,56	86,9
**-E-G-**	675,6	3,35	79,75
**-E-H-**	869,8	3,11	84,7
**-E-I-**	731,8	3,54	83,21
**-E-K-**	860,8	2,7	86,93
**-E-L-**	731,8	3,56	84,37
**-E-M-**	749,8	2,88	83,19
**-E-N-**	732,7	3,51	72,15
**-E-P-**	715,7	5,33	74,43
**-E-Q-**	746,7	4,16	83,4
**-E-R-**	888,8	3,7	84,23
**-E-S-**	705,7	4,2	78,64
**-E-T-**	719,7	4,09	60,05
**-E-V-**	717,7	3,62	85,61
**-E-W-**	804,8	3,42	84,14
**-E-Y-**	781,8	3,67	86,8
**-F-A-**	707,7	3,48	84,4
**-F-D-**	751,7	3,84	78,4
**-F-E-**	765,8	3,52	81,42
**-F-F-**	783,8	3,5	91,68
**-F-G-**	693,7	3,6	85,77
**-F-H-**	887,8	3,99	92,24
**-F-I-**	749,8	2,87	90,82
**-F-K-**	878,9	2,71	94,94
**-F-L-**	749,8	3,67	91,53
**-F-M-**	767,8	5,08	64,54
**-F-N-**	750,8	4,09	89,37
**-F-P-**	733,8	5,66	88,11

**-F-Q-**	764,8	4,2	92,08
**-F-R-**	906,9	4,71	92,95
**-F-S-**	723,7	3,85	79,4
**-F-T-**	737,8	3,87	71,48
**-F-V-**	735,8	3,52	89,73
**-F-W-**	822,9	2,81	90,13
**-F-Y-**	799,8	3,82	88,69
**-G-A-**	617,6	4,25	70,84
**-G-D-**	661,6	2,01	73,12
**-G-E-**	675,6	4,26	77,87
**-G-F-**	693,7	3,58	86,59
**-G-G-**	603,6	4,68	77,14
**-G-H-**	797,7	4,59	91,23
**-G-I-**	659,7	4,22	81,53
**-G-K-**	788,7	4,3	87,8
**-G-L-**	659,7	4,05	79,02
**-G-M-**	677,7	3,62	83,97
**-G-N-**	660,6	3,78	75,56
**-G-P-**	643,6	4,95	73,18
**-G-Q-**	674,7	4,01	78,7
**-G-R-**	816,7	5,13	90,47
**-G-S-**	633,6	4	73,09
**-G-T-**	647,6	4	65,08
**-G-V-**	645,7	4,36	82,23
**-G-W-**	732,7	3,56	82,51
**-G-Y-**	709,7	3,62	84,71
**-H-A-**	811,7	3,91	78,59
**-H-D-**	855,7	3,44	81,11
**-H-E-**	869,8	3,58	79,88
**-H-F-**	887,8	3,64	80,65
**-H-G-**	797,7	4,42	90,52
**-H-H-**	991,8	4,37	79
**-H-I-**	853,8	3,73	75,12
**-H-K-**	982,8	4,37	92,82
**-H-L-**	853,8	4,09	74,8
**-H-M-**	871,8	3,88	75,21
**-H-N-**	854,7	2,32	89,05
**-H-P-**	837,8	4,91	87,33

**-H-Q-**	868,8	3,72	78,35
**-H-R-**	1010,9	3,76	88,25
**-H-S-**	827,7	3,96	86,1
**-H-T-**	841,7	4,3	69,73
**-H-V-**	839,8	3,68	74,61
**-H-W-**	926,9	3,55	95,24
**-H-Y-**	903,8	5,36	81,5
**-I-A-**	673,7	3,07	86,61
**-I-D-**	717,7	2,8	84,75
**-I-E-**	731,8	2,81	86,72
**-I-F-**	749,8	2,8	89,07
**-I-G-**	659,7	2,95	88,03
**-I-H-**	853,8	4,1	82,51
**-I-I-**	715,8	3,26	88,93
**-I-K-**	844,8	3,88	86,6
**-I-L-**	715,8	3,76	90,32
**-I-M-**	733,8	3,51	86,07
**-I-N-**	716,7	3,91	90,85
**-I-P-**	699,8	5,92	90,15
**-I-Q-**	730,8	2,78	93,8
**-I-R-**	872,8	4,67	88
**-I-S-**	689,7	3,71	74,78
**-I-T-**	703,7	3,64	69,65
**-I-V-**	701,8	3,7	83,16
**-I-W-**	788,8	3,64	94,74
**-I-Y-**	765,8	4,51	90,27
**-K-A-**	802,8	4,28	80,86
**-K-D-**	846,8	3,71	78,73
**-K-E-**	860,8	3,85	82,59
**-K-F-**	878,9	3,83	88,26
**-K-G-**	788,7	2,92	90,97
**-K-H-**	982,8	5,8	72,52
**-K-I-**	844,8	4,71	70,86
**-K-K-**	973,9	4,36	71,02
**-K-L-**	844,8	4,09	77,76
**-K-M-**	862,9	4,26	80,04
**-K-N-**	845,8	3,66	86,05
**-K-P-**	828,8	5,47	87,88

**-K-Q-**	859,8	3,82	72,08
**-K-R-**	1001,9	4,88	92,62
**-K-S-**	818,8	4,26	77,89
**-K-T-**	832,8	4,42	67,93
**-K-V-**	830,8	4,27	78,89
**-K-W-**	917,9	3,94	92,37
**-K-Y-**	894,8	4,94	80,15
**-L-A-**	673,7	3,5	83,84
**-L-D-**	717,7	2,88	81,35
**-L-E-**	731,8	3,13	83,06
**-L-F-**	749,8	3,18	90,96
**-L-G-**	659,7	2,89	85,81
**-L-H-**	853,8	3,99	88,72
**-L-I-**	715,8	2,43	89,18
**-L-K-**	844,8	4,53	91,5
**-L-L-**	715,8	3,68	90,84
**-L-M-**	733,8	4,22	93,15
**-L-N-**	716,7	3,72	90,08
**-L-P-**	699,8	5,72	84,81
**-L-Q-**	730,8	4,27	86,23
**-L-R-**	872,8	4,35	97,96
**-L-S-**	689,7	3,92	79,88
**-L-T-**	703,7	3,83	68,89
**-L-V-**	701,8	3,73	89,12
**-L-W-**	788,8	3,79	89,71
**-L-Y-**	765,8	4,28	90,09
**-M-A-**	691,7	5,1	82,44
**-M-D-**	735,8	4,41	81,43
**-M-E-**	749,8	5,03	82,97
**-M-F-**	767,8	5,2	93,01
**-M-G-**	677,7	4,75	86,65
**-M-H-**	871,8	6,19	82,79
**-M-I-**	733,8	4,54	87,84
**-M-K-**	862,9	5,63	81,13
**-M-L-**	733,8	4,89	93,31
**-M-M-**	751,9	4,89	93,47
**-M-N-**	734,8	5,36	84,64
**-M-P-**	717,8	7,4	91,19

**-M-Q-**	748,8	5,5	89,12
**-M-R-**	890,9	5,83	87,92
**-M-S-**	707,7	5,27	82,9
**-M-T-**	721,8	4	82,37
**-M-V-**	719,8	4,98	93,6
**-M-W-**	806,9	5,8	92,02
**-M-Y-**	783,8	5,37	90,83
**-N-A-**	674,7	4,52	86,48
**-N-D-**	718,7	4,05	79,63
**-N-E-**	732,7	4,68	78,38
**-N-F-**	750,8	4,35	89,05
**-N-G-**	660,6	4,31	83,79
**-N-H-**	854,7	4,55	86,88
**-N-I-**	716,7	3,48	90,2
**-N-K-**	845,8	4,73	90,04
**-N-L-**	716,7	4,08	87,74
**-N-M-**	734,8	2,99	89,55
**-N-N-**	717,7	4,62	85,4
**-N-P-**	700,7	6,77	76,28
**-N-Q-**	731,7	3,85	84,19
**-N-R-**	873,8	4,5	92,08
**-N-S-**	690,7	4,18	75,32
**-N-T-**	704,7	3,88	75,91
**-N-V-**	702,7	3,79	81,89
**-N-W-**	789,8	3,96	95,44
**-N-Y-**	766,8	5,91	89,4
**-P-A-**	657,7	6,02	78,8
**-P-D-**	701,7	5,24	81,17
**-P-E-**	715,7	4,03	88,79
**-P-F-**	733,8	5,6	86,49
**-P-G-**	643,6	6,26	76,3
**-P-H-**	837,8	6,91	89,64
**-P-I-**	699,8	6,1	82,37
**-P-K-**	828,8	7,16	90,54
**-P-L-**	699,8	7,12	86,27
**-P-M-**	717,8	7,35	90,77
**-P-N-**	700,7	7,53	75,45
**-P-P-**	683,7	10,26	86,91

**-P-Q-**	714,7	7,55	82,29
**-P-R-**	856,8	7,83	84,03
**-P-S-**	673,7	6,29	79,84
**-P-T-**	687,7	5,66	72,63
**-P-V-**	685,7	6,73	86,99
**-P-W-**	772,8	6,12	87,58
**-P-Y-**	749,8	7,15	82,52
**-Q-A-**	688,7	4,98	82,9
**-Q-D-**	732,7	4,57	82,33
**-Q-E-**	746,7	5,05	83,37
**-Q-F-**	764,8	3,77	94,1
**-Q-G-**	674,7	4,89	82,84
**-Q-H-**	868,8	5,29	89,04
**-Q-I-**	730,8	3,53	90,88
**-Q-K-**	859,8	5,53	89,9
**-Q-L-**	730,8	4,18	90,32
**-Q-M-**	748,8	4,53	81,36
**-Q-N-**	731,7	4,81	76,93
**-Q-P-**	714,7	7,12	77,48
**-Q-Q-**	745,7	4,57	86,06
**-Q-R-**	887,8	5,13	87,33
**-Q-S-**	704,7	4,95	65,7
**-Q-T-**	718,7	4,43	64,81
**-Q-V-**	716,7	5,01	79,8
**-Q-W-**	803,8	5,1	84,43
**-Q-Y-**	780,8	5,59	92,08
**-R-A-**	830,8	4,68	80,64
**-R-D-**	874,8	4,69	79,45
**-R-E-**	888,8	5,13	85,63
**-R-F-**	906,9	5,06	94,55
**-R-G-**	816,7	4,9	88,99
**-R-H-**	1010,9	5,41	88,86
**-R-I-**	872,8	4,91	80,33
**-R-K-**	1001,9	5,67	91,22
**-R-L-**	872,8	4,96	85,47
**-R-M-**	890,9	5,76	78,09
**-R-N-**	873,8	4,61	88,18
**-R-P-**	856,8	7,32	88,99

**-R-Q-**	887,8	5,06	86,63
**-R-R-**	1029,9	4,79	90,32
**-R-S-**	846,8	4,66	78,66
**-R-T-**	860,8	2,89	64,36
**-R-V-**	858,8	6,06	77,32
**-R-W-**	945,9	4	95,36
**-R-Y-**	922,9	6,75	81,78
**-S-A-**	647,6	5,76	72,21
**-S-D-**	691,6	4,82	79,87
**-S-E-**	705,7	5,2	67,62
**-S-F-**	723,7	3,56	86,97
**-S-G-**	633,6	4,59	72,67
**-S-H-**	827,7	6,39	85,22
**-S-I-**	689,7	5,45	87,98
**-S-K-**	818,8	5,51	89,64
**-S-L-**	689,7	4,78	82,04
**-S-M-**	707,7	4,86	84,2
**-S-N-**	690,7	5,61	77,69
**-S-P-**	673,7	6,97	75,39
**-S-Q-**	704,7	4,96	83,83
**-S-R-**	846,8	5,49	90,05
**-S-S-**	663,6	5,23	68,81
**-S-T-**	677,7	5,22	73,36
**-S-V-**	675,7	4,71	85,5
**-S-W-**	762,8	5,07	86,09
**-S-Y-**	739,7	5,17	83,97
**-T-A-**	661,7	5,01	59,32
**-T-D-**	705,7	4,17	50,94
**-T-E-**	719,7	4,52	67,27
**-T-F-**	737,8	4,1	81,05
**-T-G-**	647,6	5,13	64,11
**-T-H-**	841,7	4,68	80,65
**-T-I-**	703,7	4,32	85,54
**-T-K-**	832,8	4,49	79,63
**-T-L-**	703,7	3,89	74,6
**-T-M-**	721,8	4,13	71,53
**-T-N-**	704,7	4,31	80,26
**-T-P-**	687,7	6,87	75,02

**-T-Q-**	718,7	4,6	71,45
**-T-R-**	860,8	4,19	72,16
**-T-S-**	677,7	4,64	68,37
**-T-T-**	691,7	4,98	46,91
**-T-V-**	689,7	2,88	85,62
**-T-W-**	776,8	4,35	82,47
**-T-Y-**	753,8	5,17	84,17
**-V-A-**	659,7	2,85	90,41
**-V-D-**	703,7	3,28	87,67
**-V-E-**	717,7	3,55	90,66
**-V-F-**	735,8	3,82	88,71
**-V-G-**	645,7	3,53	90,96
**-V-H-**	839,8	5,06	75,27
**-V-I-**	701,8	4,24	86,62
**-V-K-**	830,8	4,86	78,76
**-V-L-**	701,8	4,25	87,39
**-V-M-**	719,8	4,18	93,06
**-V-N-**	702,7	4,39	92,49
**-V-P-**	685,7	5,42	96,44
**-V-Q-**	716,7	4,83	91,28
**-V-R-**	858,8	5,89	79,07
**-V-S-**	675,7	4,39	83,96
**-V-T-**	689,7	4,07	70,91
**-V-V-**	687,7	4,44	85,77
**-V-W-**	774,8	4,53	90,72
**-V-Y-**	751,8	5,23	85,78
**-W-A-**	746,8	5,08	85,03
**-W-D-**	790,8	3,32	87,35
**-W-E-**	804,8	4,76	89,93
**-W-F-**	822,9	4,91	92,74
**-W-G-**	732,7	4,74	89,21
**-W-H-**	926,9	6,46	92,53
**-W-I-**	788,8	4,63	87,76
**-W-K-**	917,9	5,41	92,76
**-W-L-**	788,8	4,74	92,21
**-W-M-**	806,9	4,48	94,82
**-W-N-**	789,8	5,44	92,14
**-W-P-**	772,8	6,79	90,35

**-W-Q-**	803,8	4,32	89,13
**-W-R-**	945,9	5,12	93,29
**-W-S-**	762,8	4,5	83,01
**-W-T-**	776,8	4,43	73
**-W-V-**	774,8	4,66	91,42
**-W-W-**	861,9	4,63	89,36
**-W-Y-**	838,9	4,9	92,79
**-Y-A-**	723,7	5,24	80,73
**-Y-D-**	767,7	4,87	85,57
**-Y-E-**	781,8	4,6	89,56
**-Y-F-**	799,8	4,87	90,17
**-Y-G-**	709,7	4,94	86,25
**-Y-H-**	903,8	5,99	87,26
**-Y-I-**	765,8	4,28	86,36
**-Y-K-**	894,8	5,66	86,25
**-Y-L-**	765,8	4,61	89,15
**-Y-M-**	783,8	4,76	90,94
**-Y-N-**	766,8	4,68	88,7
**-Y-P-**	749,8	6	89,28
**-Y-Q-**	780,8	4,73	91
**-Y-R-**	922,9	5,27	93,13
**-Y-S-**	739,7	2,1	85,13
**-Y-T-**	753,8	4,35	81,68
**-Y-V-**	751,8	4,14	89,79
**-Y-W-**	838,9	4,89	90,08
**-Y-Y-**	815,8	5,17	88,34

7. Luminescent assay validation library

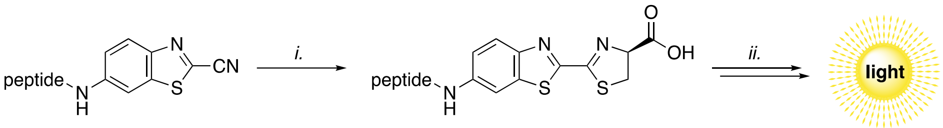

Figure S3. i. β-A-X-R/β-A-X-K substrate (1 equiv) D-cysteine (1.5 equiv), buffer (25 mM HEPES, 125 mM NaCl, 0.5% BSA, pH = 7.4), 37 °C, 30 min. ii. Protease (thrombin/FXa, final conc 10 nM), ATP (1.5 equiv), $MgCl_2$ (10 equiv), Quantilum® recombinant firefly luciferase (final conc 10 µM).

D-Cysteine (final conc 1 mM) and assay buffer (25 mM HEPES, 125 mM NaCl, 0.5% BSA, pH = 7.4) were added to a well containing methoxyacetyl-βA-X-R-6ABTC or methoxyacetyl-βA-X-K-6ABTC (final conc 667 µM) with a total volume of 30 µL. After incubation for 30 min at 37 °C, 30 µL of the detection mix was added containing luciferase (Quantilum®, Promega, final conc 10 µM), $MgCl_2$ (final conc 6.7 mM), ATP (final conc 1 mM), protease enzyme (Factor Xa or thrombin) (final conc 10 nM), and assay buffer (25 mM HEPES, 125 mM NaCl, 0.5% BSA, pH = 7.4). The luminescence was recorded in relative light units (RLU) every minute for 30 min at 37 °C with an integration time of 1000 ms using a SpectraMax M3 plate reader (Molecular Devices, San Jose, CA, USA). The Area Under the Curve (AUC; RLU) was calculated via:

$$AUC = \sum_{t=0}^{29} \frac{60 * (\gamma_t + \gamma_{t+1})}{2}$$

where *t* is the time in min and *y* the luminescent signal in RLU/s. All measurements were performed in duplicate, of which the mean AUC was calculated and plotted using GraphPad Prism (version 9.0).

8. NMR Spectra

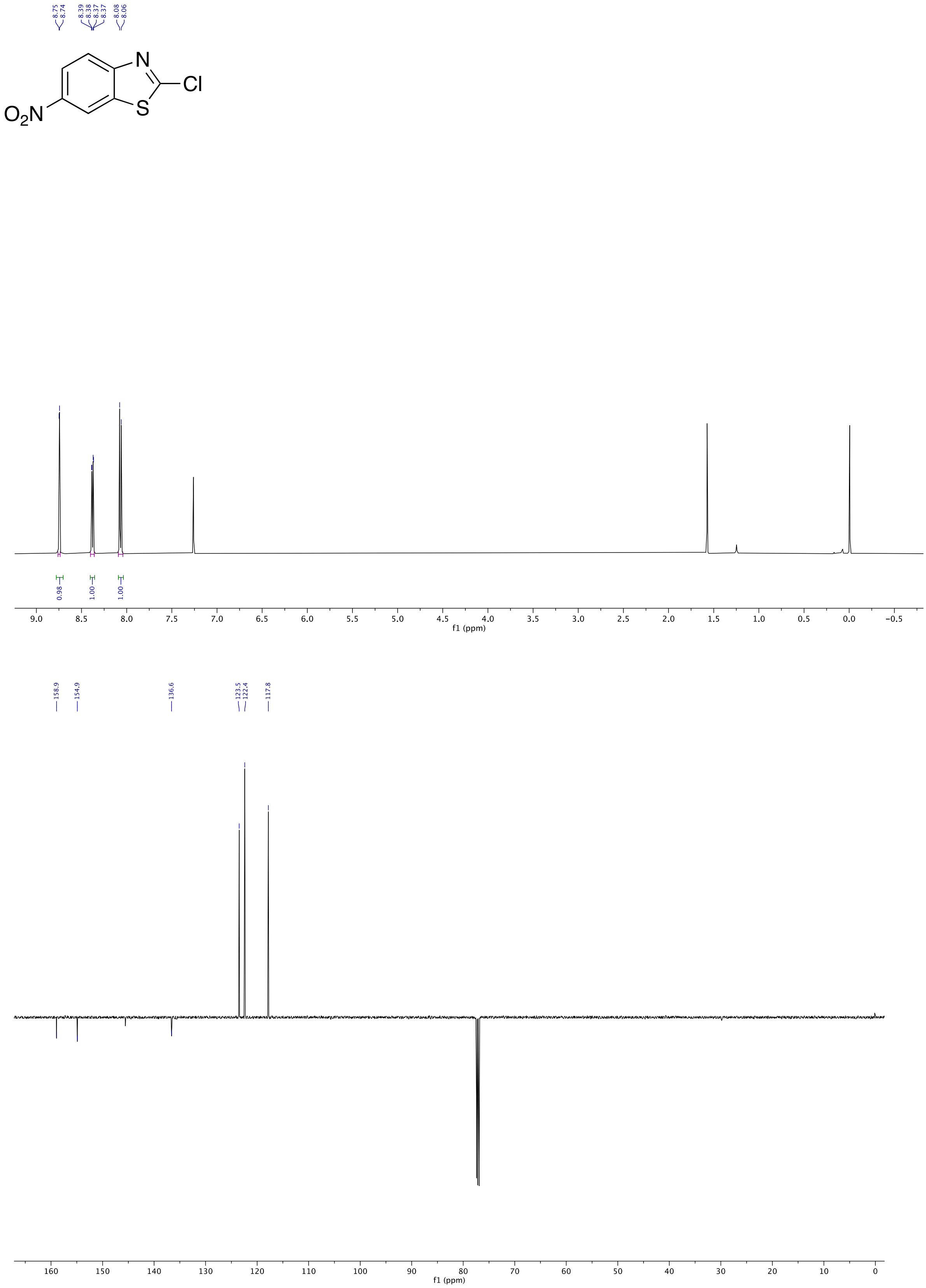

Figure S4: ^{1}H and ^{13}C NMR spectra of compound **8**.

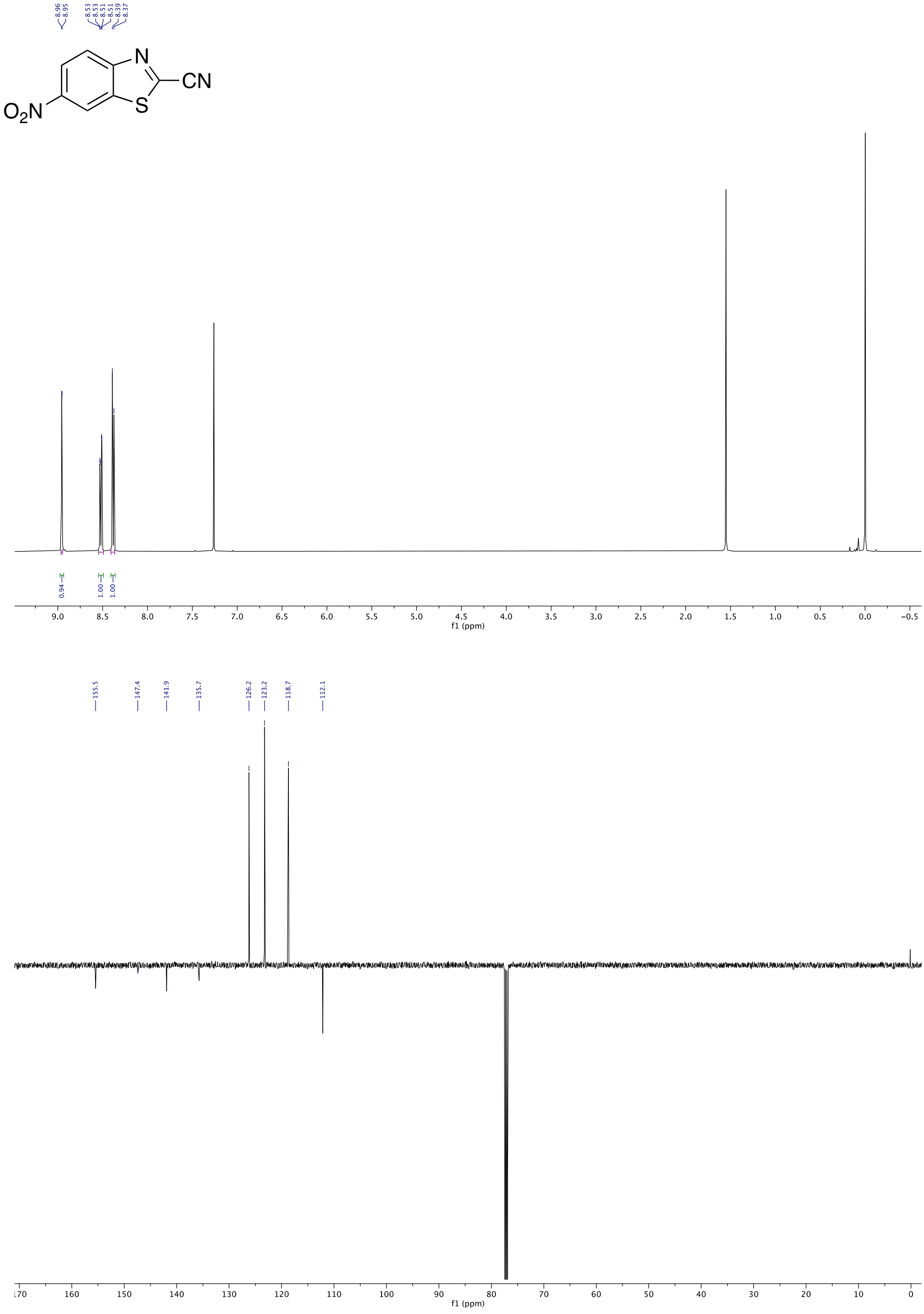

Figure S5: ^{1}H and ^{13}C NMR spectra of compound **9**.

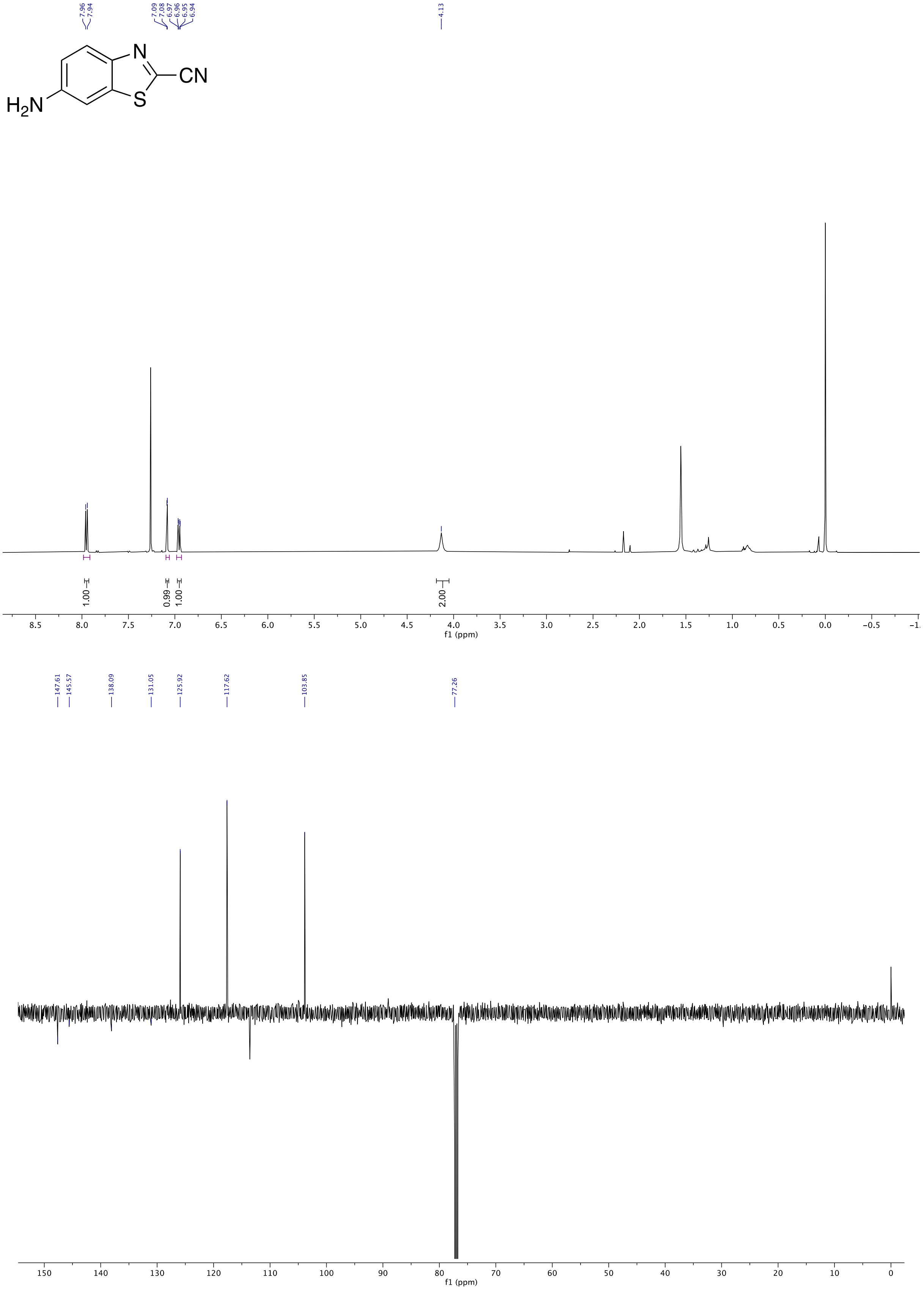

Figure S6: ^{1}H and ^{13}C NMR spectra of compound **1**.

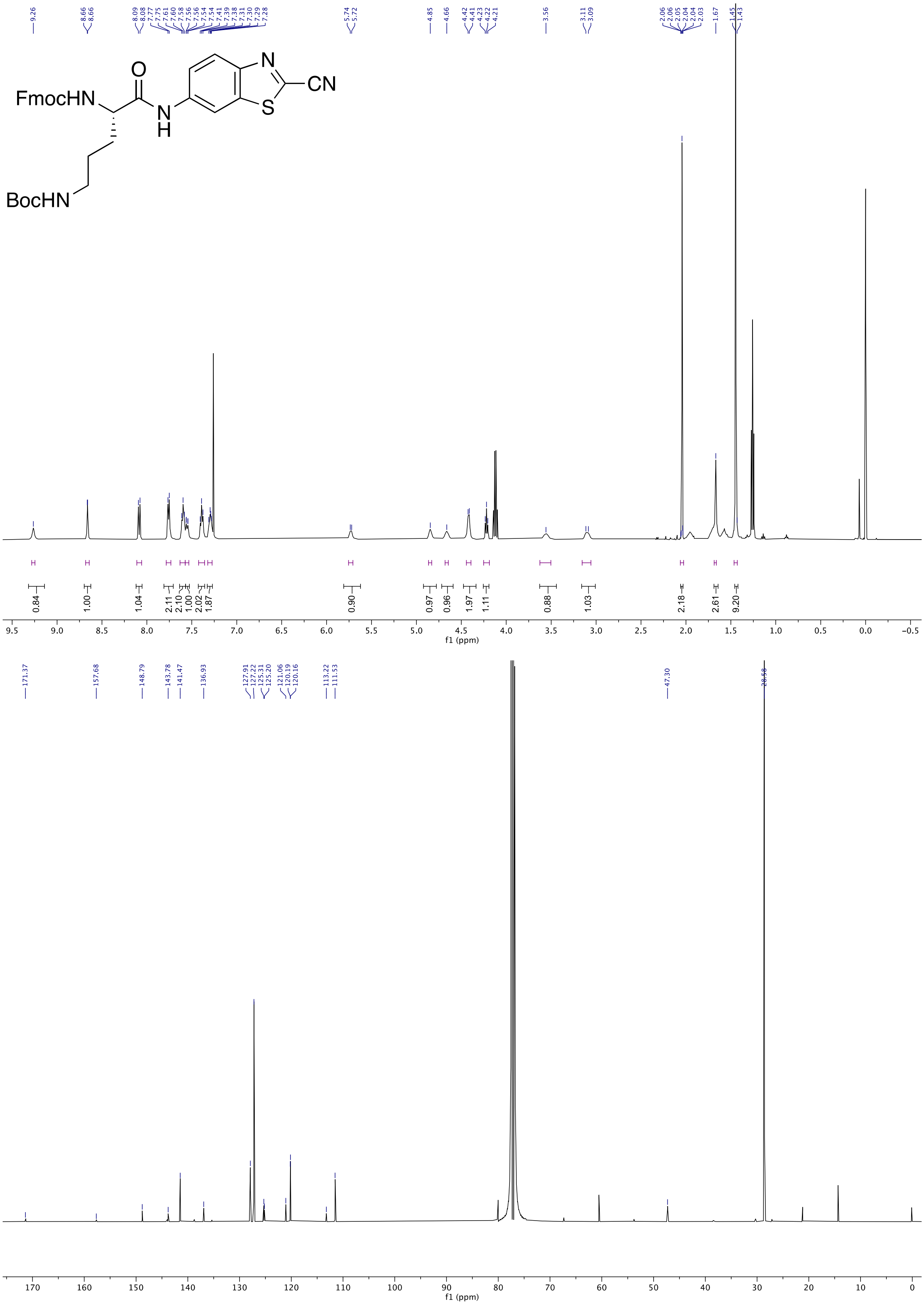

Figure S7: ^{1}H and ^{13}C NMR spectra of compound **4**.

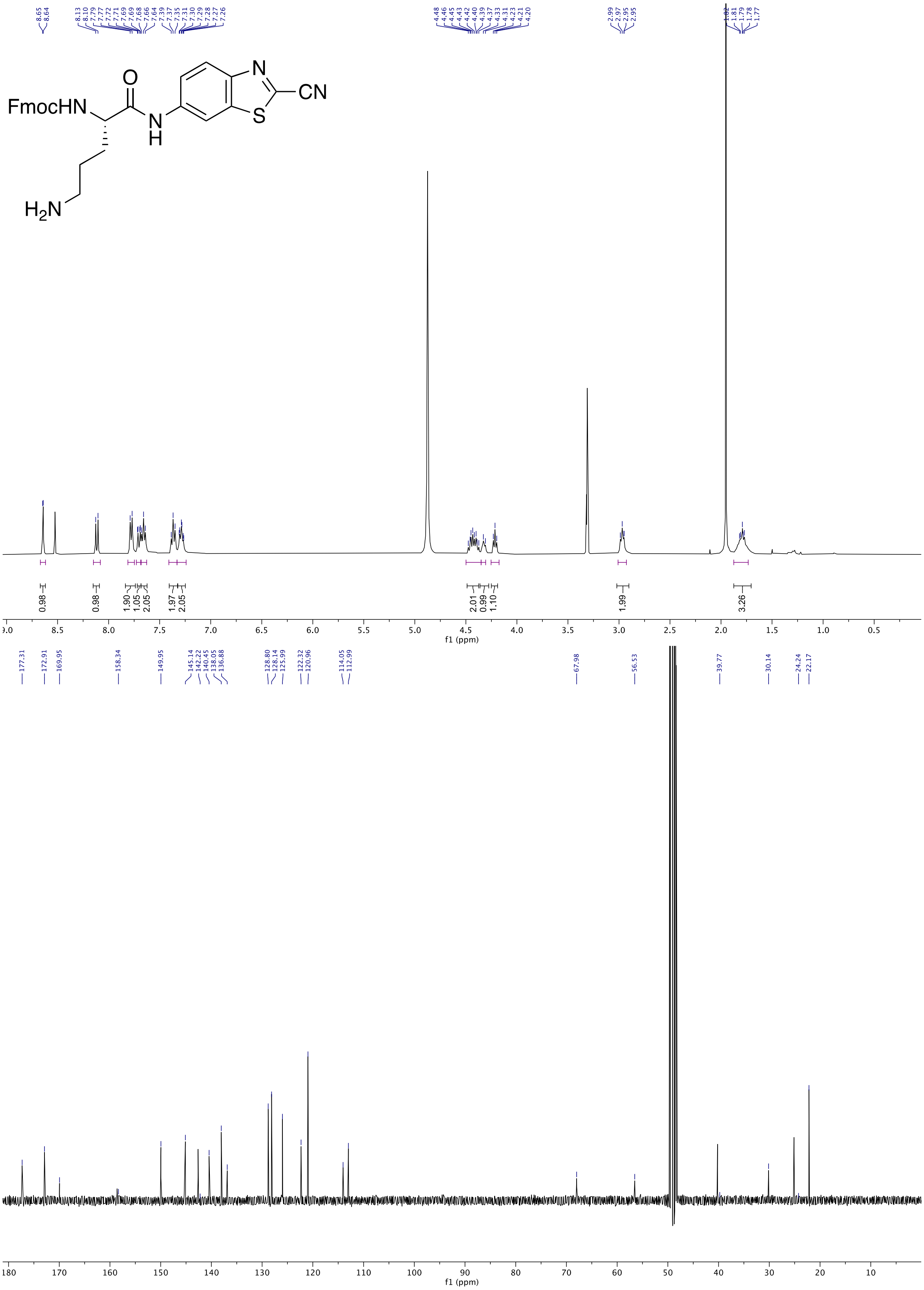

Figure S8: ^{1}H and ^{13}C NMR spectra of compound **6**.

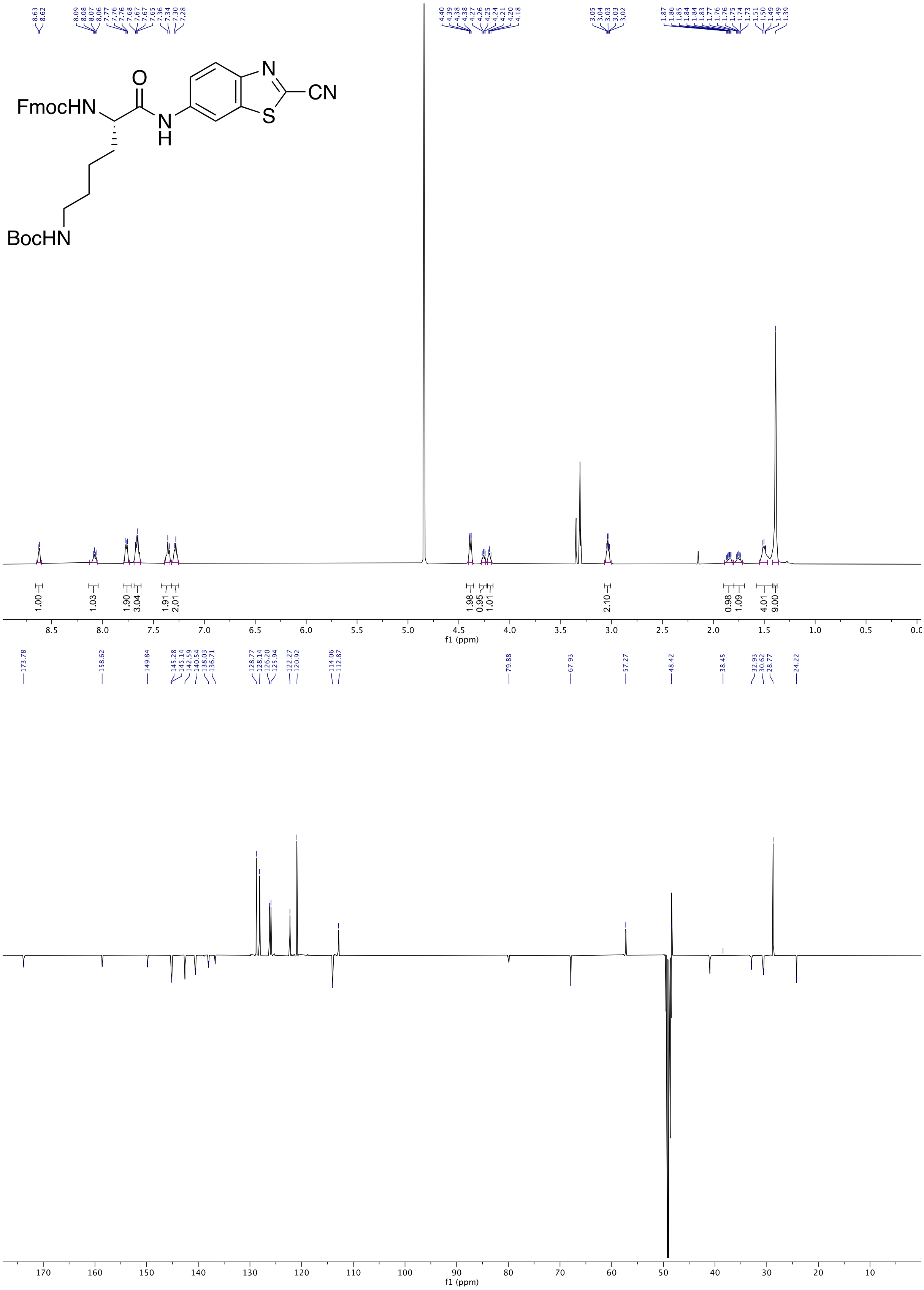

Figure S9: ^{1}H and ^{13}C NMR spectra of compound **5**.

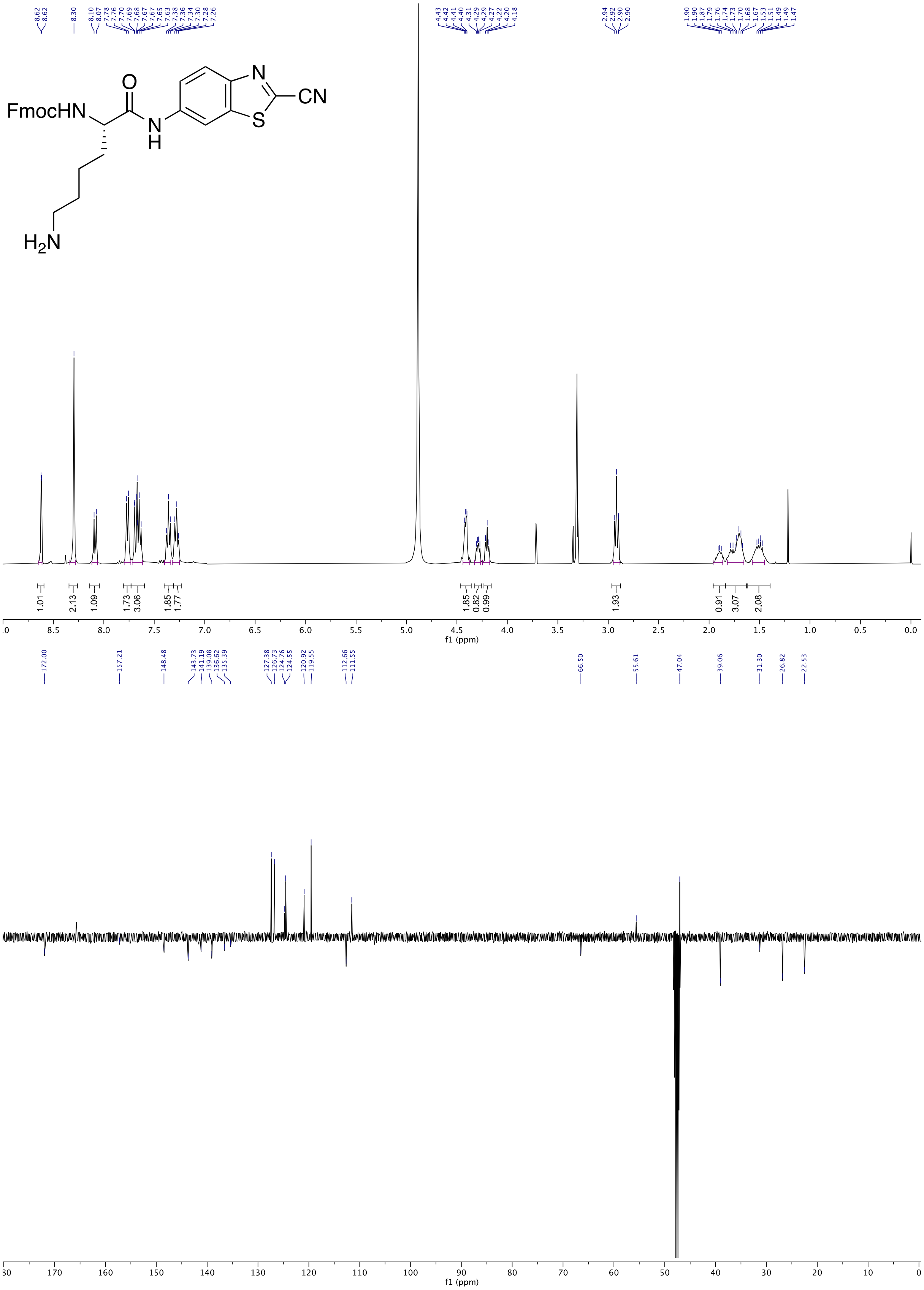

Figure S10: ^{1}H and ^{13}C NMR spectra of compound **7**.

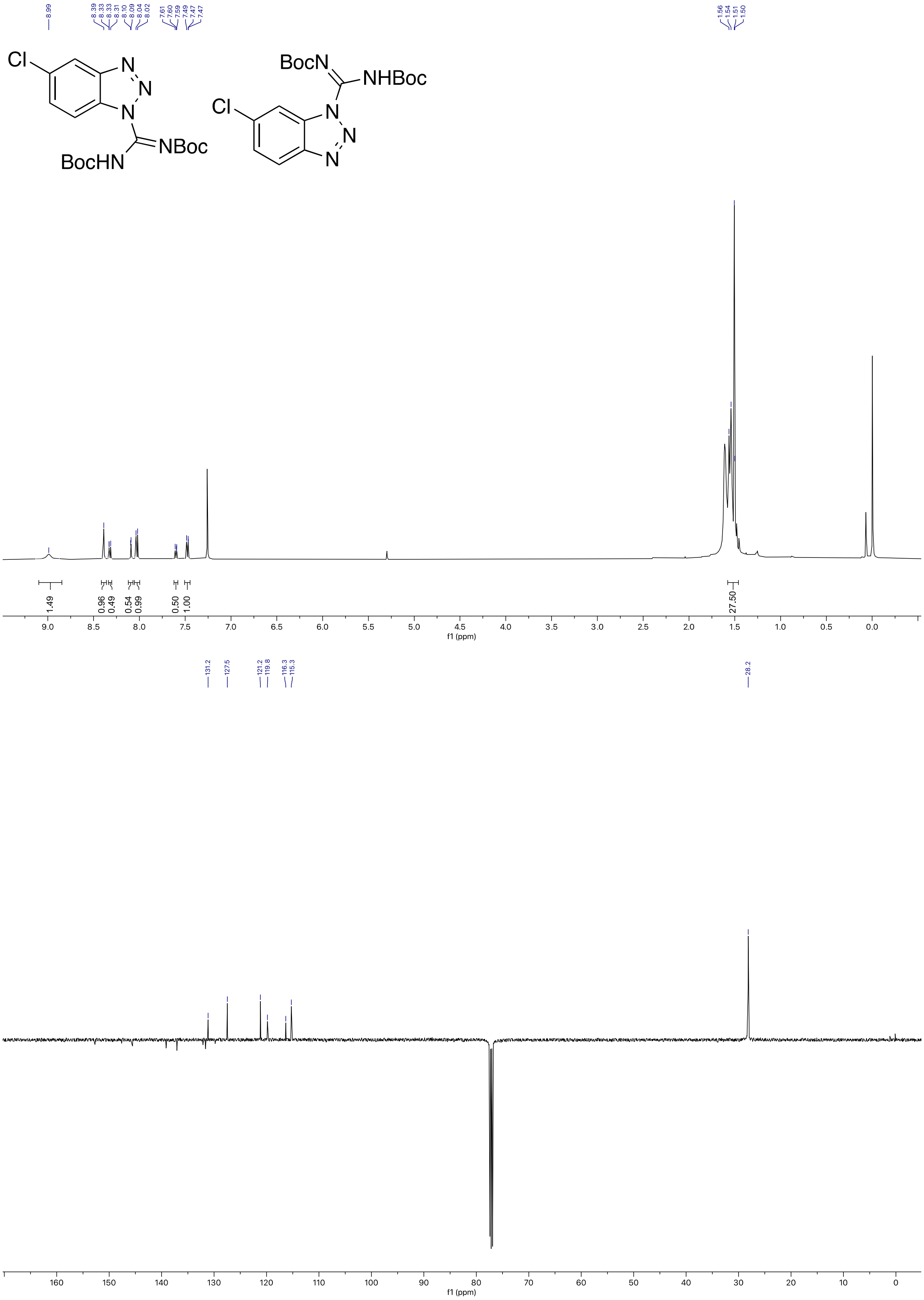

Figure S11: ^{1}H and ^{13}C NMR spectra of compound **9**.

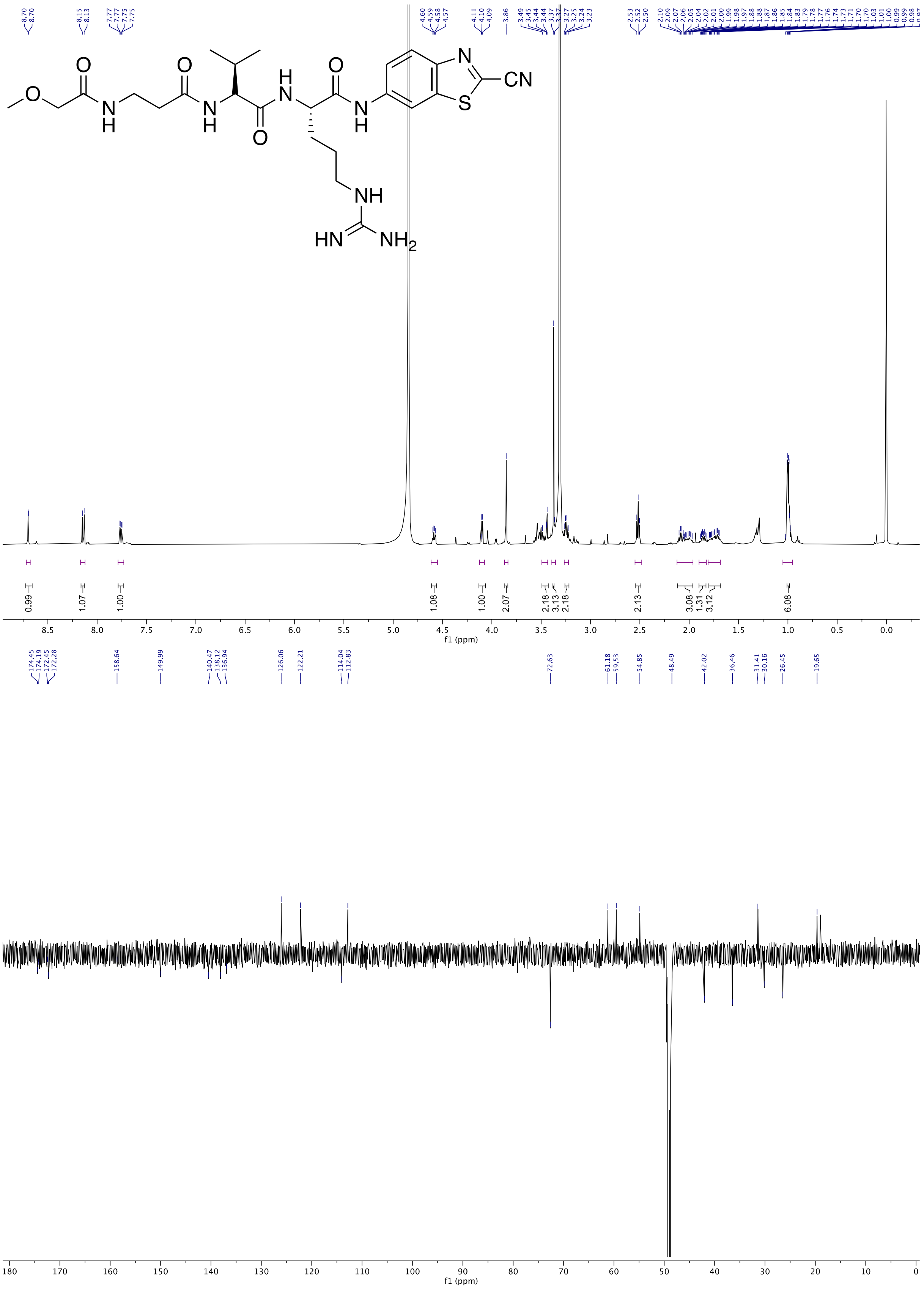

Figure S12: ^{1}H and ^{13}C NMR spectra of methoxyacetyl-βAVR-6ABTC.

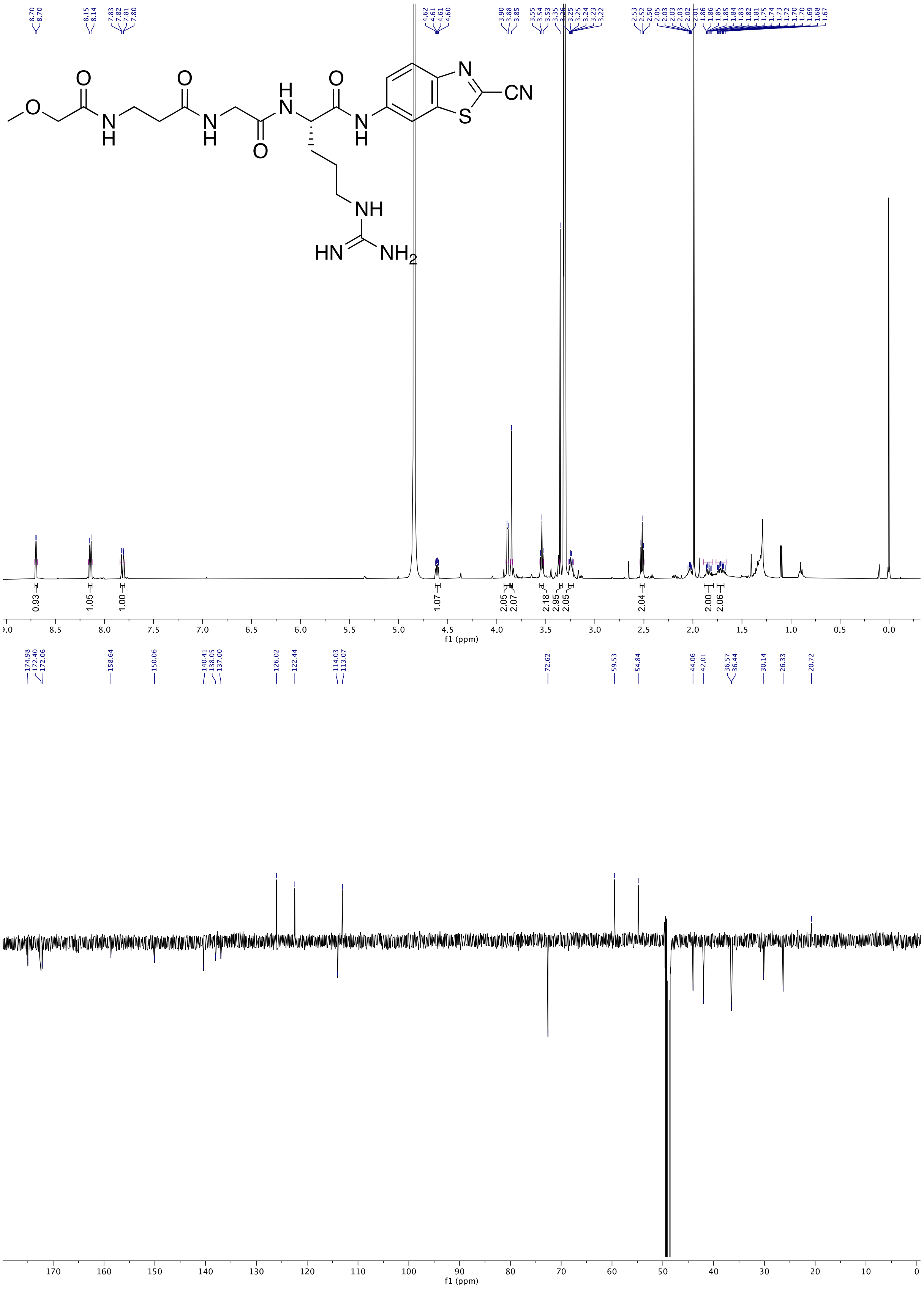

Figure S13: ^{1}H and ^{13}C NMR spectra of methoxyacetyl-βAGR-6ABTC.

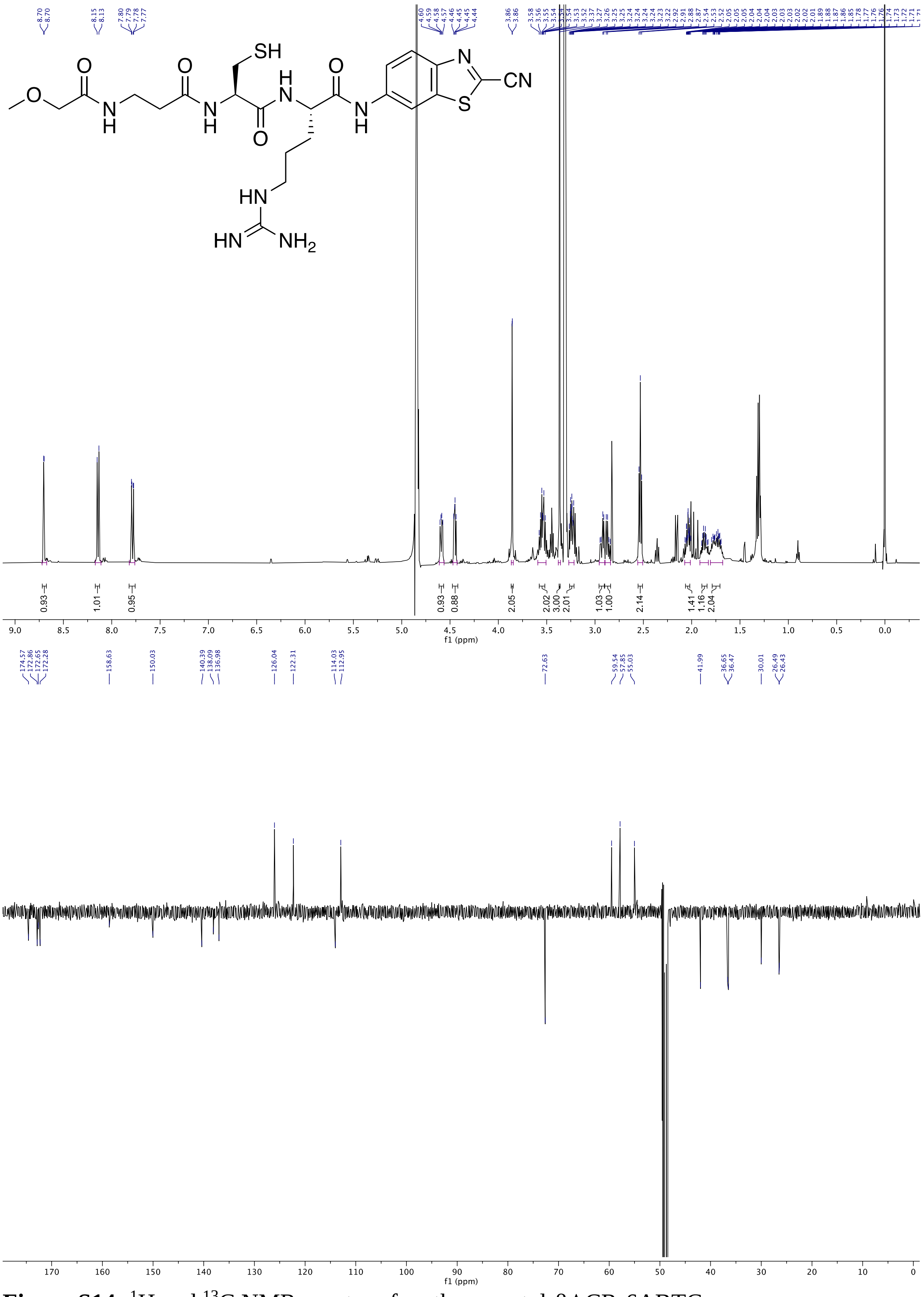

Figure S14: ^{1}H and ^{13}C NMR spectra of methoxyacetyl-βACR-6ABTC.

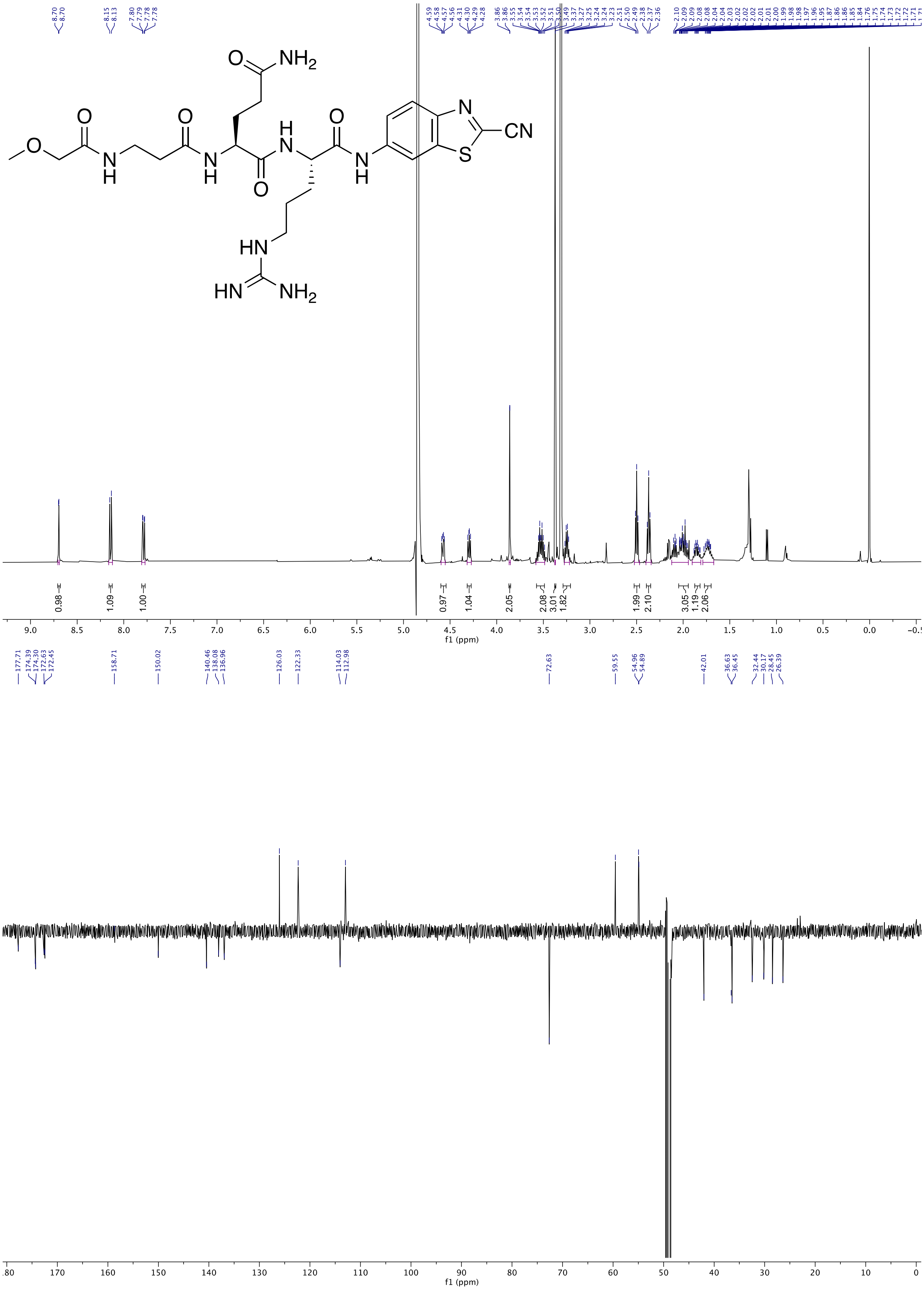

Figure S15: ^{1}H and ^{13}C NMR spectra of methoxyacetyl-βAQR-6ABTC.

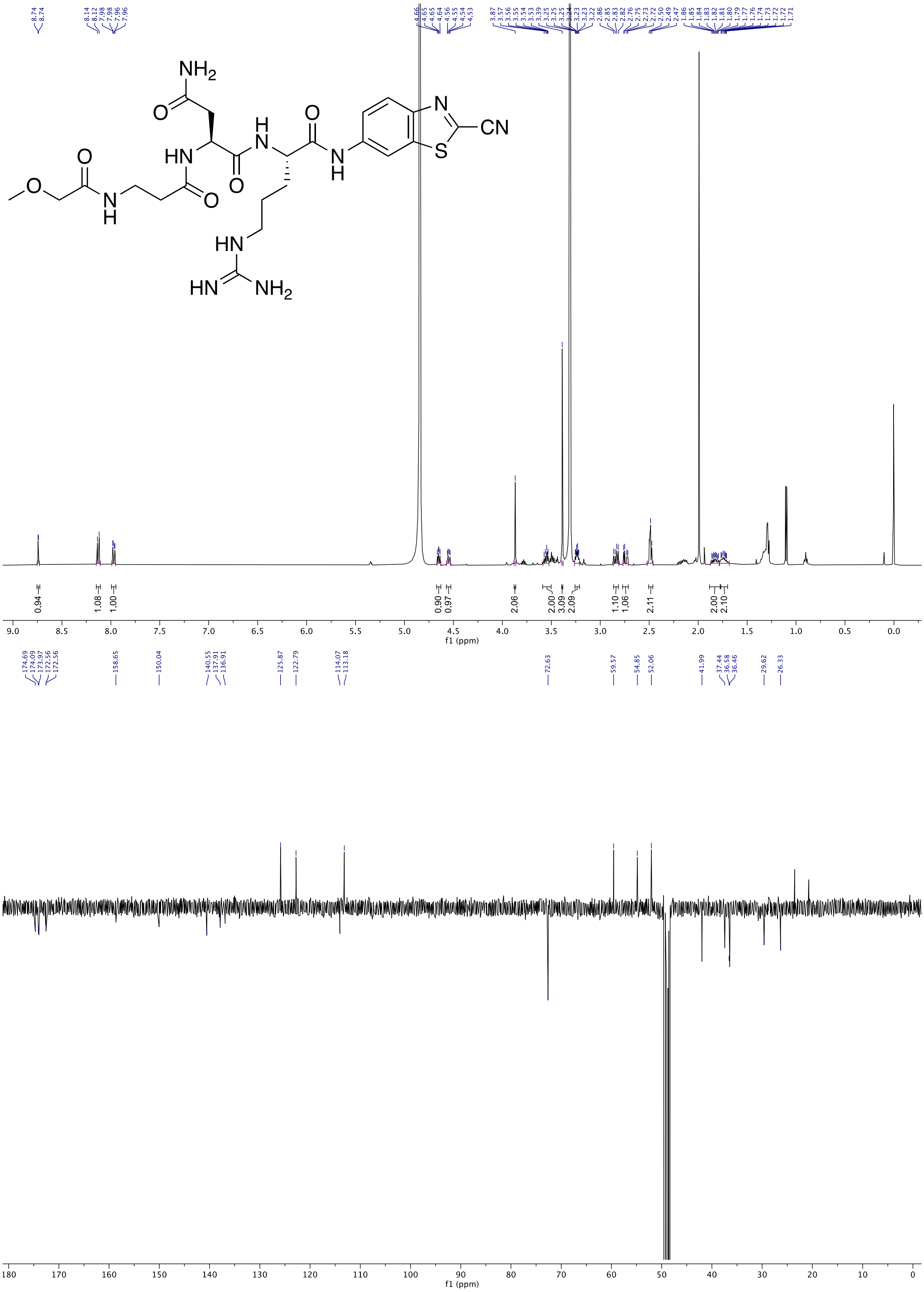

Figure S16: ^{1}H and ^{13}C NMR spectra of methoxyacetyl-βANR-6ABTC.

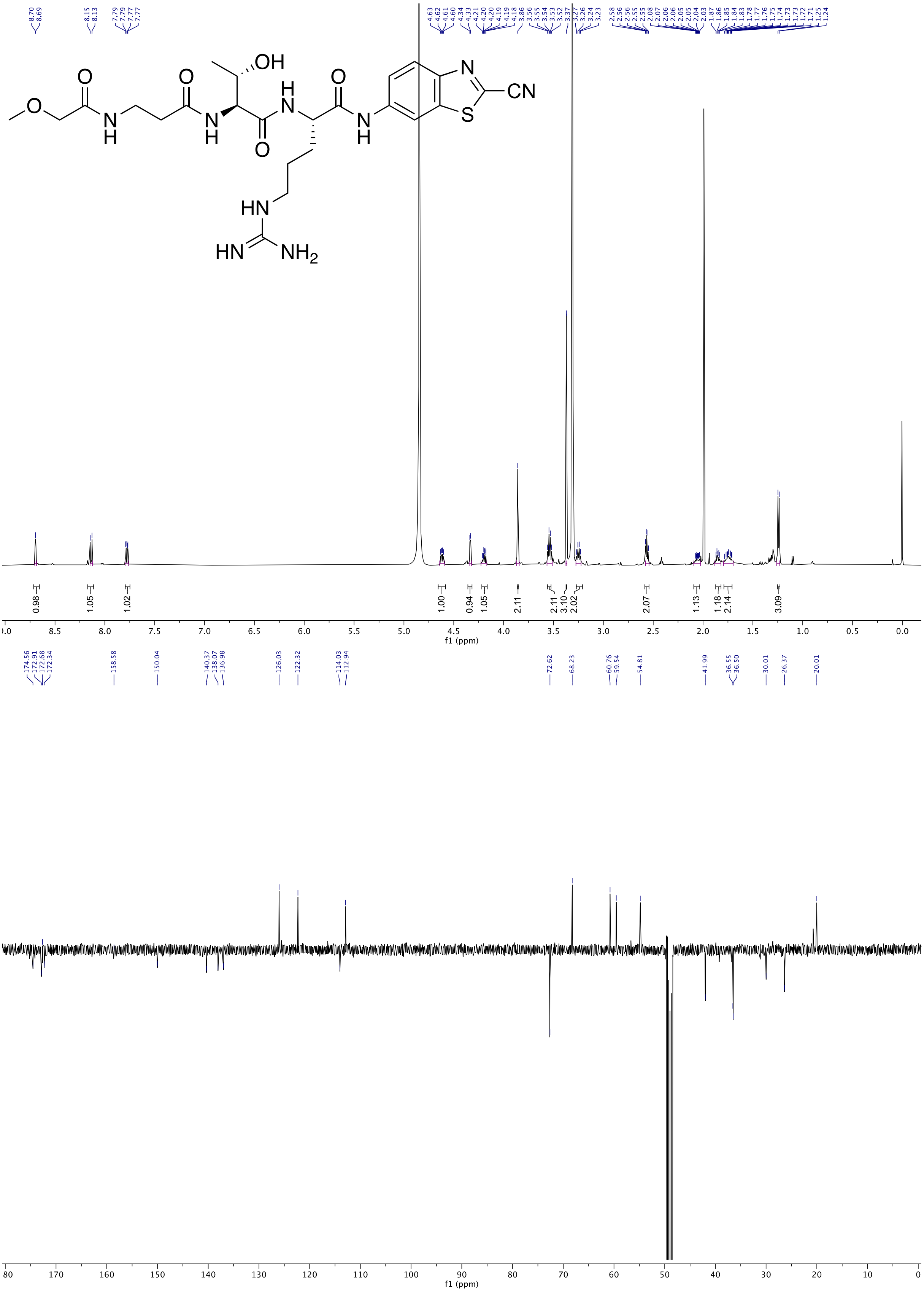

Figure S17: ^{1}H and ^{13}C NMR spectra of methoxyacetyl-βATR-6ABTC.

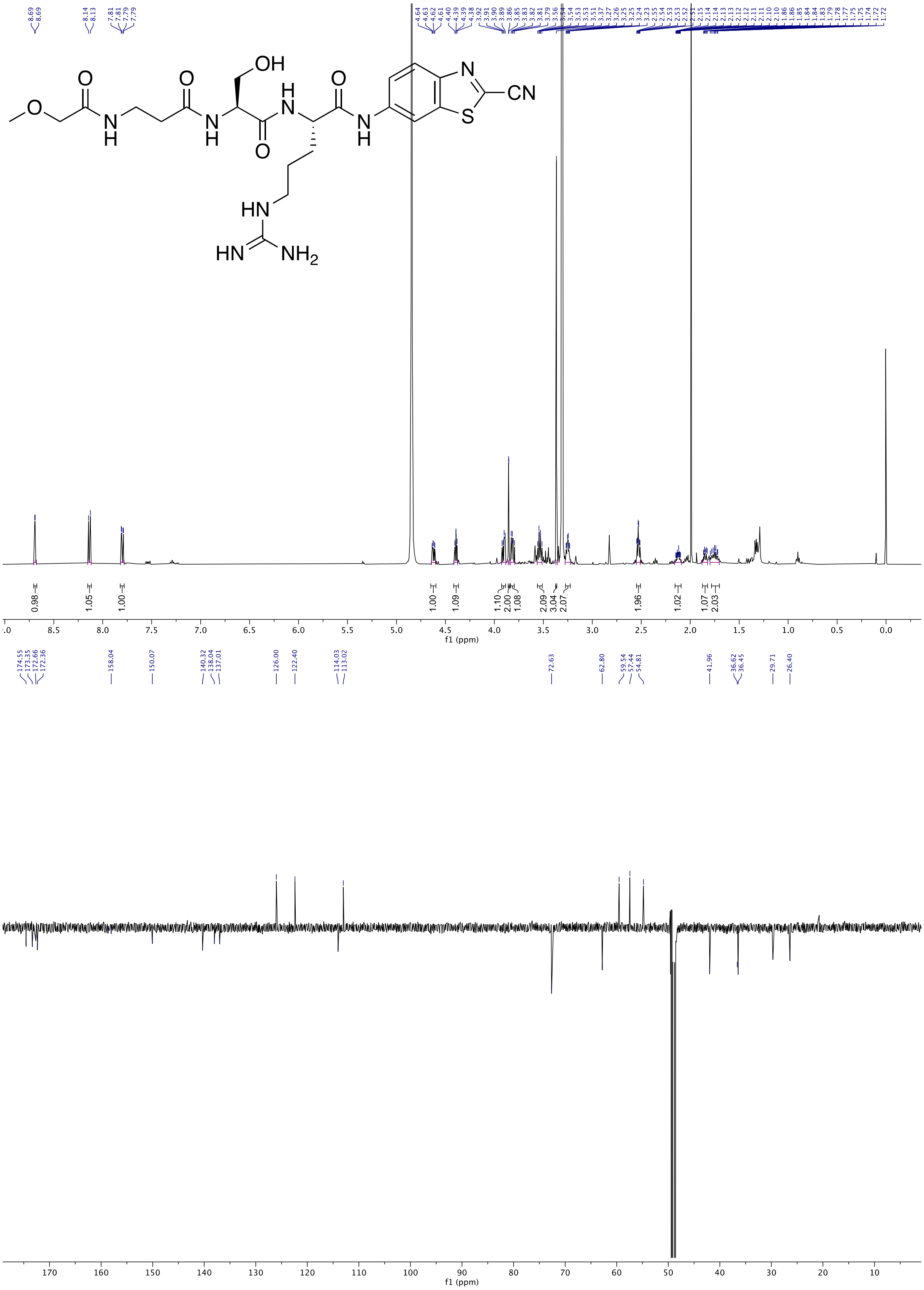

Figure S18: ^{1}H and ^{13}C NMR spectra of methoxyacetyl-βASR-6ABTC.

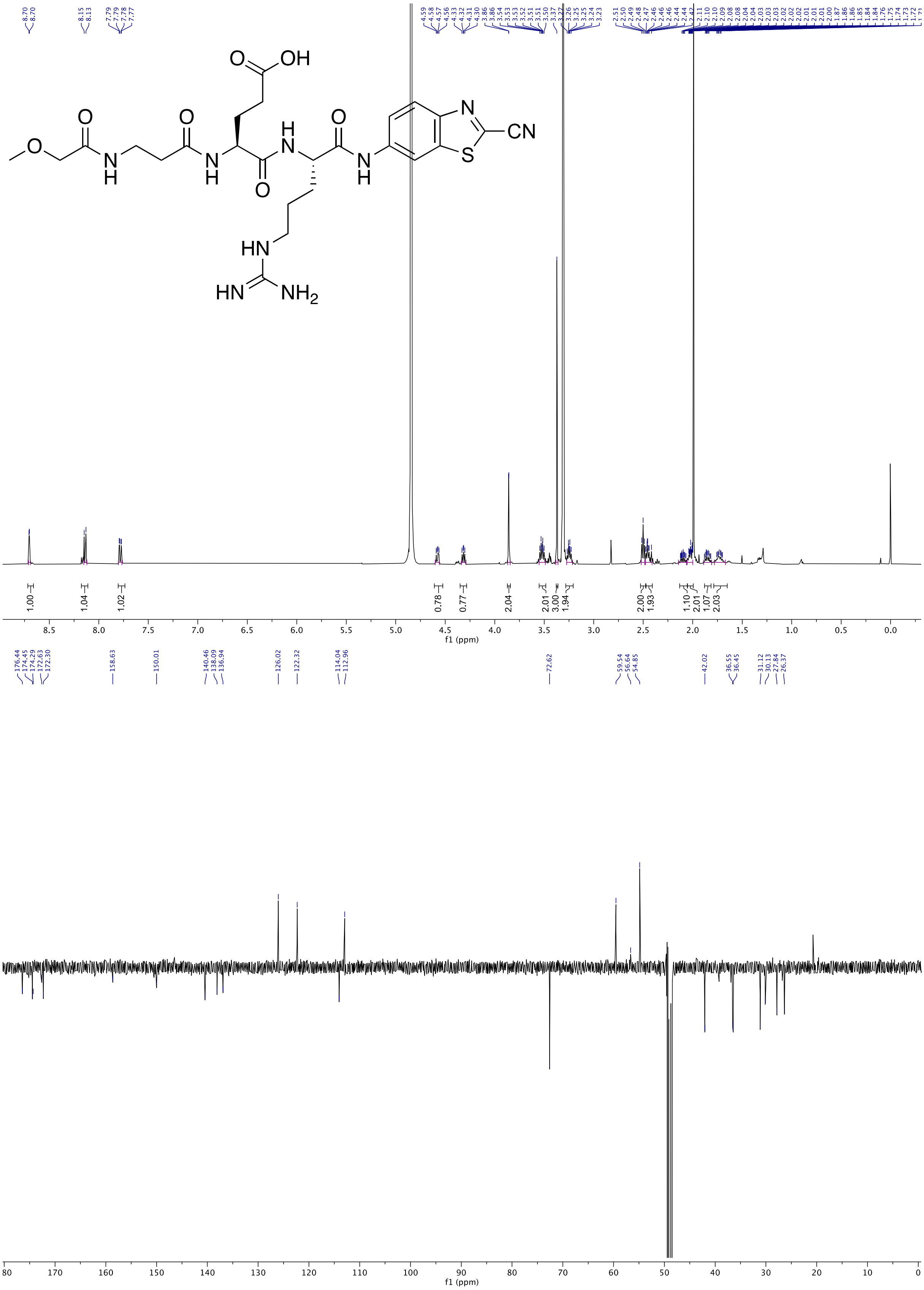

Figure S19: ^{1}H and ^{13}C NMR spectra of methoxyacetyl-βAER-6ABTC.

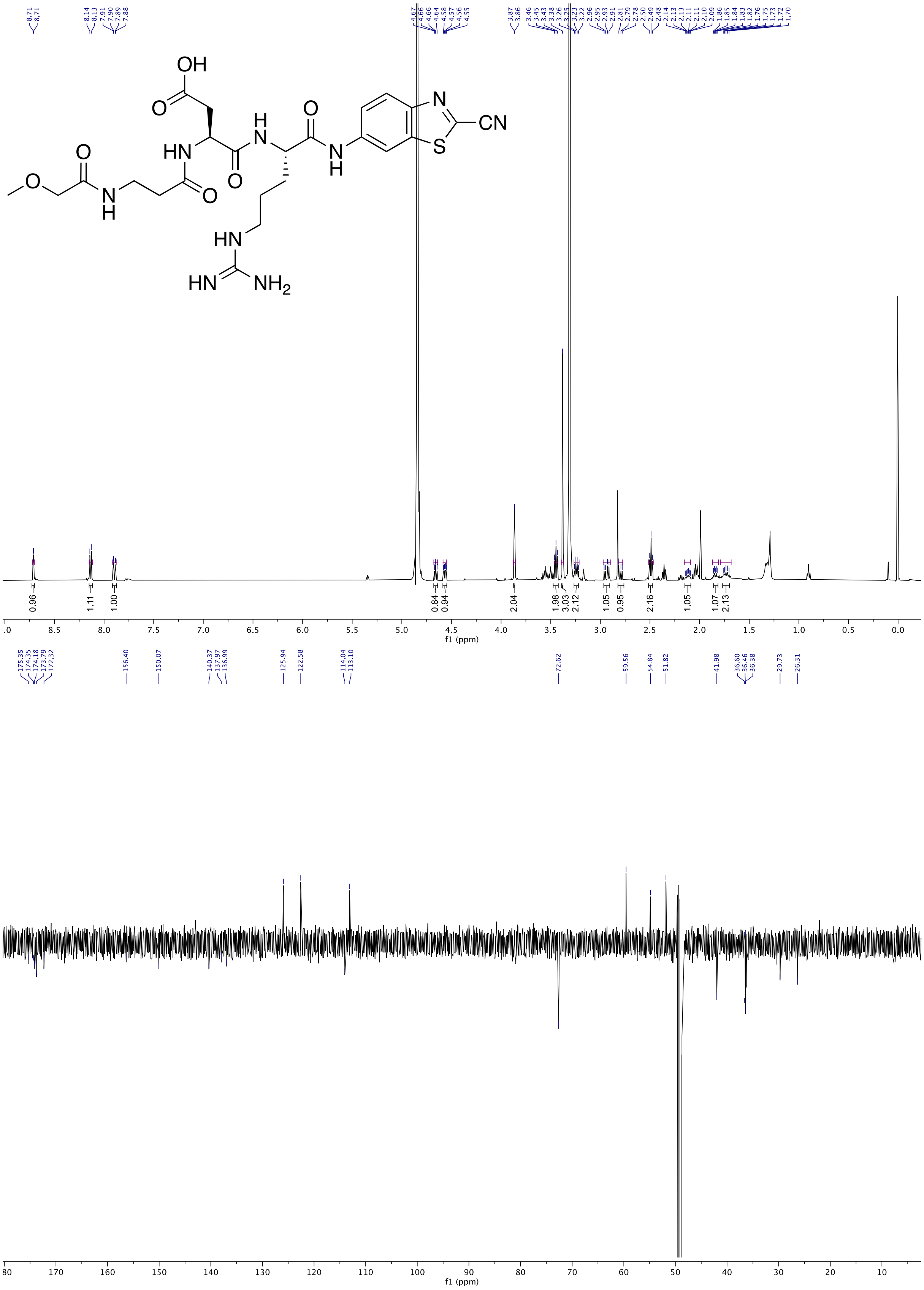

Figure S20: ^{1}H and ^{13}C NMR spectra of methoxyacetyl-βADR-6ABTC.

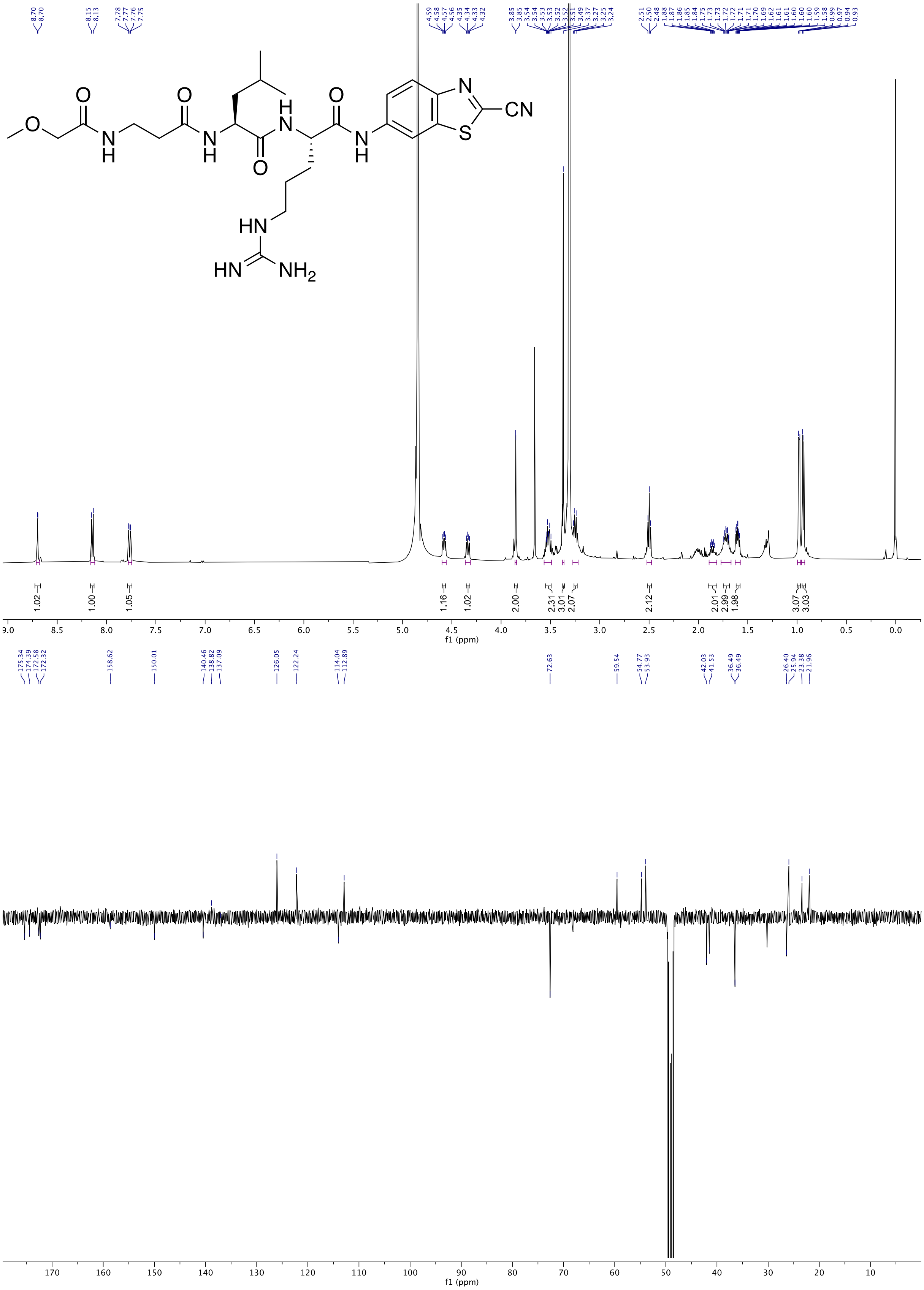

Figure S21: ^{1}H and ^{13}C NMR spectra of methoxyacetyl-βALR-6ABTC.

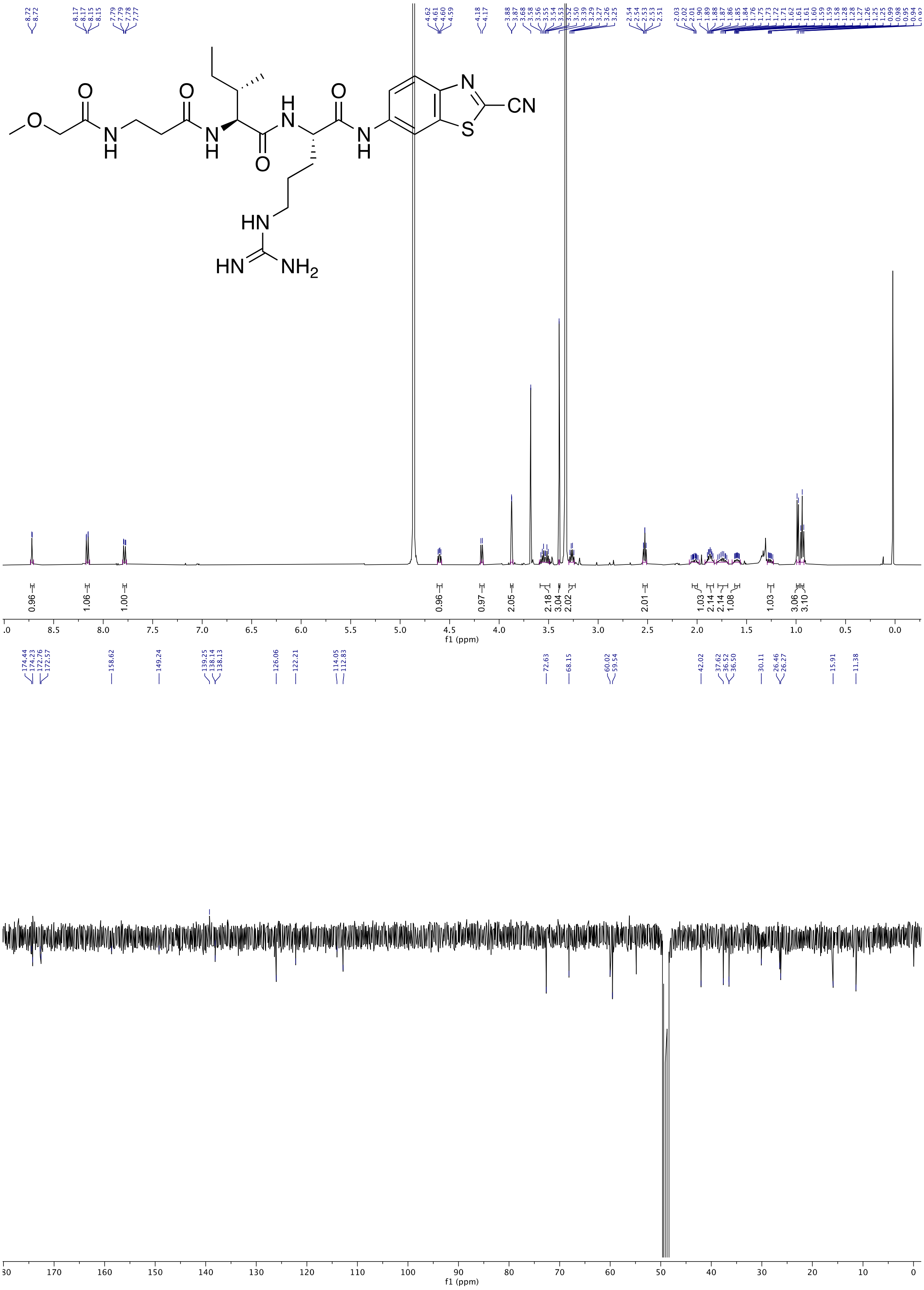

Figure S22: ^{1}H and ^{13}C NMR spectra of methoxyacetyl-βAIR-6ABTC.

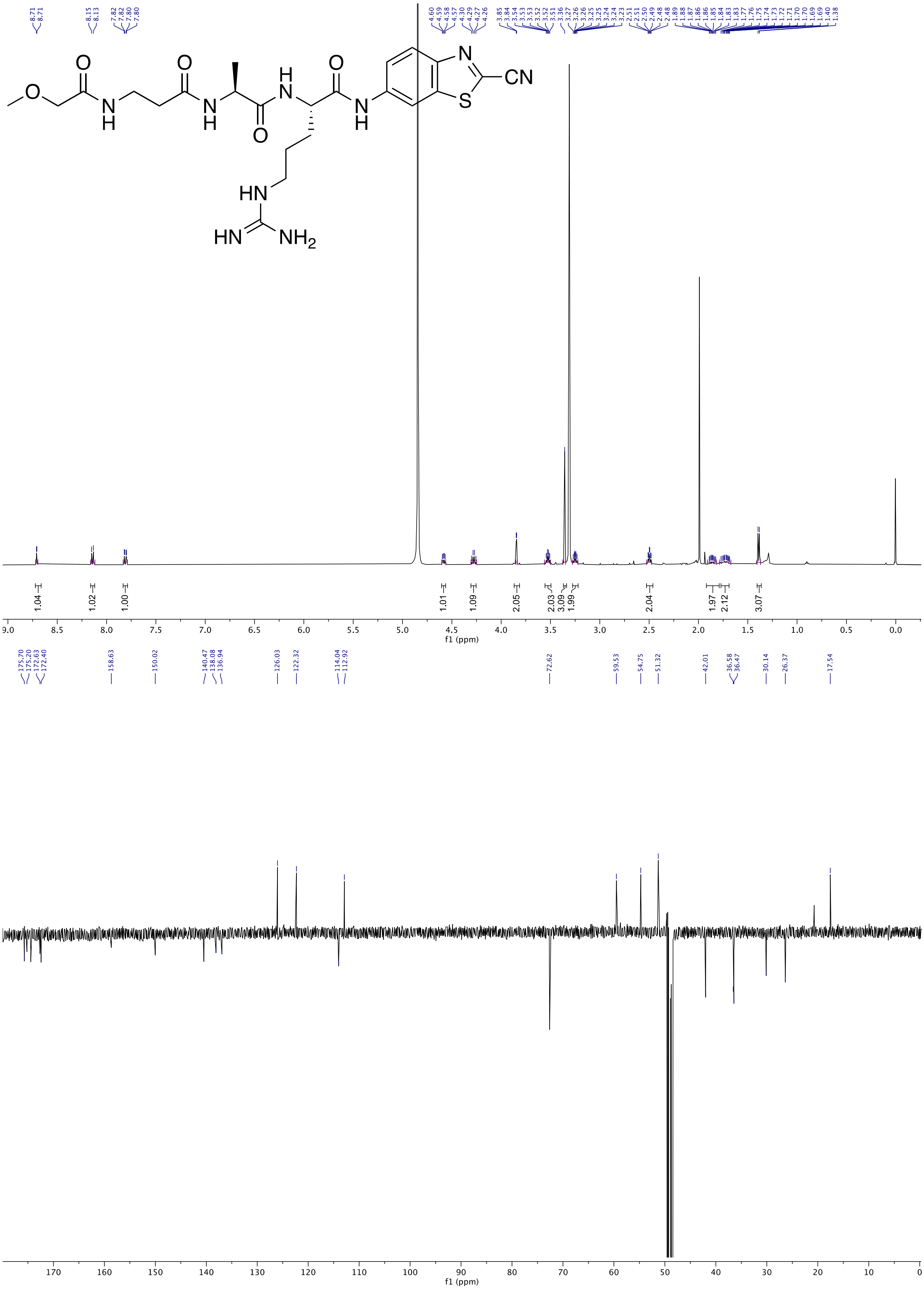

Figure S23: ^{1}H and ^{13}C NMR spectra of methoxyacetyl-βAAR-6ABTC.

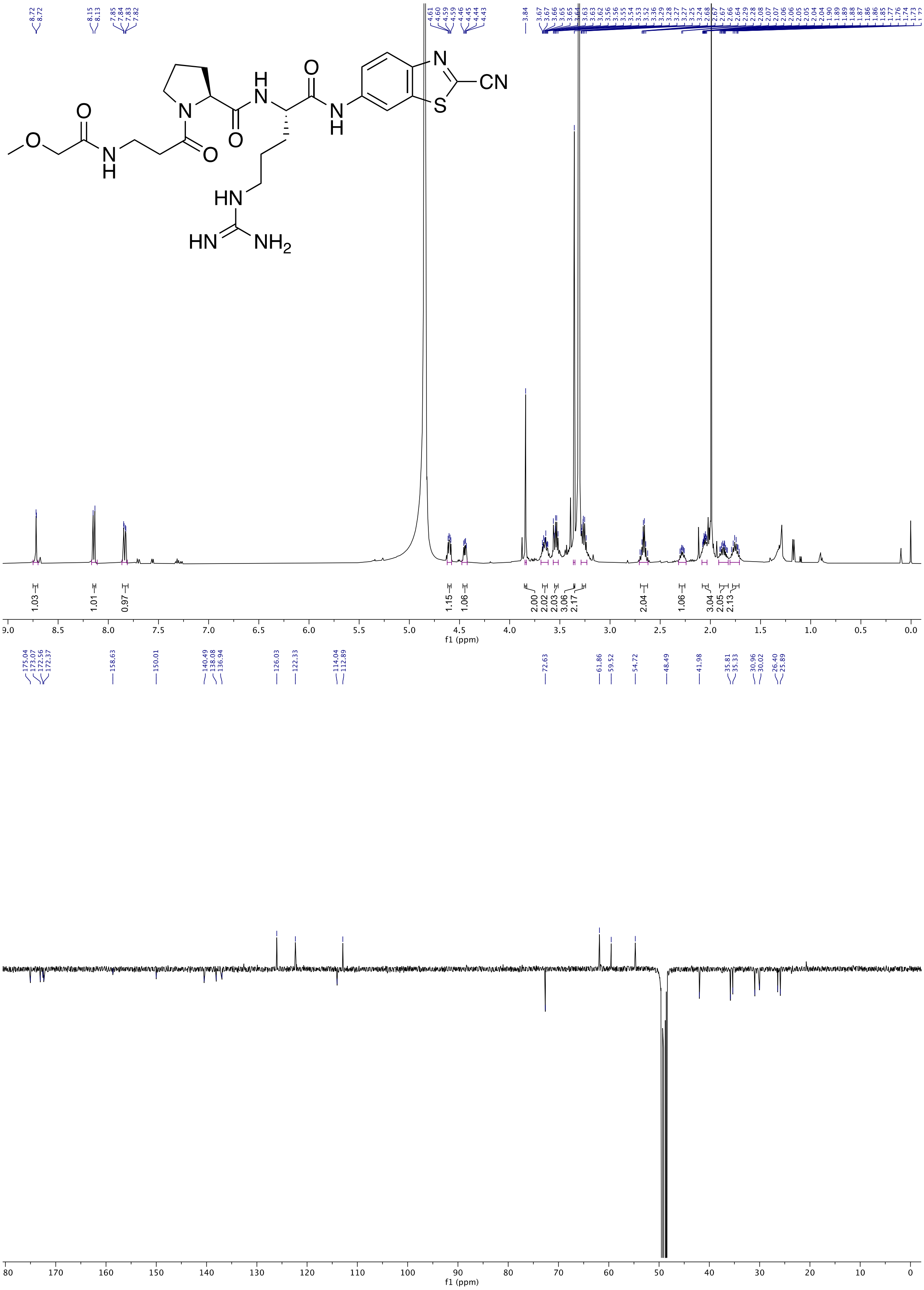

Figure S24: ^{1}H and ^{13}C NMR spectra of methoxyacetyl-βAPR-6ABTC.

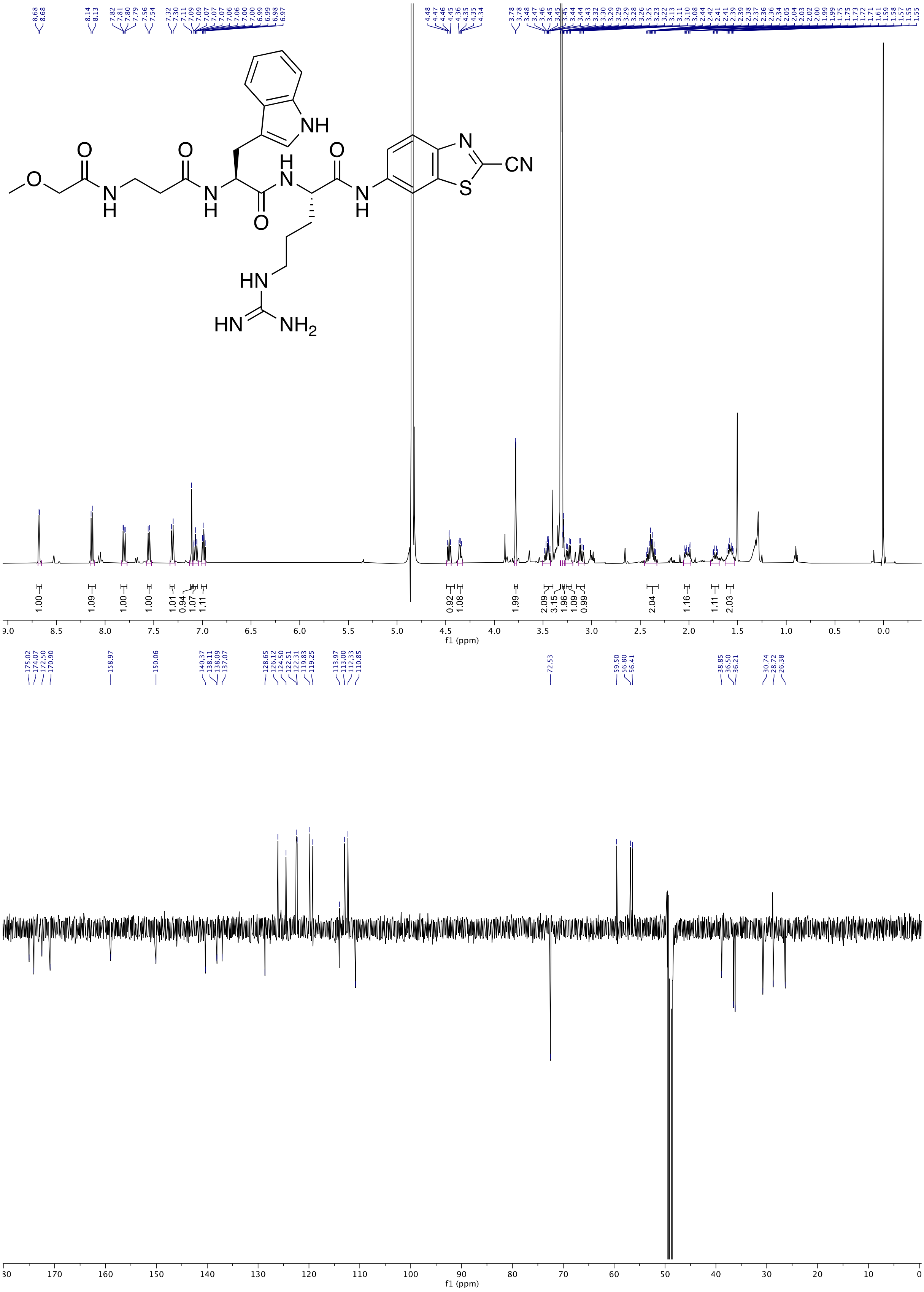

Figure S25: ^{1}H and ^{13}C NMR spectra of methoxyacetyl-βAWR-6ABTC.

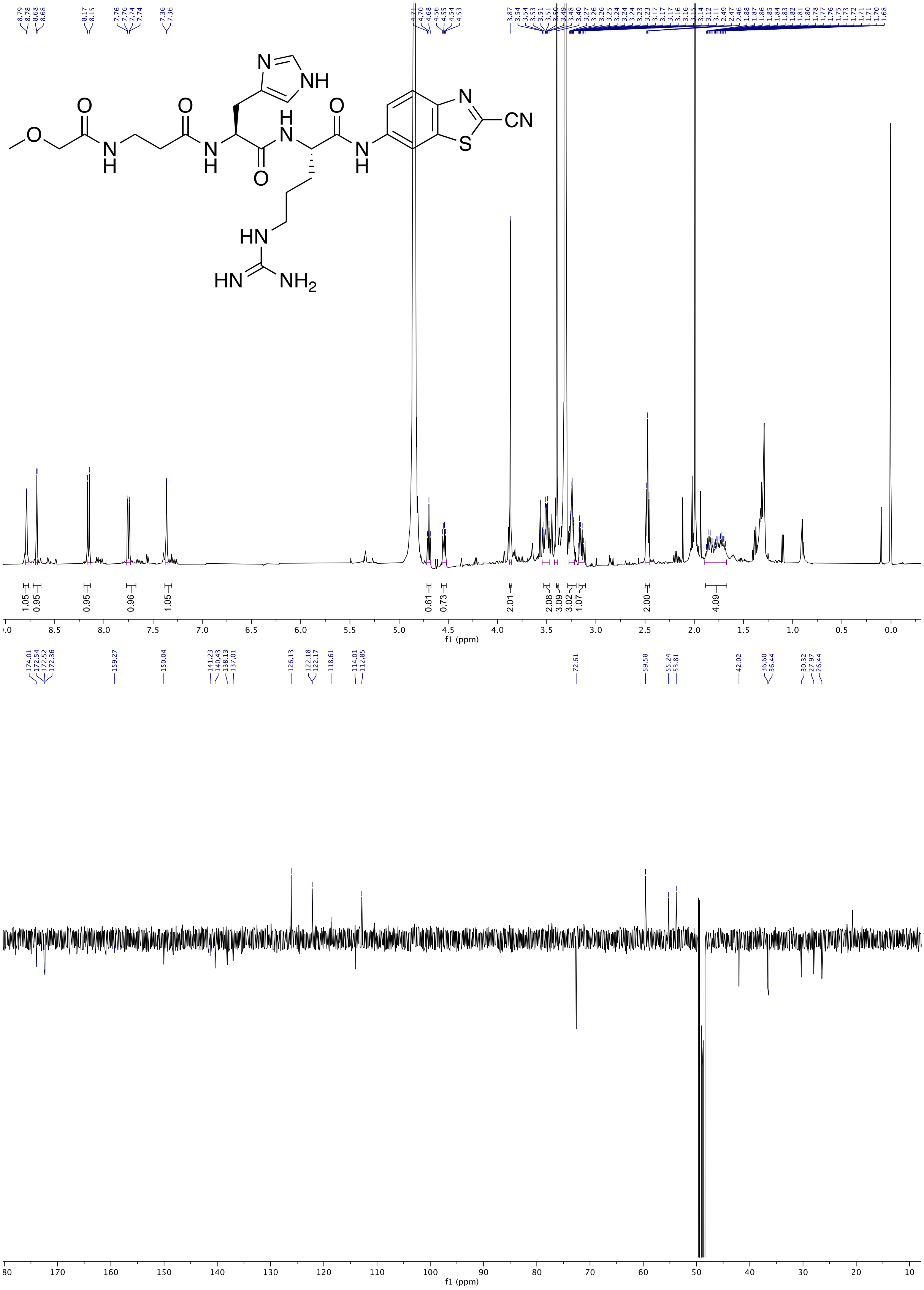

Figure S26: ^{1}H and ^{13}C NMR spectra of methoxyacetyl-βAHR-6ABTC.

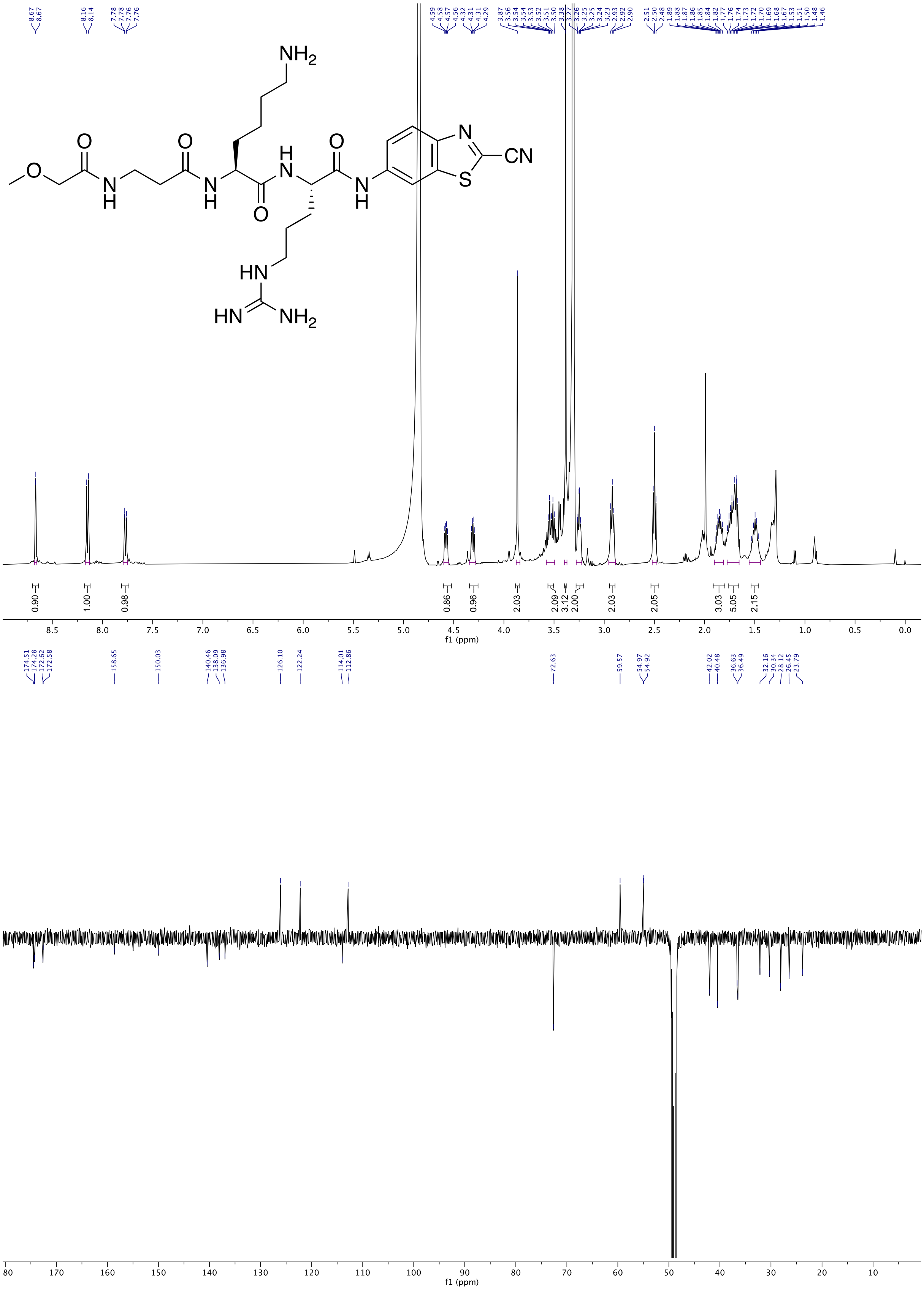

Figure S27: ^{1}H and ^{13}C NMR spectra of methoxyacetyl-βAKR-6ABTC.

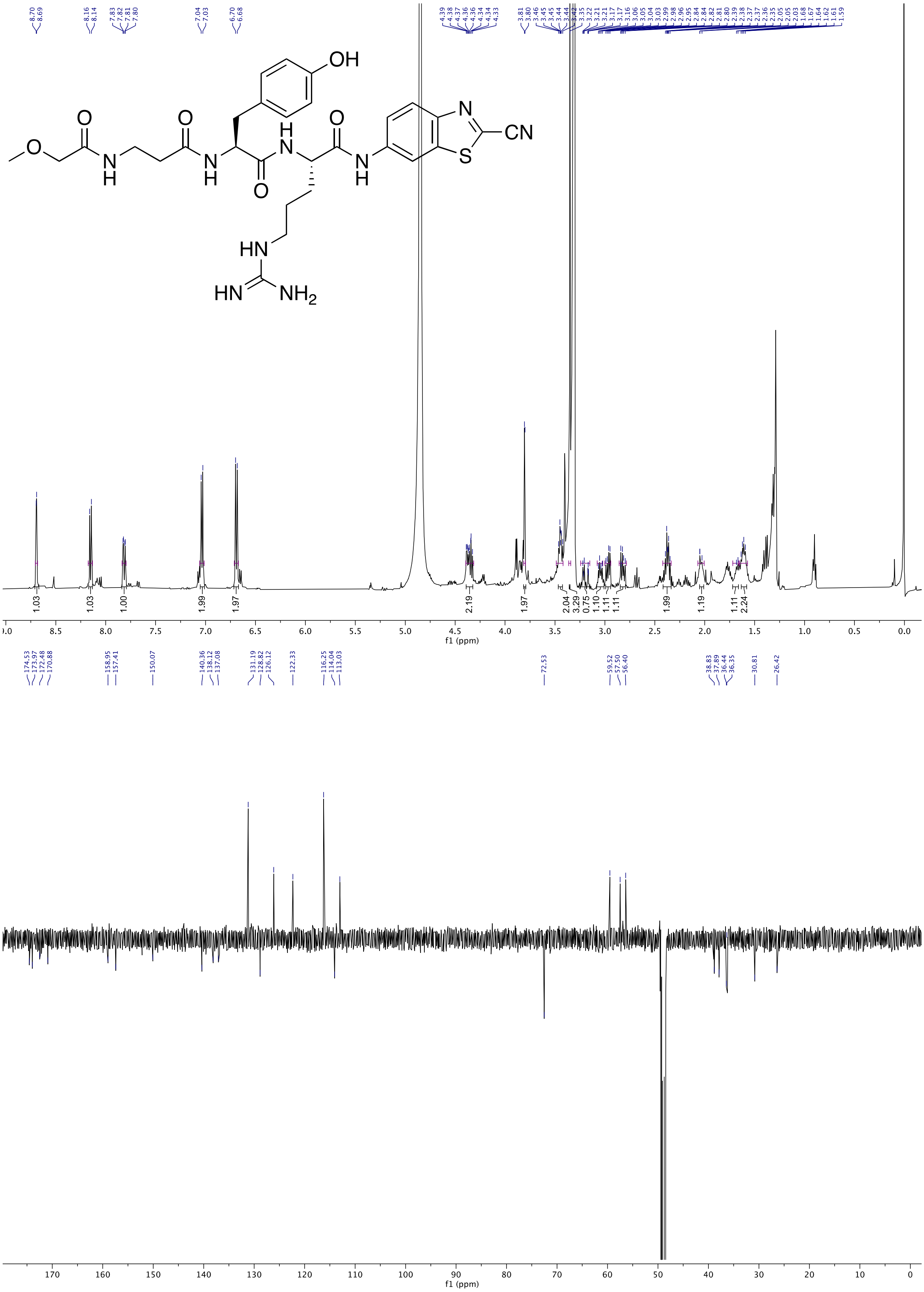

Figure S28: ^{1}H and ^{13}C NMR spectra of methoxyacetyl-βAYR-6ABTC.

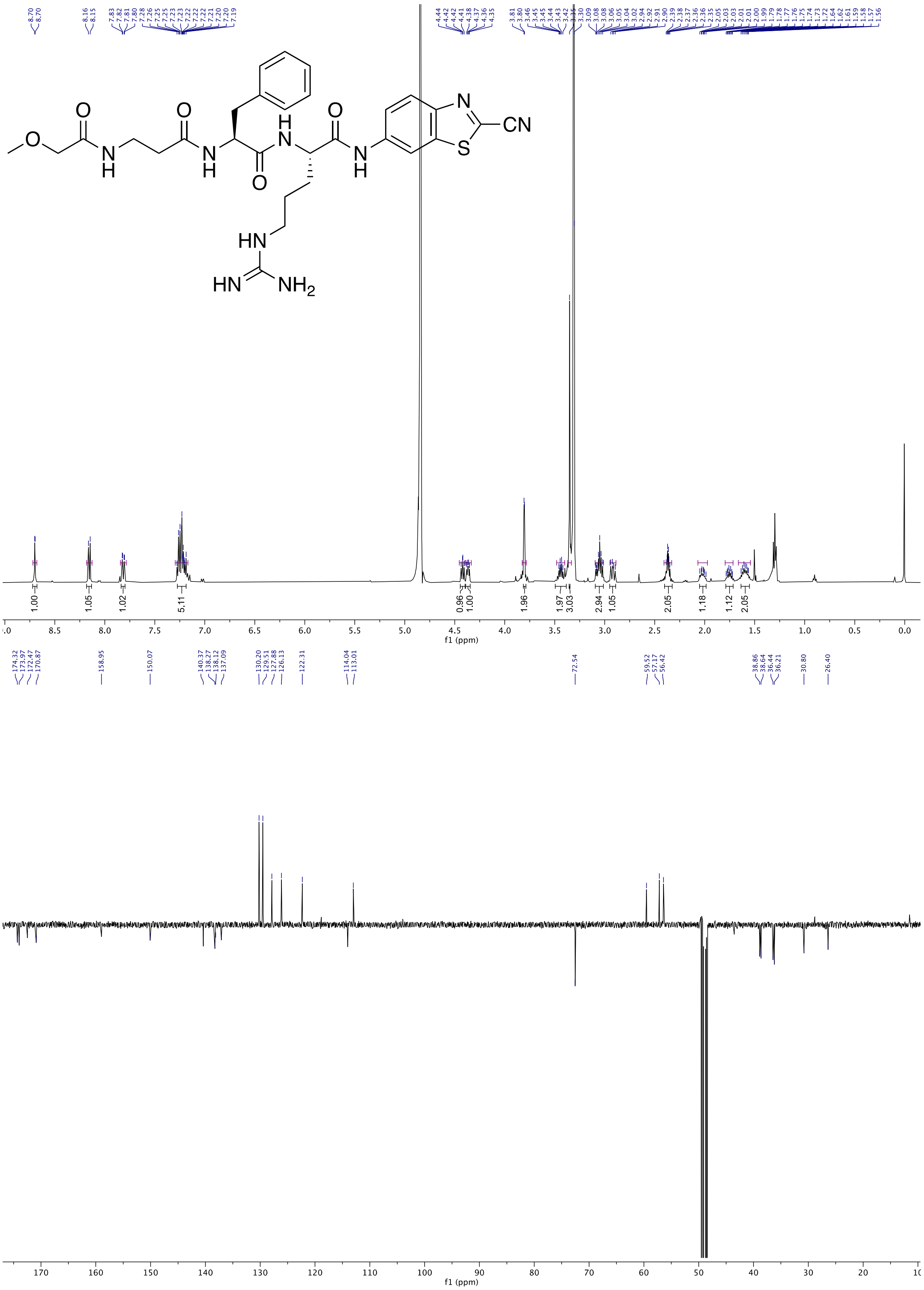

Figure S29: ^{1}H and ^{13}C NMR spectra of methoxyacetyl-βAFR-6ABTC.

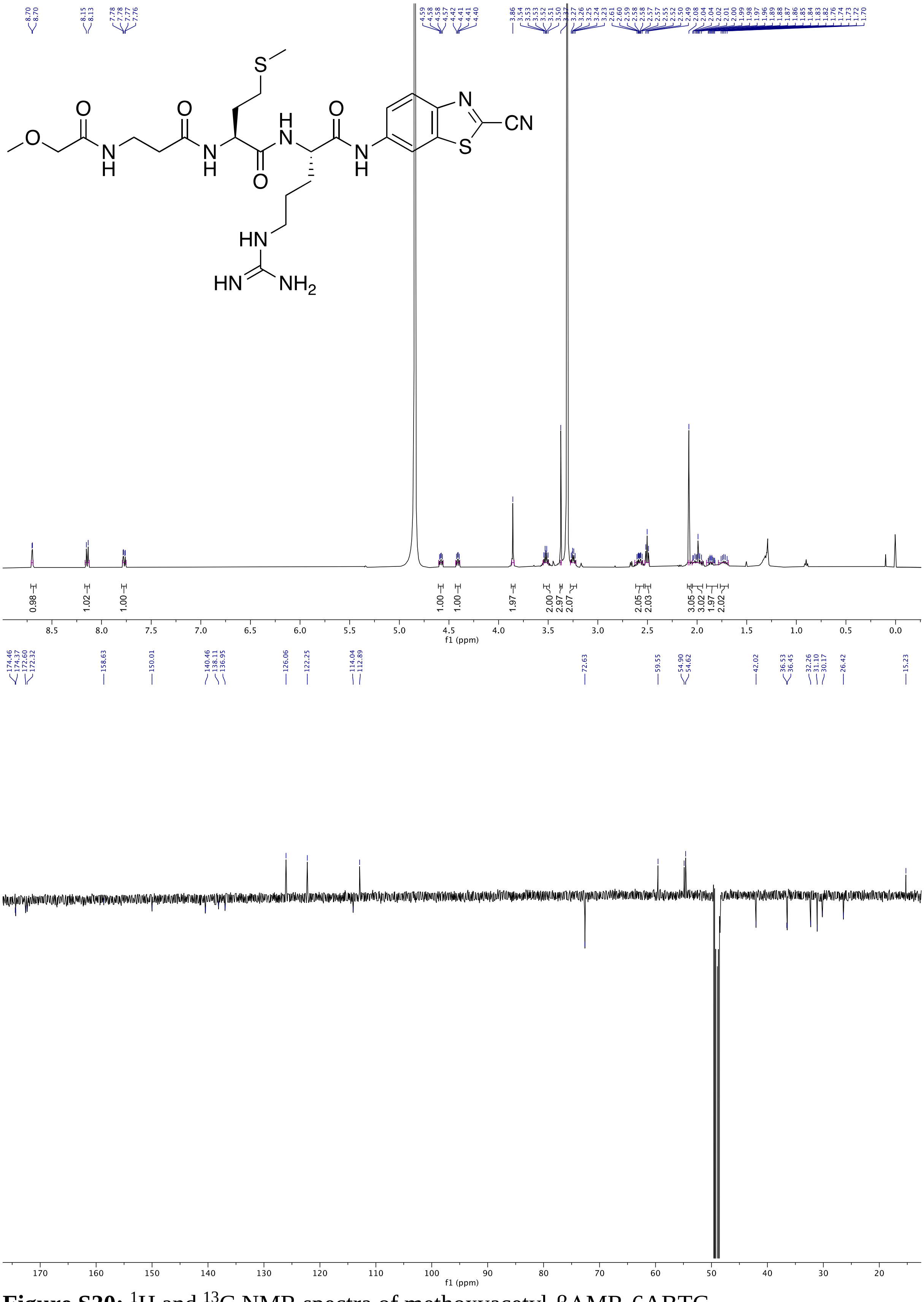

Figure S30: ^{1}H and ^{13}C NMR spectra of methoxyacetyl-βAMR-6ABTC.

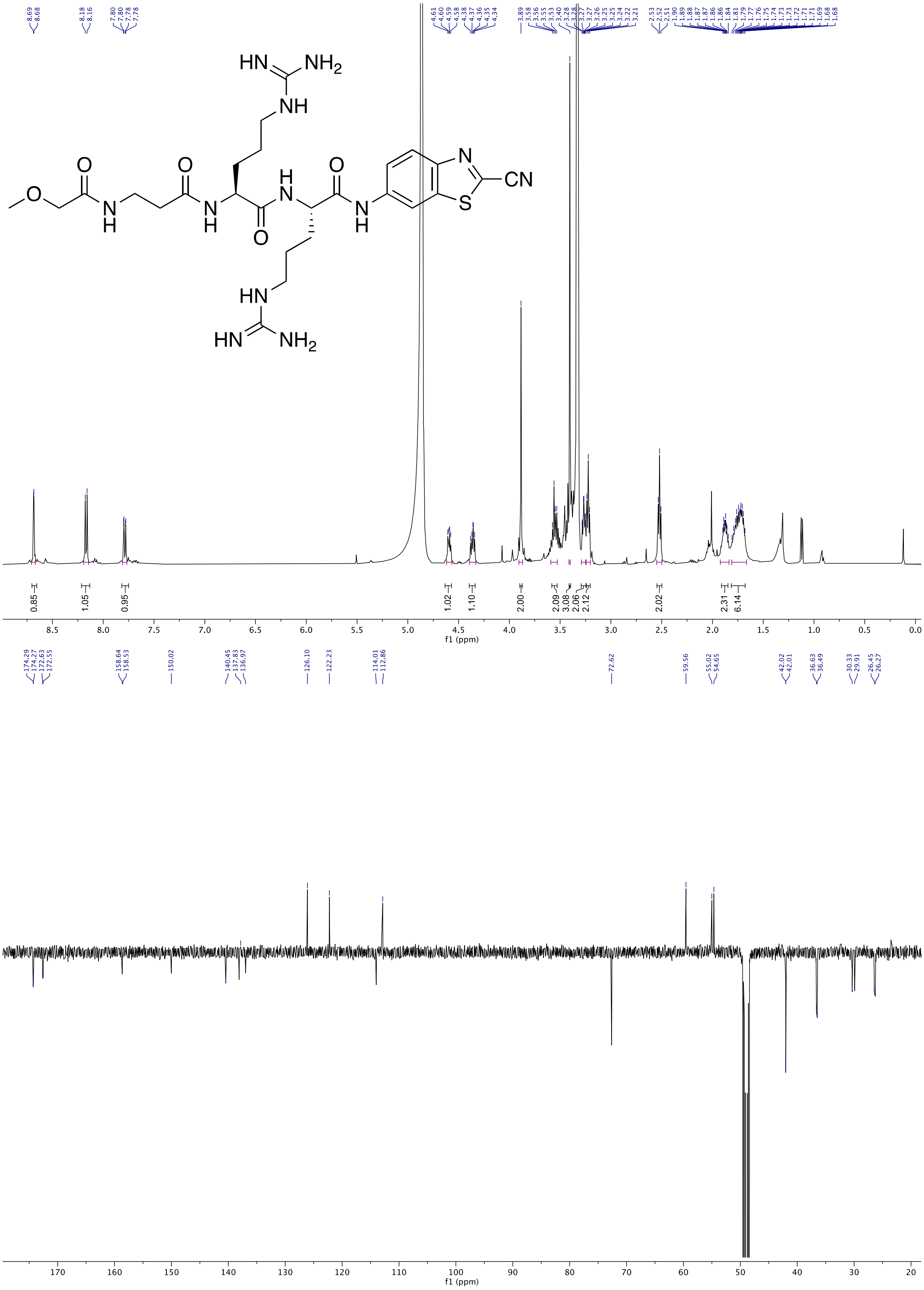

Figure S31: ^{1}H and ^{13}C NMR spectra of methoxyacetyl-βARR-6ABTC.

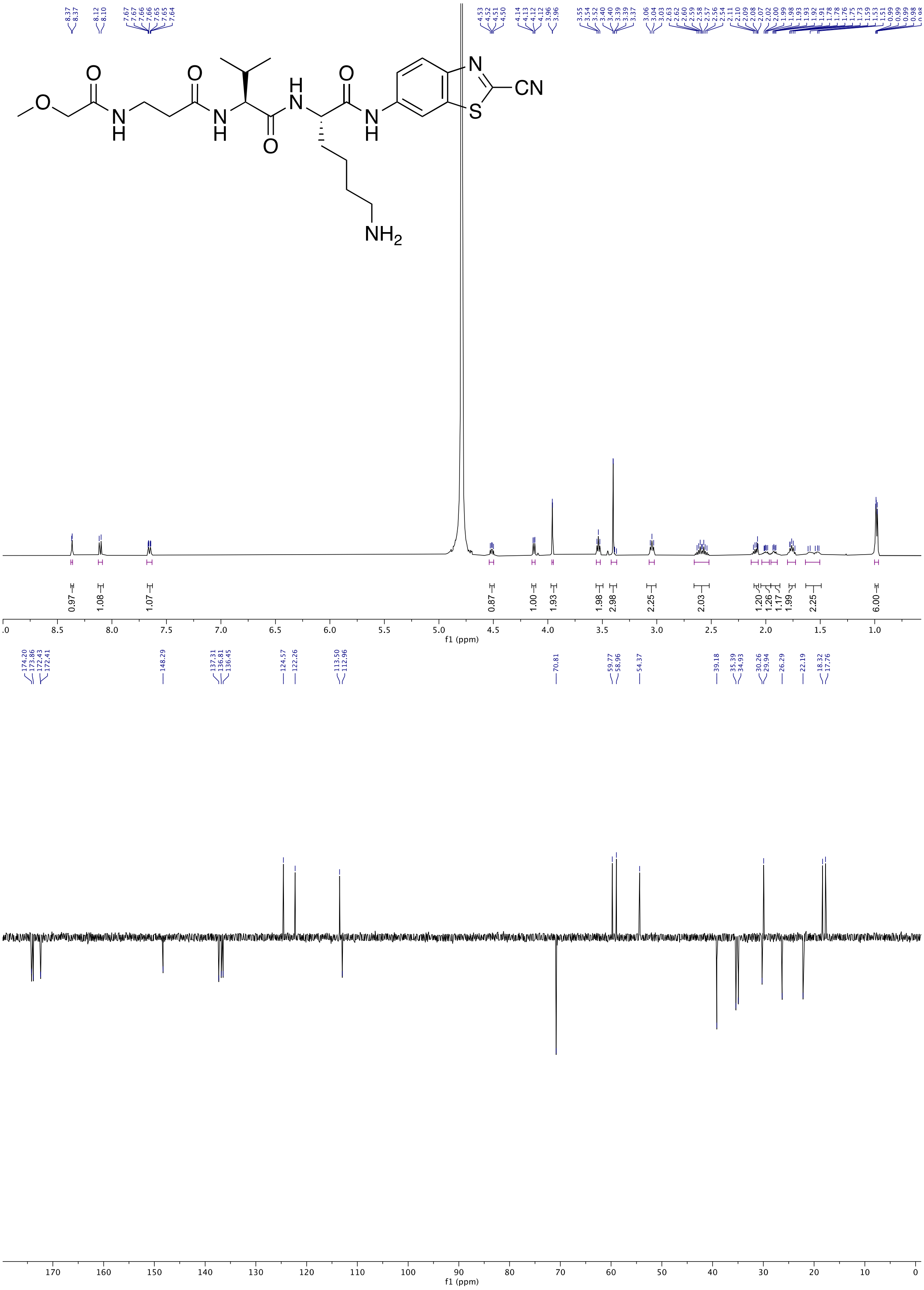

Figure S32: ^{1}H and ^{13}C NMR spectra of methoxyacetyl-βAVK-6ABTC.

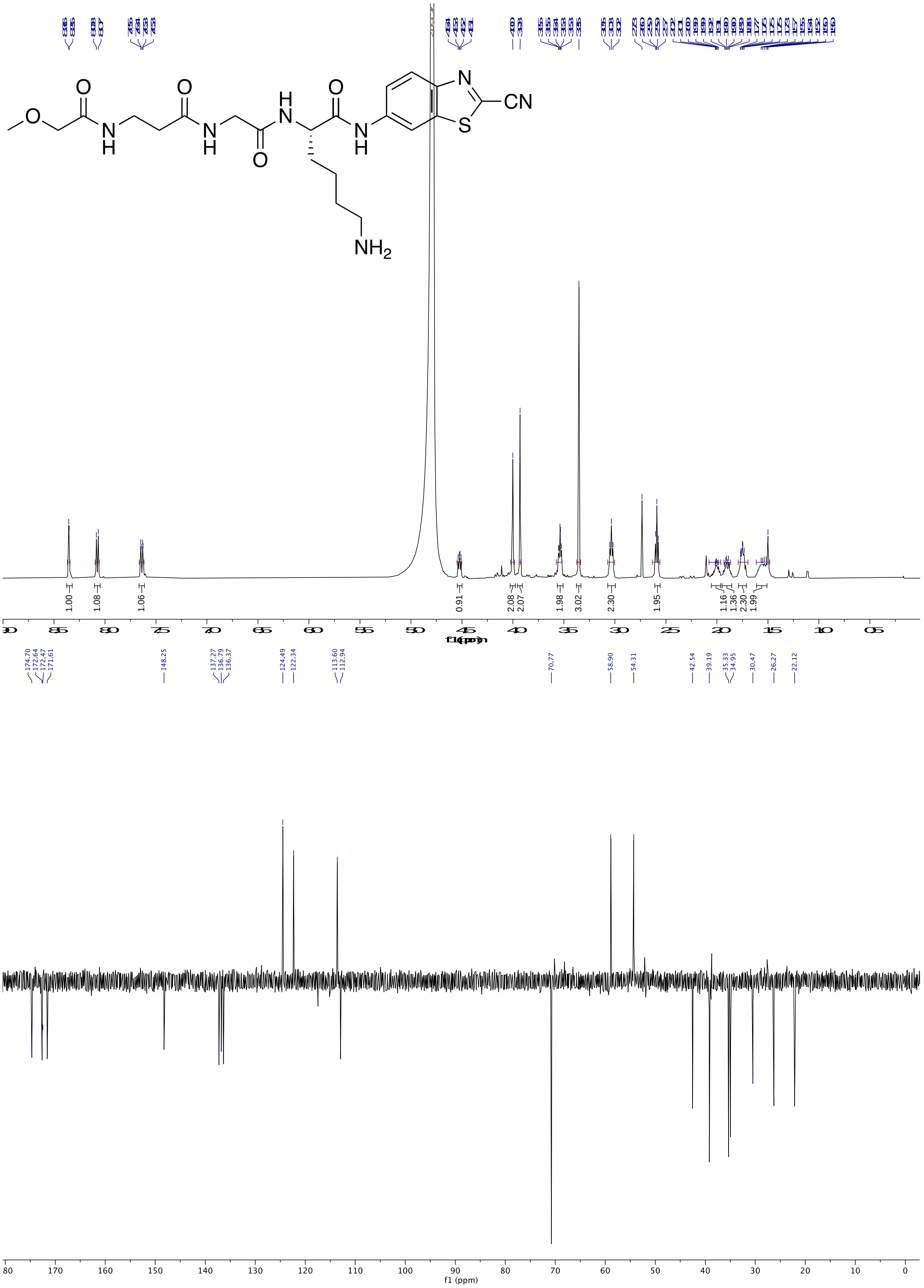

Figure S33: ^{1}H and ^{13}C NMR spectra of methoxyacetyl-βAGK-6ABTC.

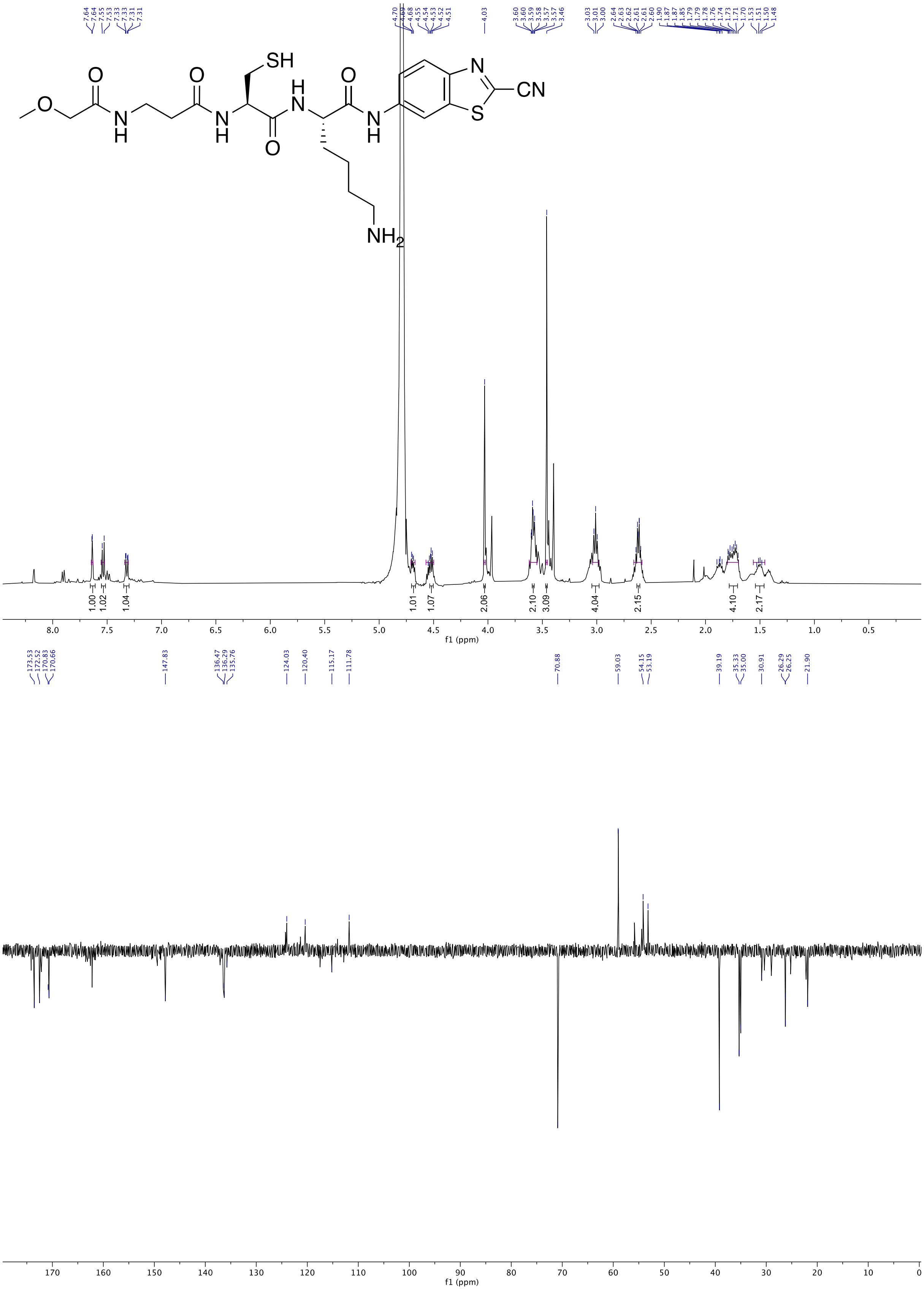

Figure S34: ^{1}H and ^{13}C NMR spectra of methoxyacetyl-βACK-6ABTC.

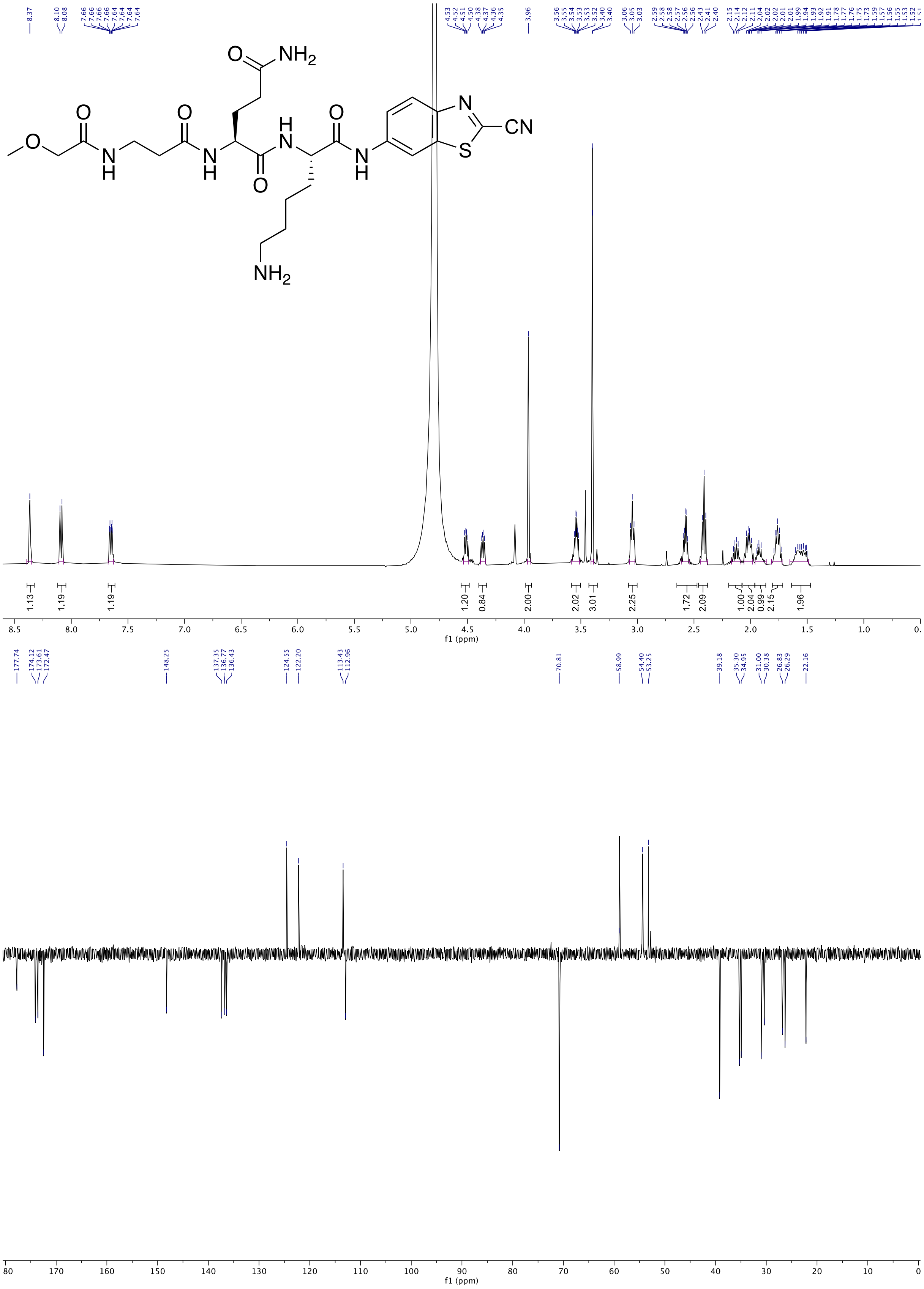

Figure S35: ^{1}H and ^{13}C NMR spectra of methoxyacetyl-βAEK-6ABTC.

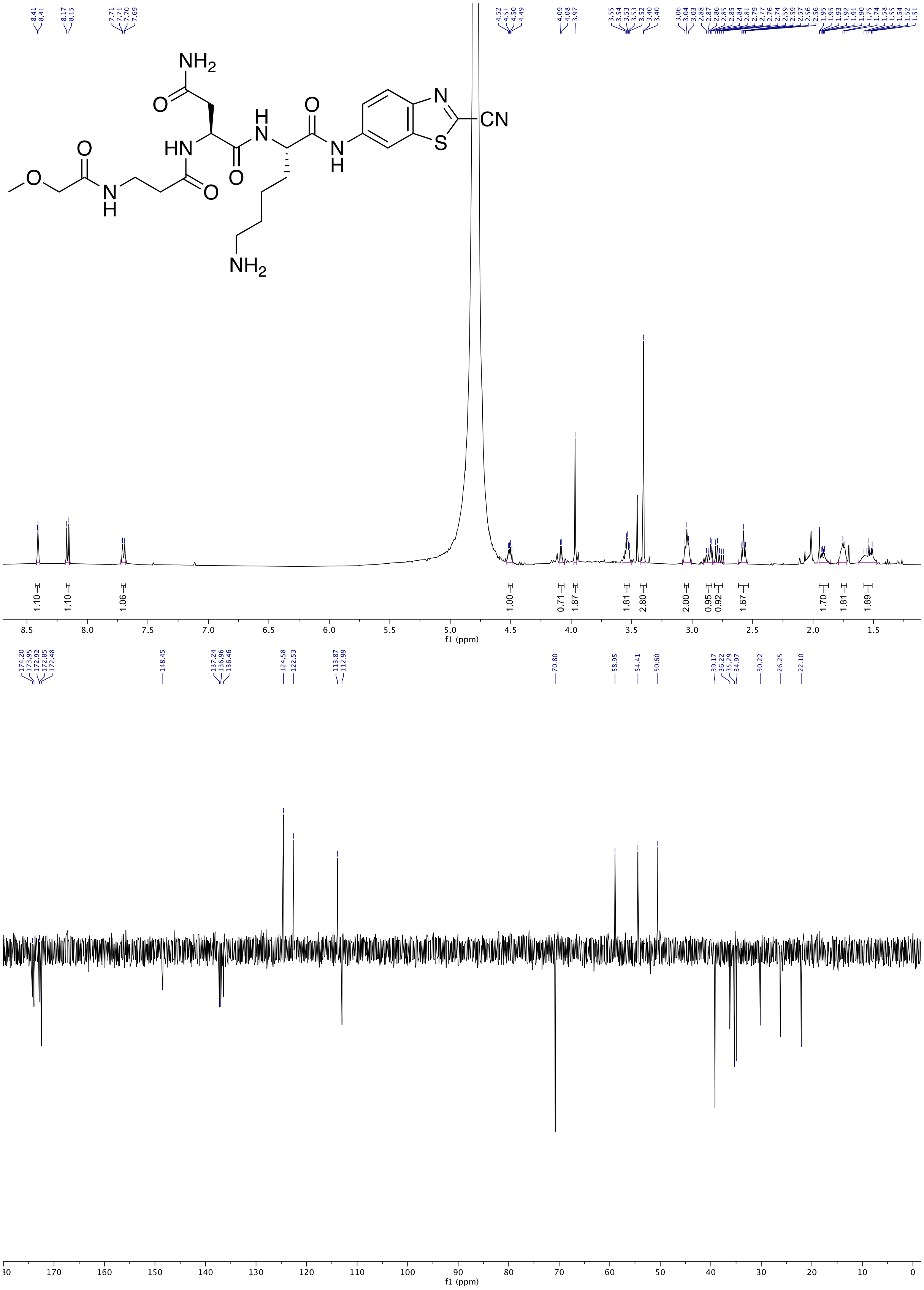

Figure S36: ^{1}H and ^{13}C NMR spectra of methoxyacetyl-βADK-6ABTC.

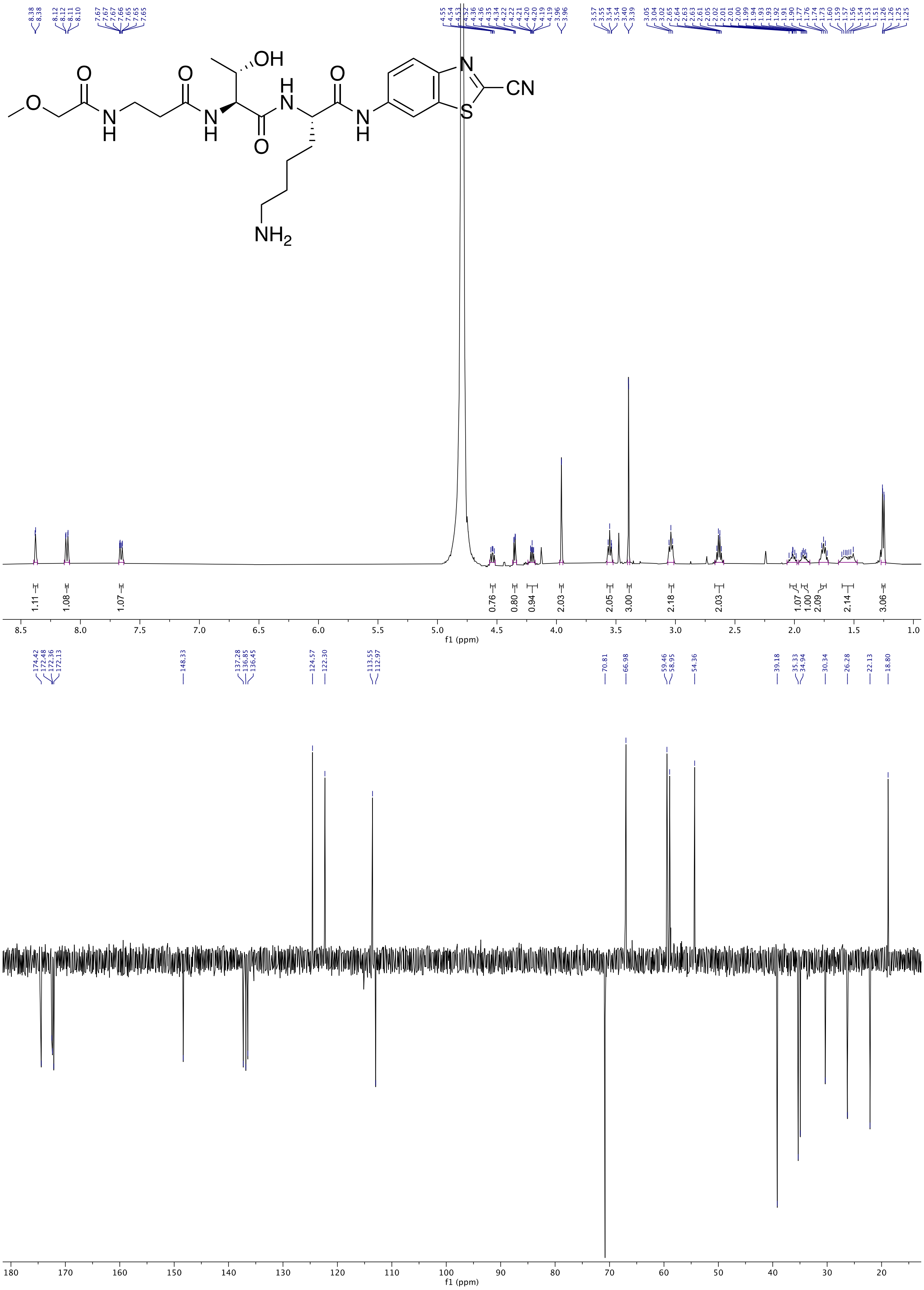

Figure S37: ^{1}H and ^{13}C NMR spectra of methoxyacetyl-βATK-6ABTC.

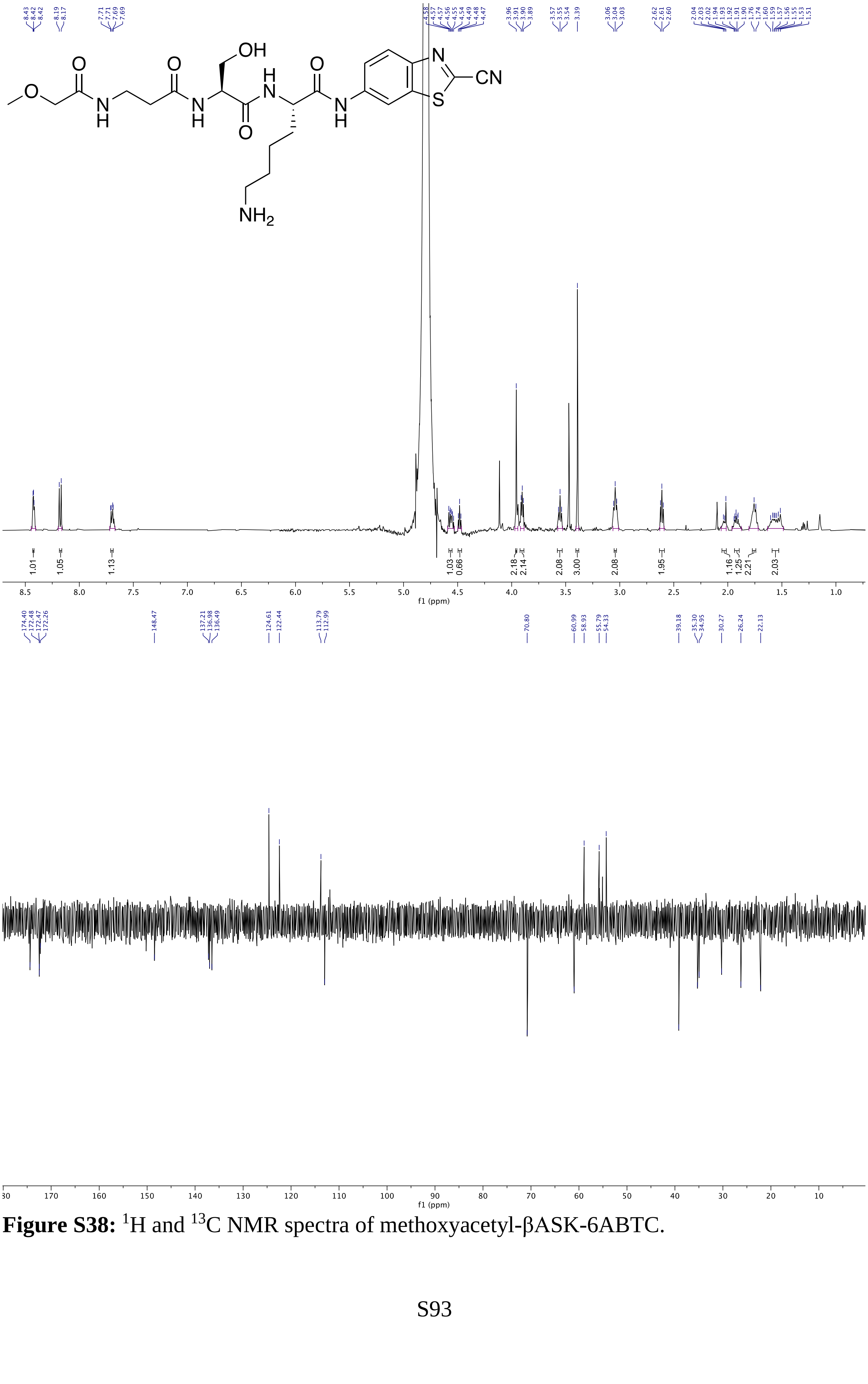

Figure S38: ^{1}H and ^{13}C NMR spectra of methoxyacetyl-βASK-6ABTC.

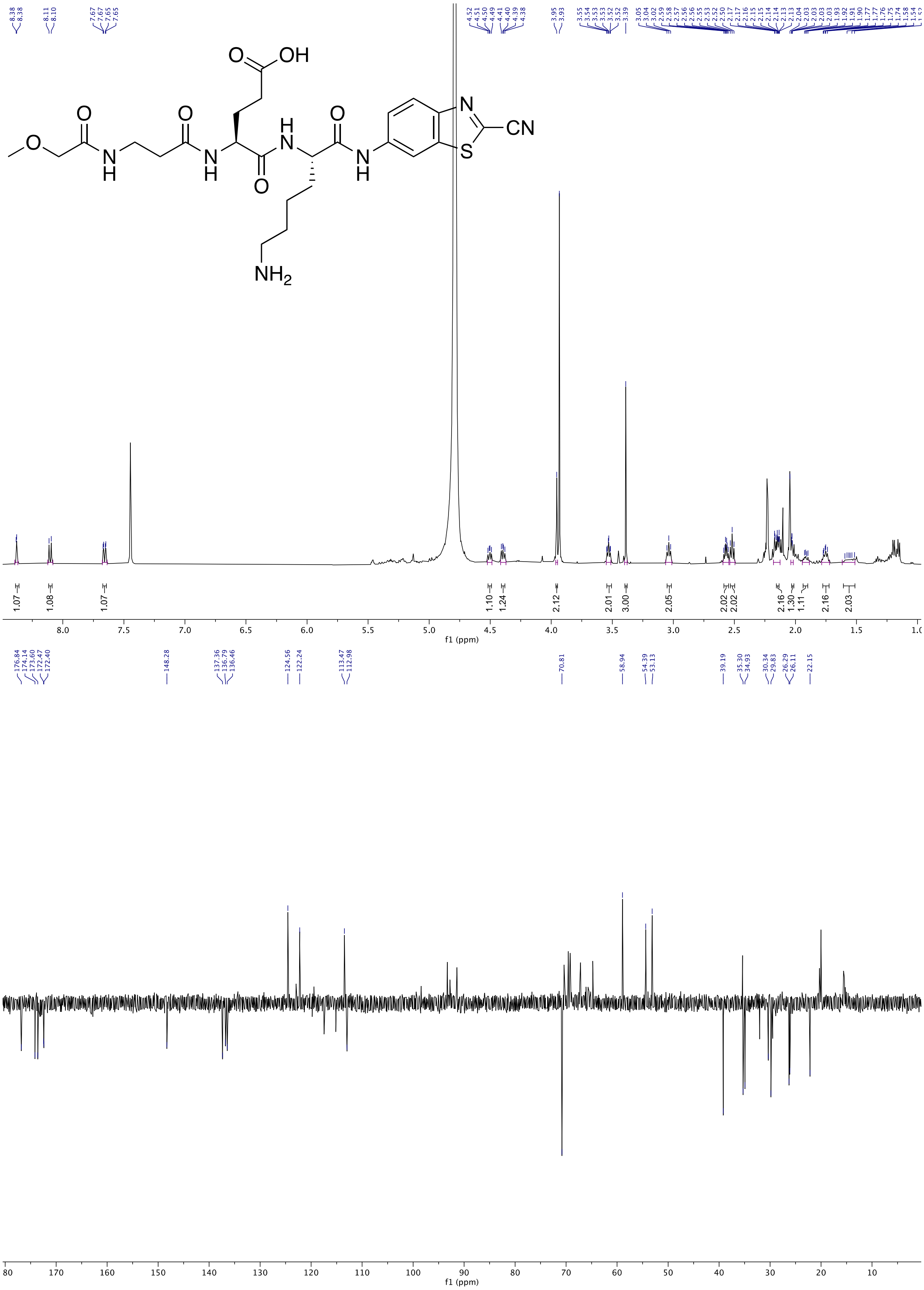

Figure S39: ^{1}H and ^{13}C NMR spectra of methoxyacetyl-βAQK-6ABTC.

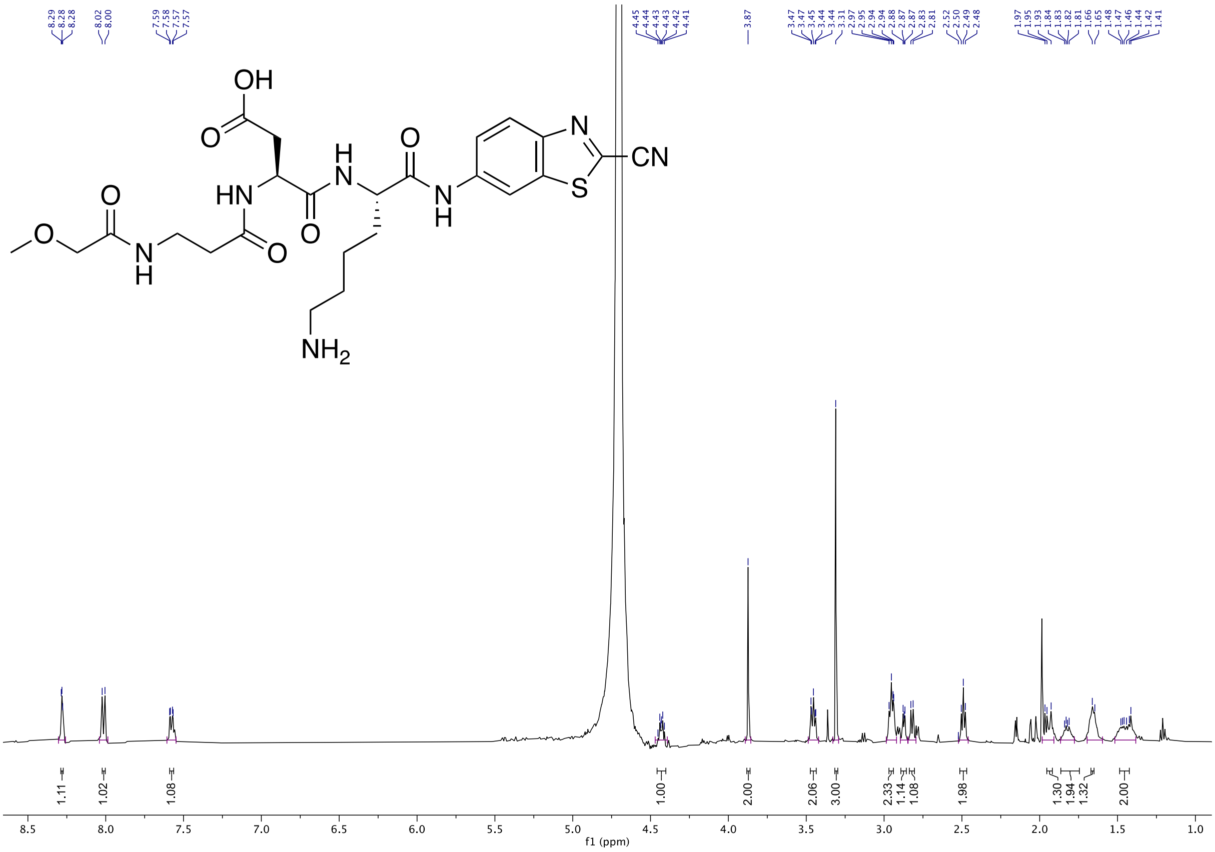

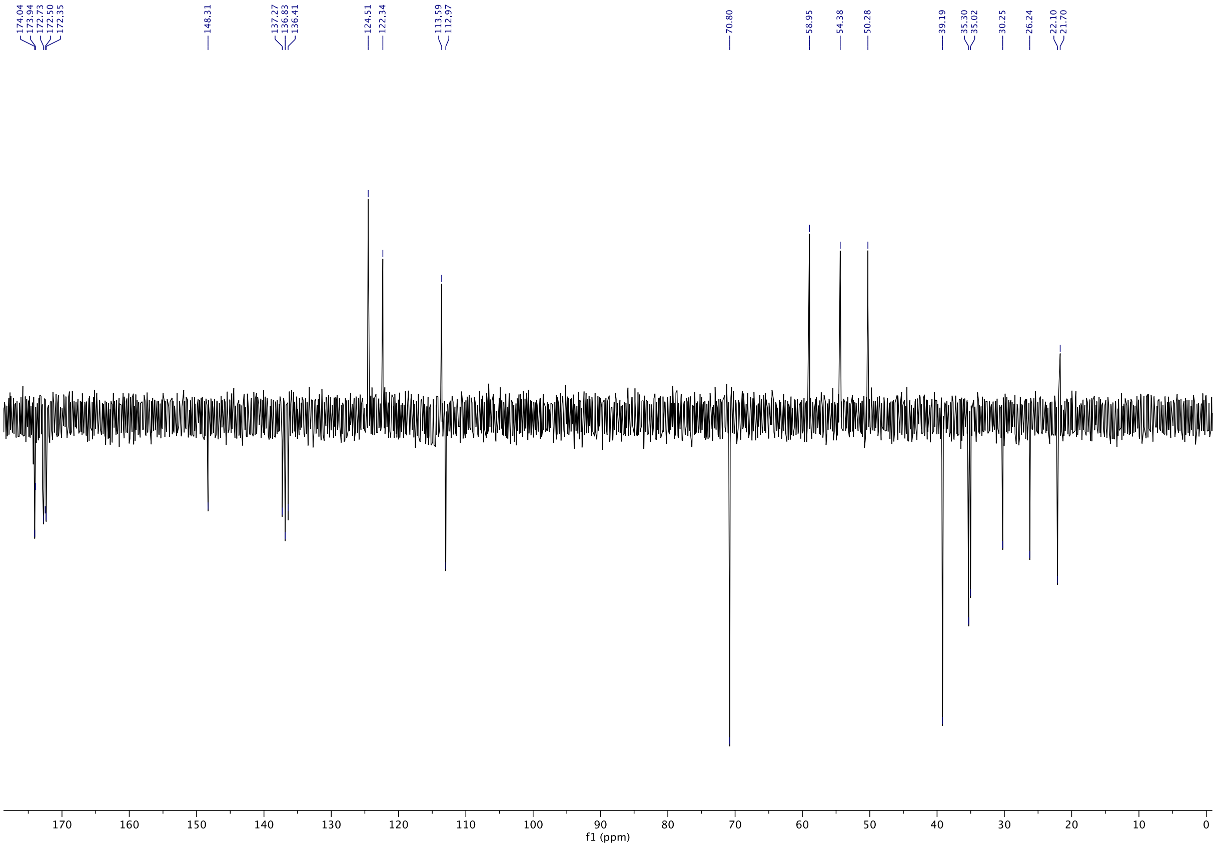

Figure S40: ^{1}H and ^{13}C NMR spectra of methoxyacetyl-βANK-6ABTC.

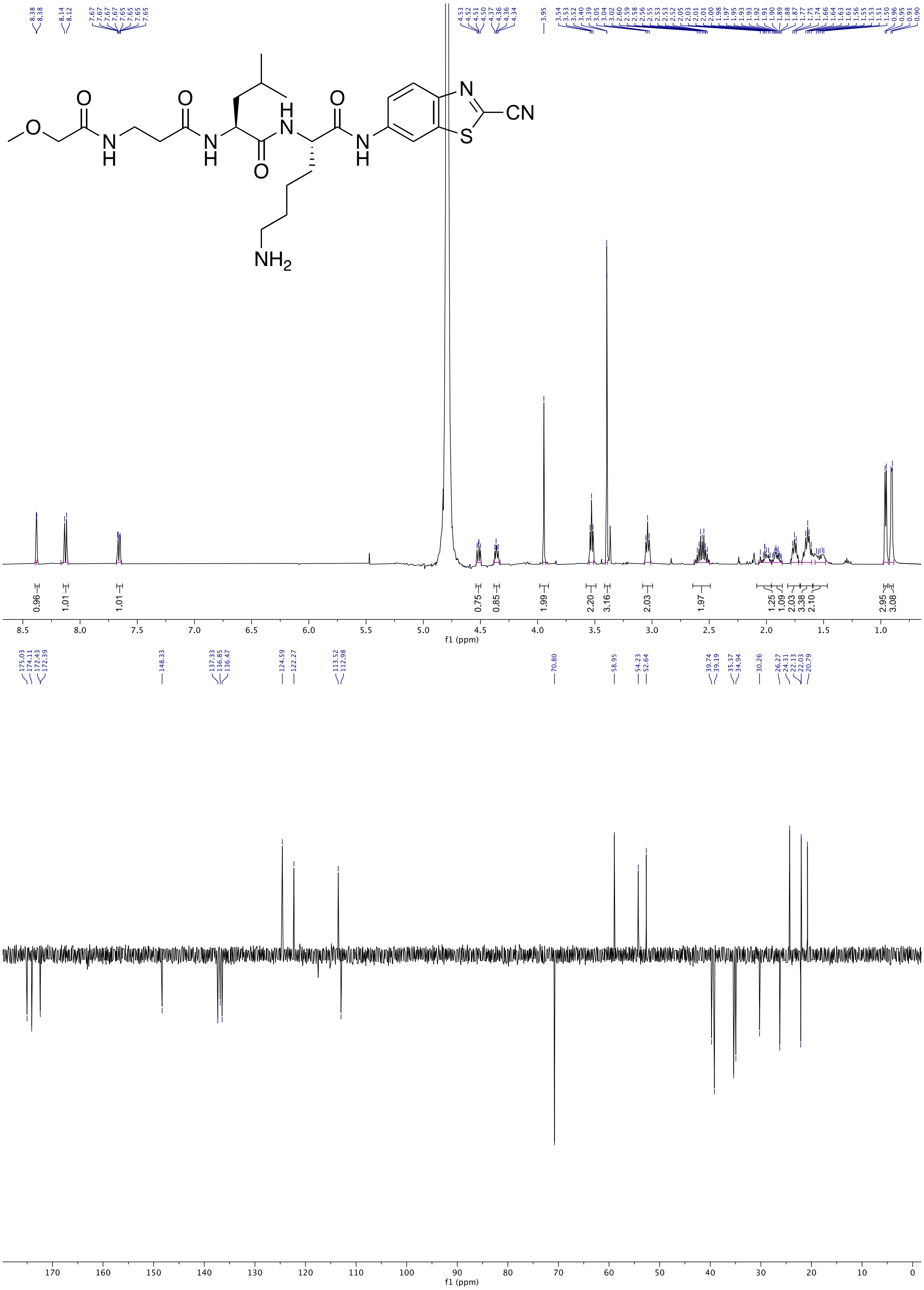

Figure S41: ^{1}H and ^{13}C NMR spectra of methoxyacetyl-βALK-6ABTC.

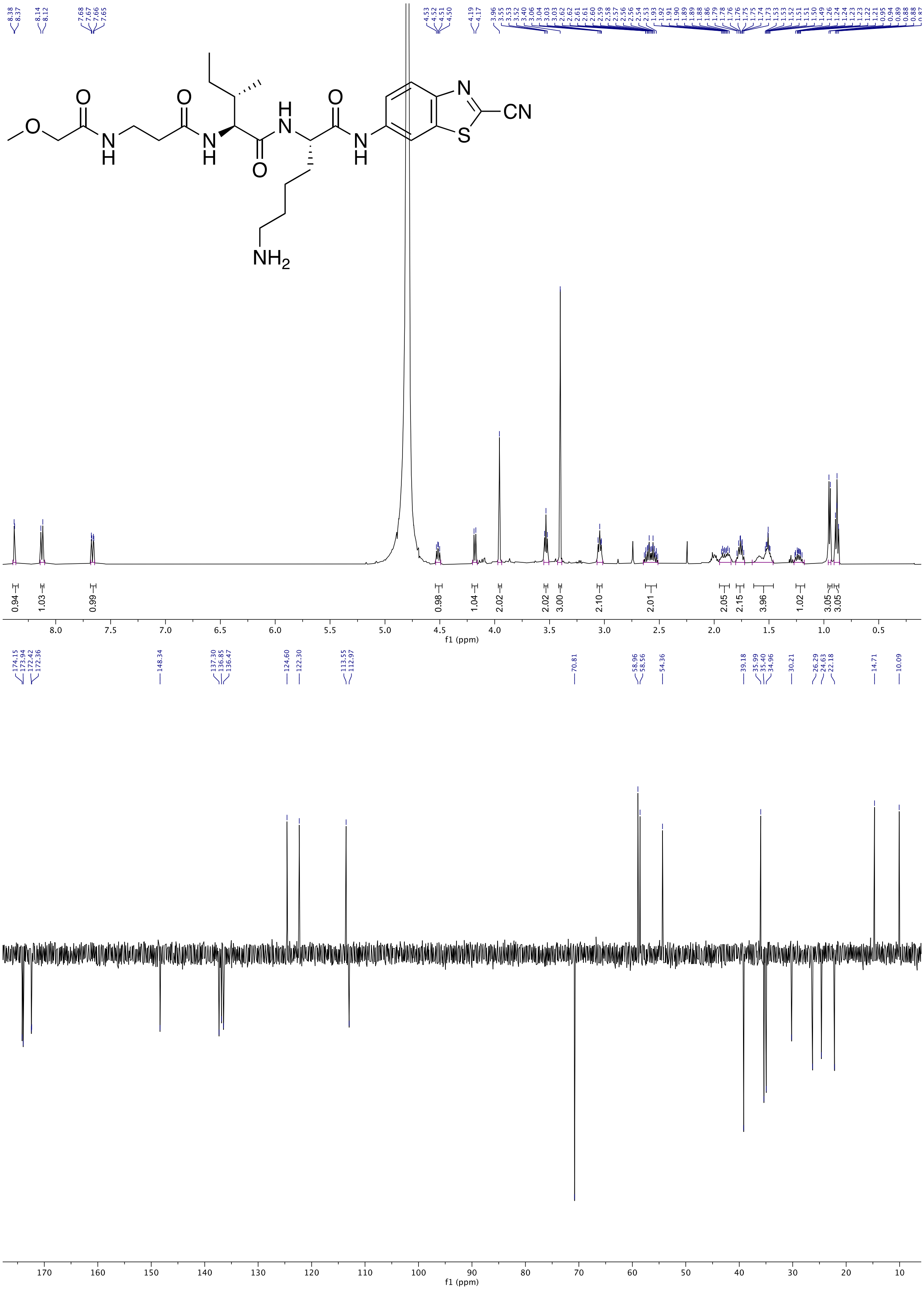

Figure S42: ^{1}H and ^{13}C NMR spectra of methoxyacetyl-βAIK-6ABTC.

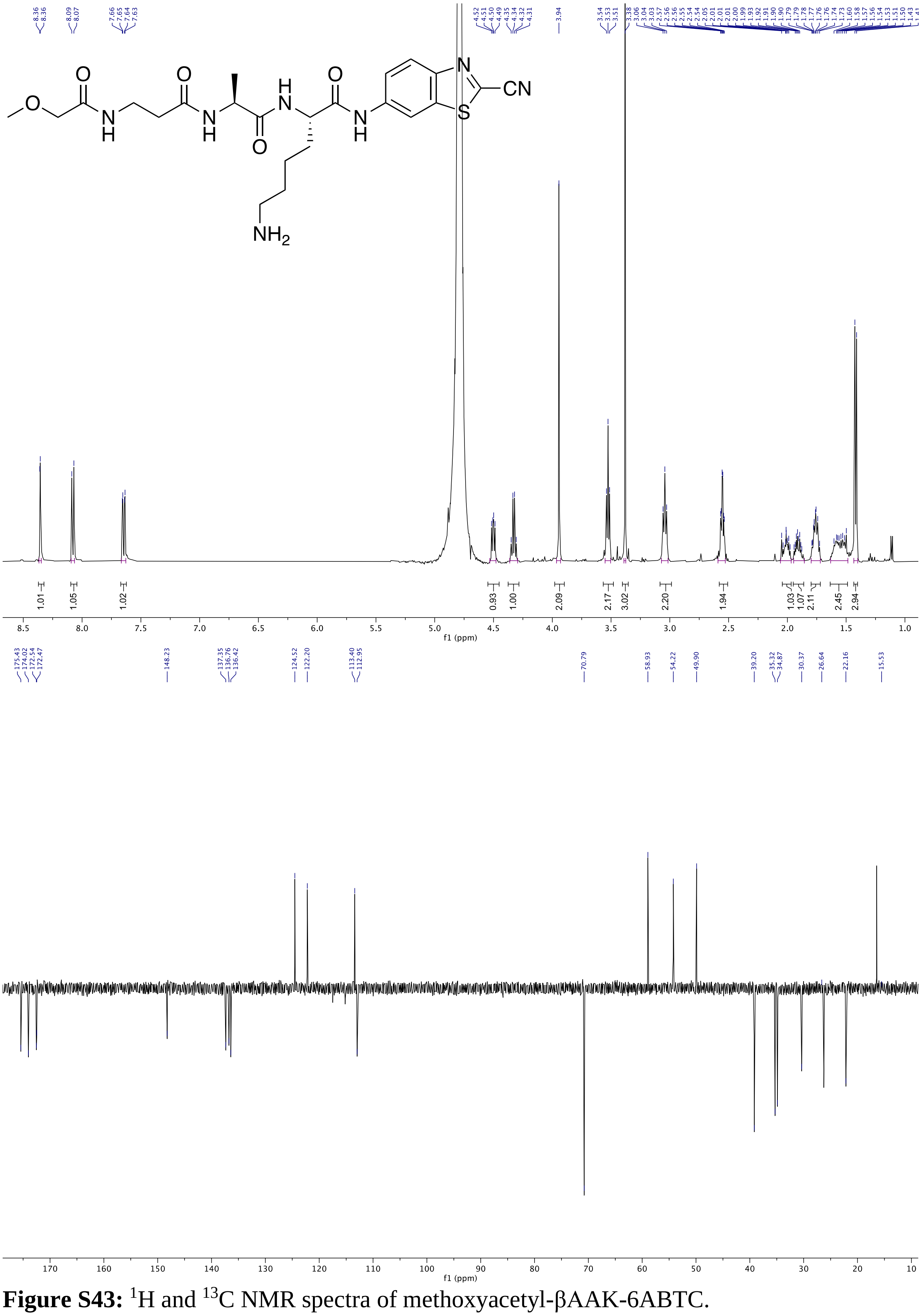

Figure S43: ^{1}H and ^{13}C NMR spectra of methoxyacetyl-βAAK-6ABTC.

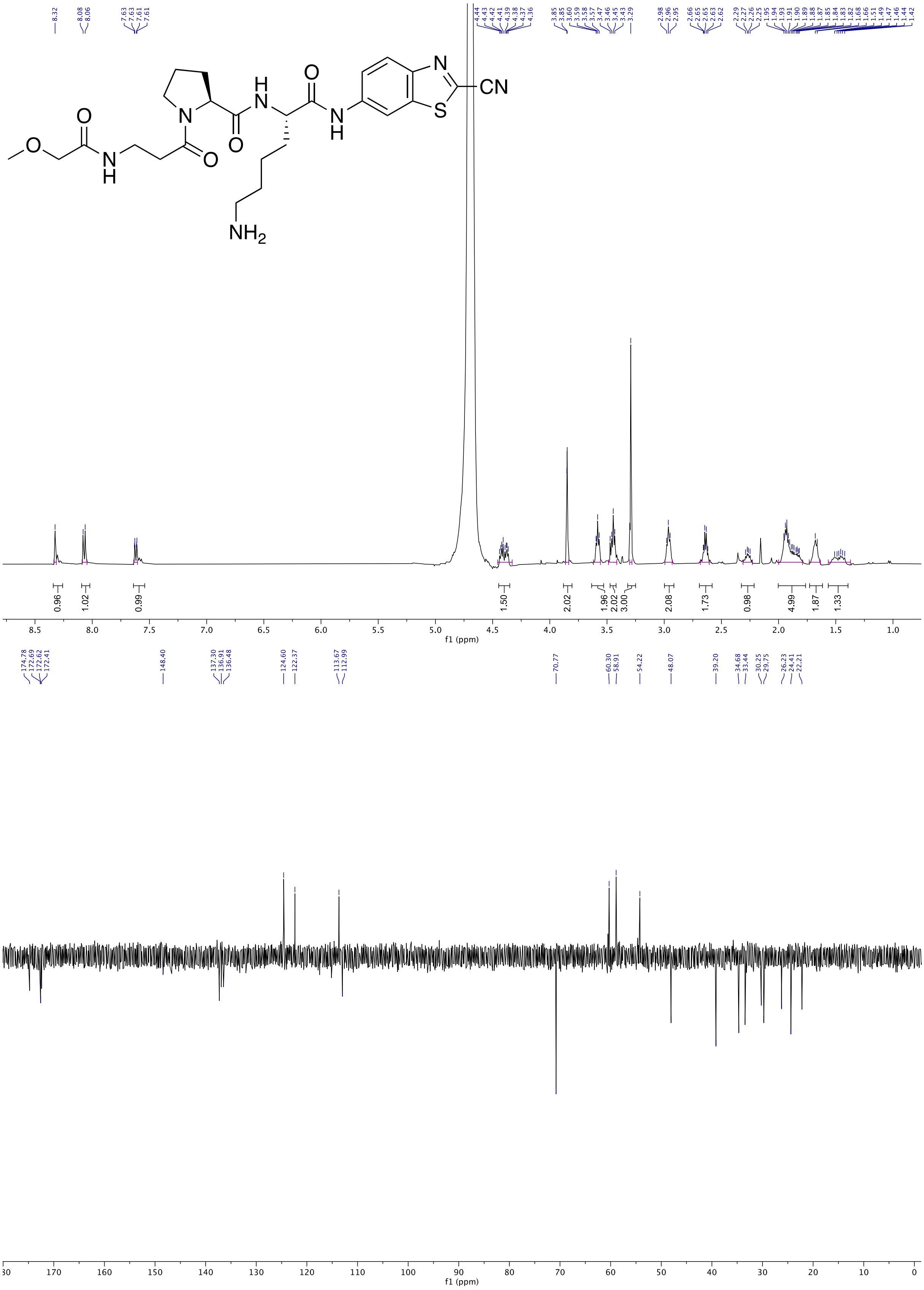

Figure S44: ^{1}H and ^{13}C NMR spectra of methoxyacetyl-βAPK-6ABTC.

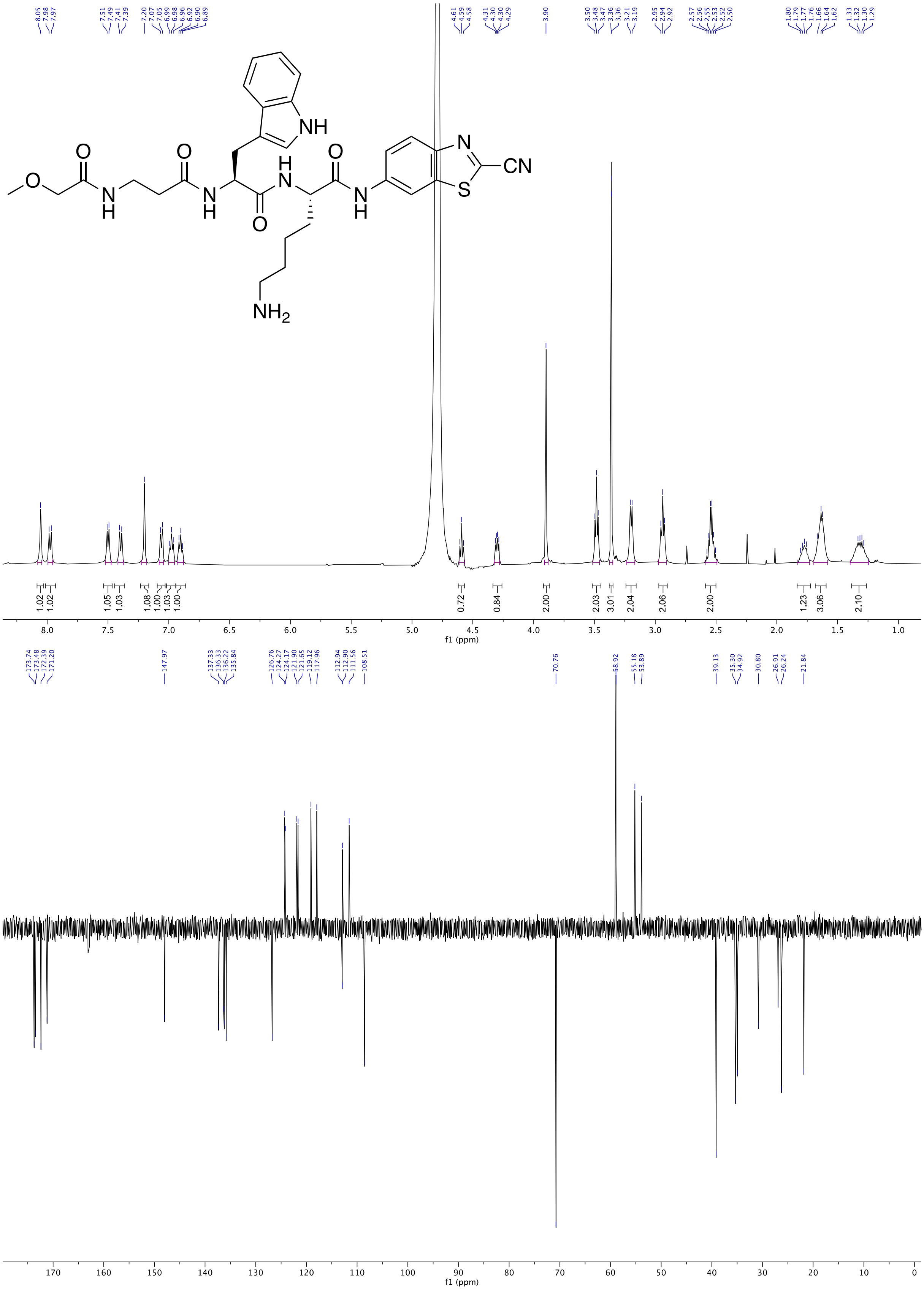

Figure S45: ^{1}H and ^{13}C NMR spectra of methoxyacetyl-βAWK-6ABTC.

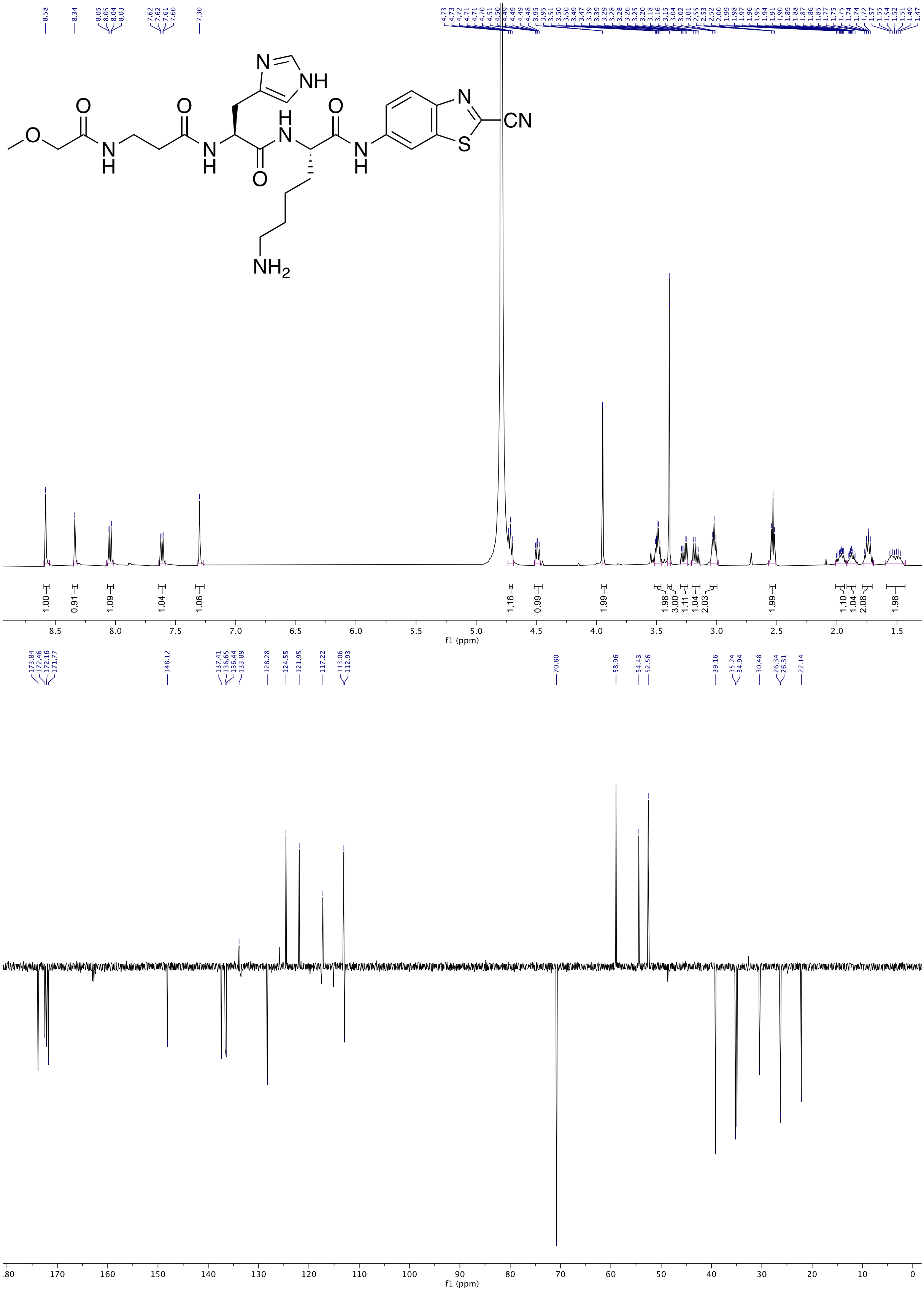

Figure S46: ^{1}H and ^{13}C NMR spectra of methoxyacetyl-βAHK-6ABTC.

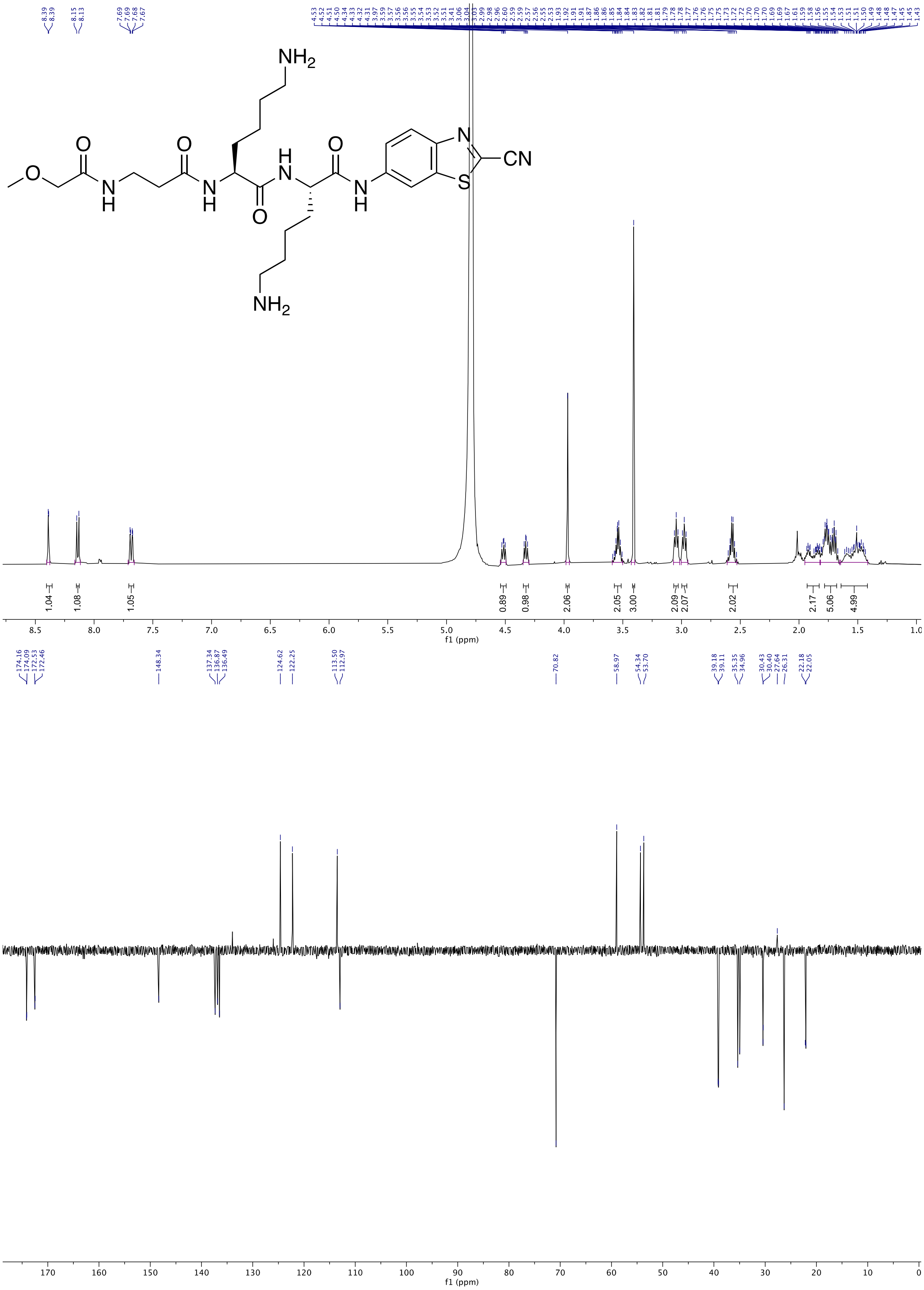

Figure S47: ^{1}H and ^{13}C NMR spectra of methoxyacetyl-βAKK-6ABTC.

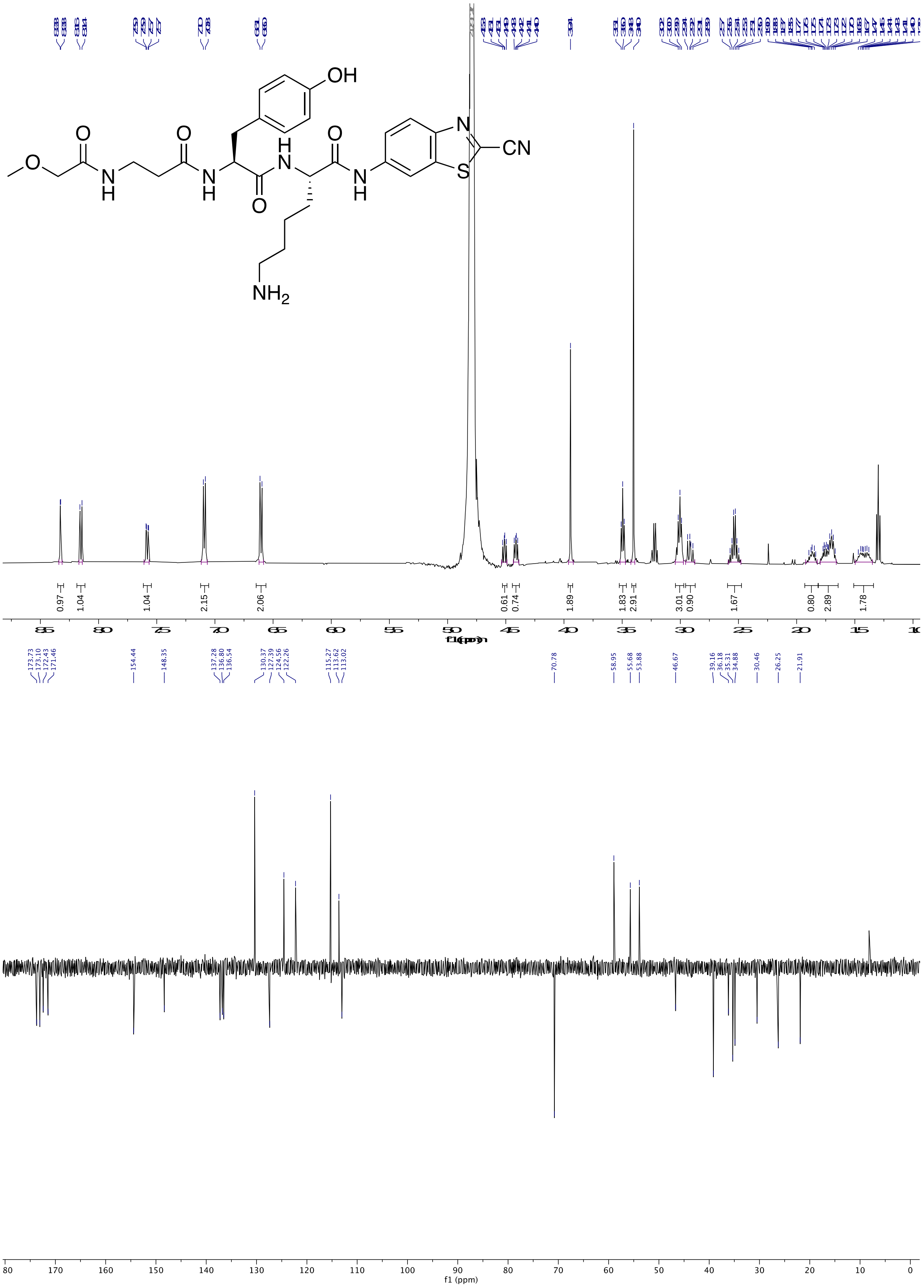

Figure S48: ^{1}H and ^{13}C NMR spectra of methoxyacetyl-βAYK-6ABTC.

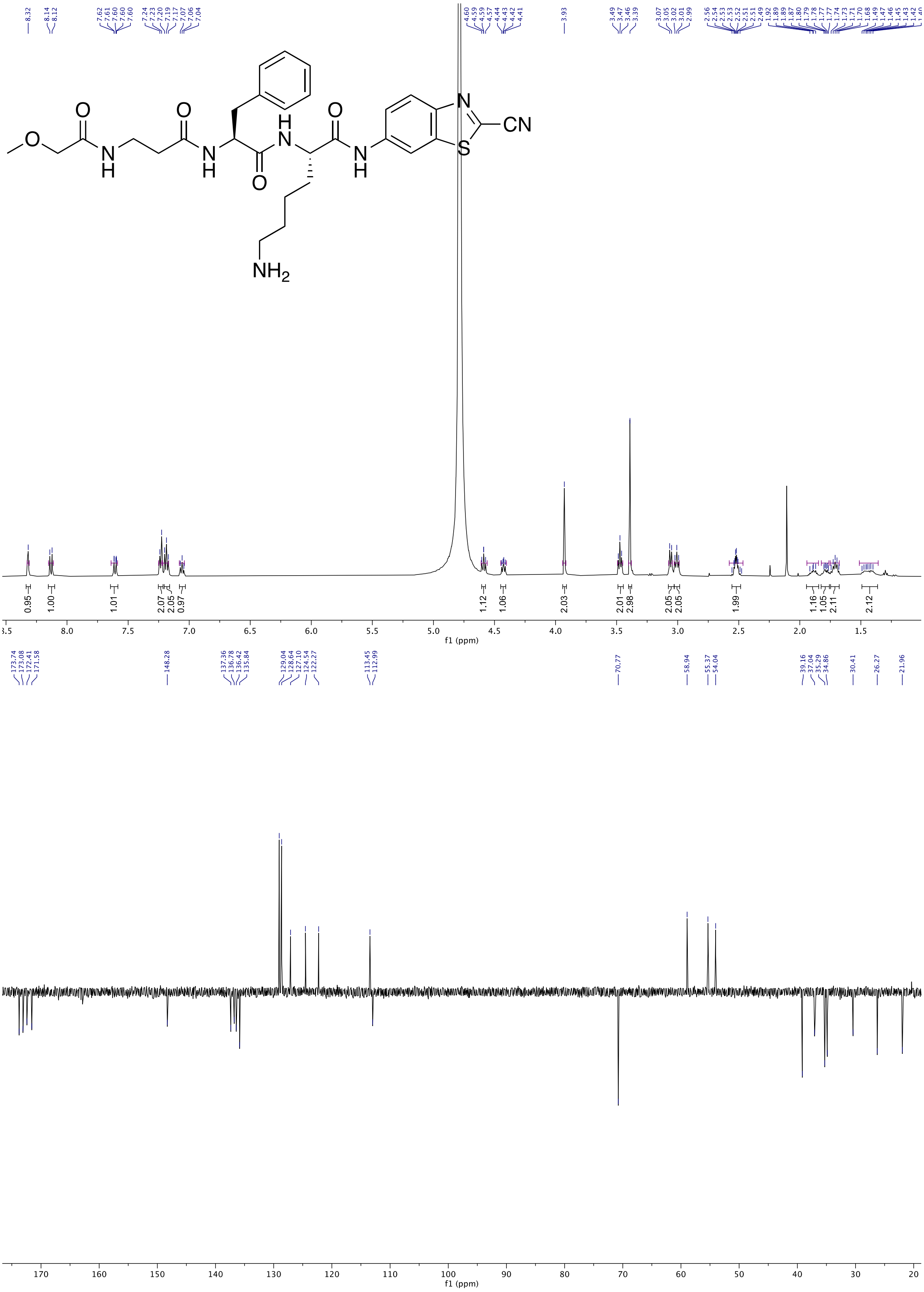

Figure S49: ^{1}H and ^{13}C NMR spectra of methoxyacetyl-βAFK-6ABTC.

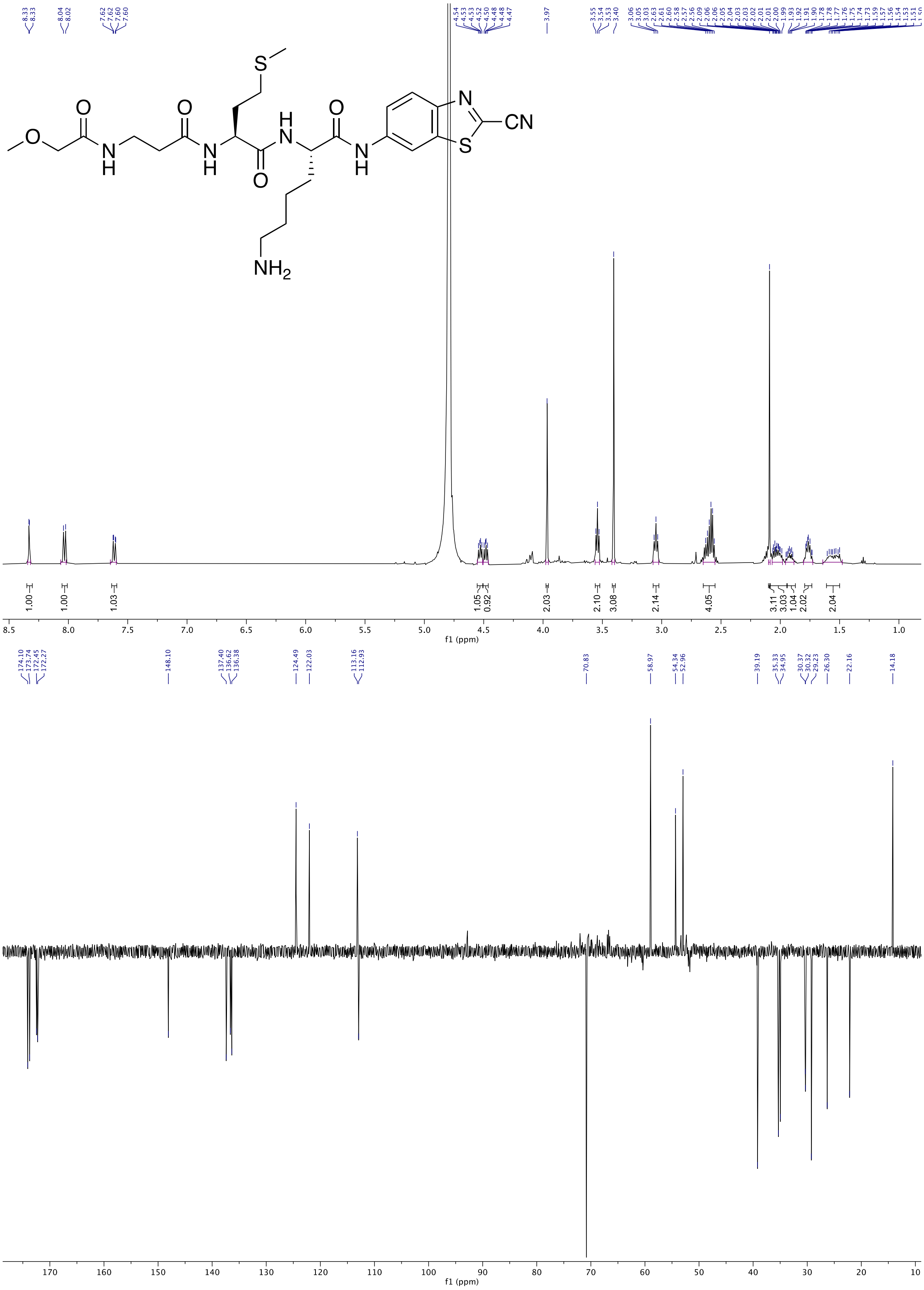

Figure S50: ^{1}H and ^{13}C NMR spectra of methoxyacetyl-βAMK-6ABTC.

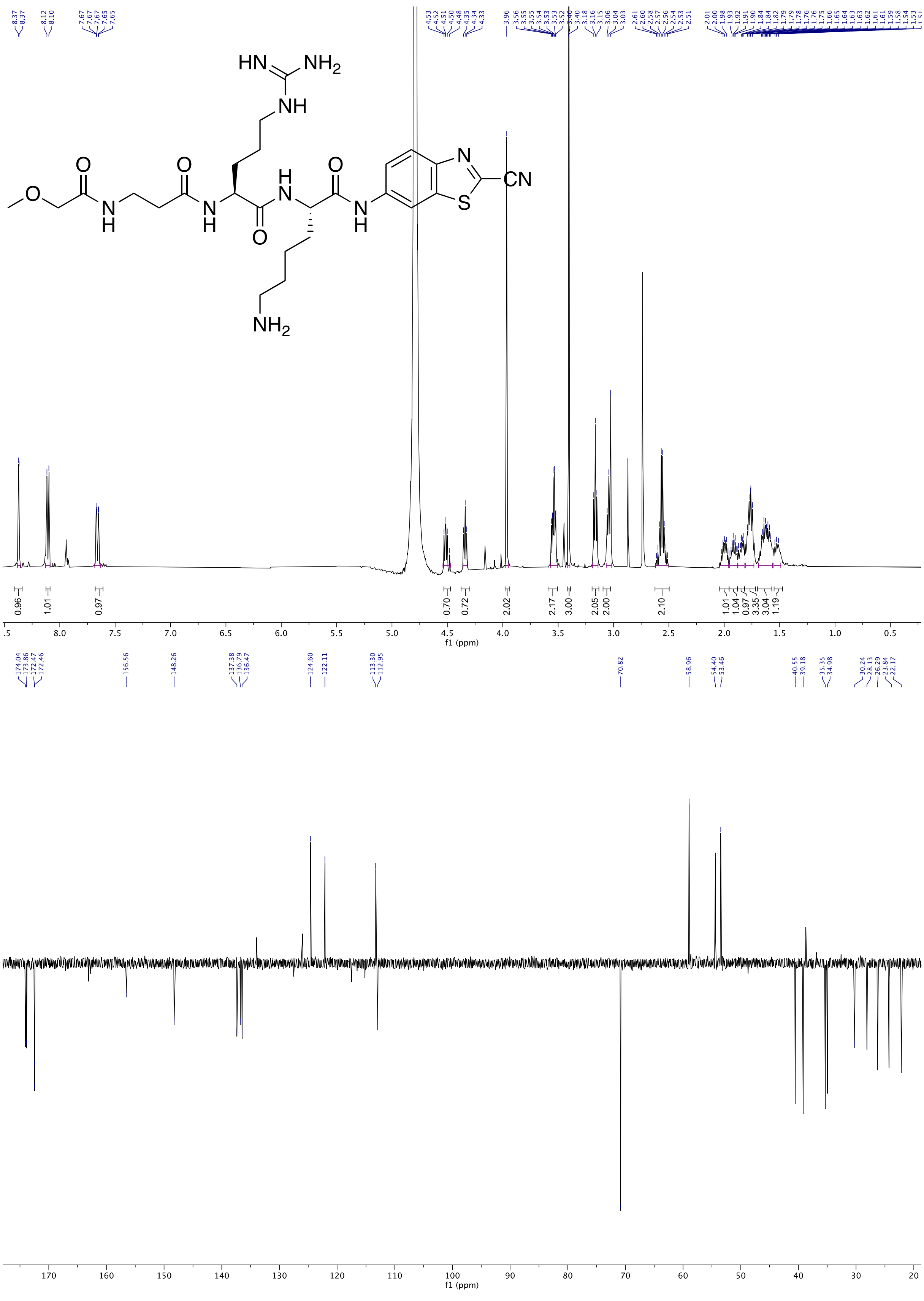

Figure S51: ^{1}H and ^{13}C NMR spectra of methoxyacetyl-βARK-6ABTC.

9. References

1. Hauser, J. R.; Beard, H. A.; Bayana, M. E.; Jolley, K. E.; Warriner, S. L.; Bon, R. S., Economical and scalable synthesis of 6-amino-2-cyanobenzothiazole. *Beilstein J. Org. Chem.* **2016,** *12,* 2019-2025.

2. van Heerde, W. L.; van Geffen, M.; Steeghs, D., Novel chemiluminescent substrates for Factor Xa. US20210371461A1: 2021.

3. Grimmel, H.; Guenther, A.; Morgan, J. F., Phosphazo compounds and their use in preparing amides. *J. Am. Chem. Soc.* **1946,** *68*, 539-542.

4. Musiol, H.-J.; Moroder, L., N, N ‘-Di-tert-butoxycarbonyl-1 H-benzotriazole-1-carboxamidine Derivatives Are Highly Reactive Guanidinylating Reagents. *Org. Lett.* **2001,** *3*, 3859-3861.

5. Tian, G.; Fedoseev, P.; Van der Eycken, E. V., Hypervalent Iodine (III)-Mediated Cascade Cyclization of Propargylguanidines and Total Syntheses of Kealiinine B and C. *Chem. Eur. J.* **2017,** *23,* 5224-5227.
